# Combining the strengths of inverse-variance weighting and Egger regression in Mendelian randomization using a mixture of regressions model

**DOI:** 10.1371/journal.pgen.1009922

**Published:** 2021-11-18

**Authors:** Zhaotong Lin, Yangqing Deng, Wei Pan

**Affiliations:** Division of Biostatistics, University of Minnesota, Minneapolis, Minnesota, United States of America; University of Cambridge, UNITED KINGDOM

## Abstract

With the increasing availability of large-scale GWAS summary data on various traits, Mendelian randomization (MR) has become commonly used to infer causality between a pair of traits, an exposure and an outcome. It depends on using genetic variants, typically SNPs, as instrumental variables (IVs). The inverse-variance weighted (IVW) method (with a fixed-effect meta-analysis model) is most powerful when all IVs are valid; however, when horizontal pleiotropy is present, it may lead to biased inference. On the other hand, Egger regression is one of the most widely used methods robust to (uncorrelated) pleiotropy, but it suffers from loss of power. We propose a two-component mixture of regressions to combine and thus take advantage of both IVW and Egger regression; it is often both more efficient (i.e. higher powered) and more robust to pleiotropy (i.e. controlling type I error) than either IVW or Egger regression alone by accounting for both valid and invalid IVs respectively. We propose a model averaging approach and a novel data perturbation scheme to account for uncertainties in model/IV selection, leading to more robust statistical inference for finite samples. Through extensive simulations and applications to the GWAS summary data of 48 risk factor-disease pairs and 63 genetically uncorrelated trait pairs, we showcase that our proposed methods could often control type I error better while achieving much higher power than IVW and Egger regression (and sometimes than several other new/popular MR methods). We expect that our proposed methods will be a useful addition to the toolbox of Mendelian randomization for causal inference.

## Author summary

For causal inference, inverse-variance weighting (IVW) and Egger regression are two of the most widely applied Mendelian randomization methods nowadays. IVW is the most powerful under the perhaps too restrictive assumption that all IVs are valid, while Egger regression is often unnecessarily too flexible in assuming all IVs to be invalid with uncorrelated pleiotropic effects. In spite of their usefulness, we point out their limitations: an IVW estimate of a causal effect would be biased if some/all IVs have directional pleiotropic effects, and an Egger regression estimate has too large a variance, leading to its loss of power. Accordingly we propose a mixture model to combine them to take advantage of their strengths while overcoming their major limitations. Furthermore, we propose a model-averaging approach and a novel data perturbation scheme to account for uncertainties in model/IV selection, leading to more robust statistical inference. Through simulations and applications to some publicly available large-scale GWAS summary data, we demonstrate the superiority of our methods over IVW and Egger regression (and over some other state-of-the-art MR methods in some scenarios).

## Introduction

**Fig 1:**
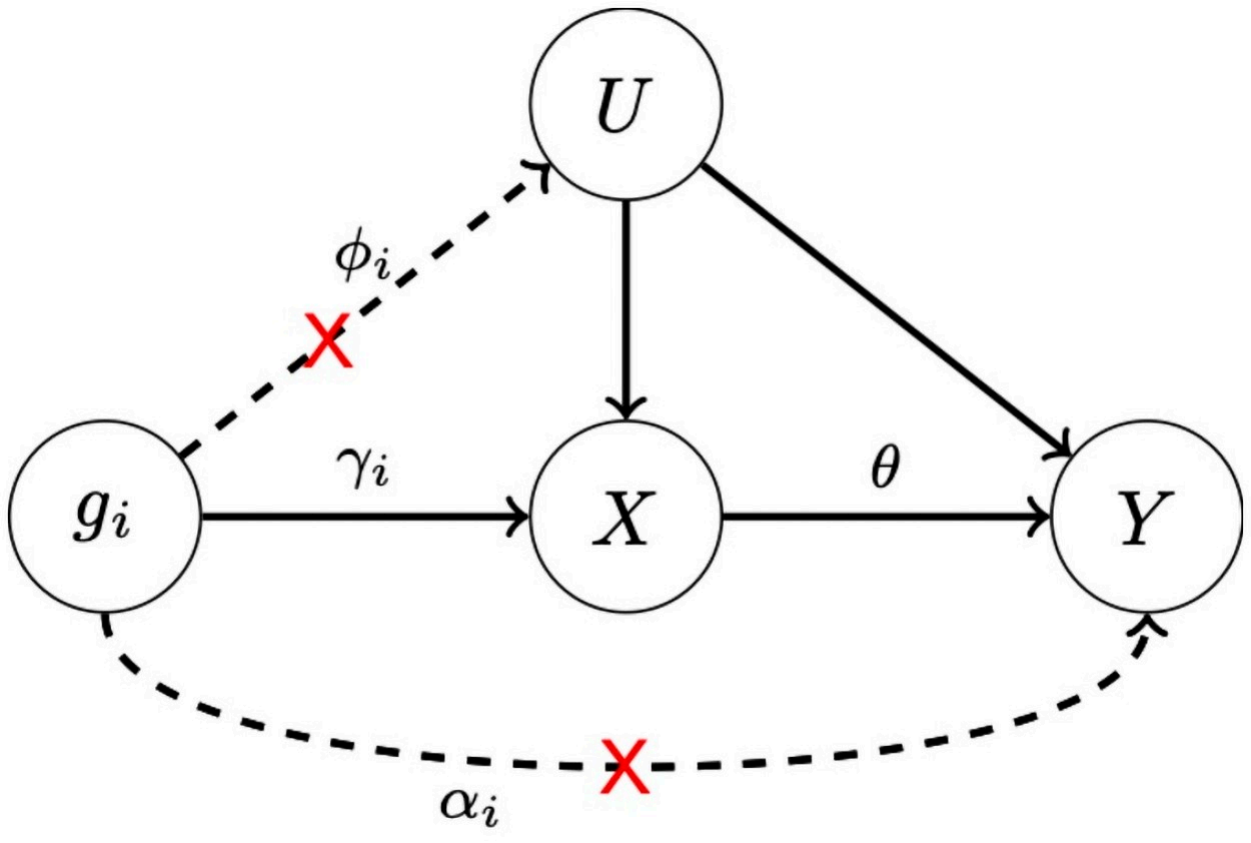
A causal diagram illustrating the three assumptions on a valid IV *g*_*i*_. Dashed lines (which are marked with a red ‘cross’) correspond to violations of assumptions 2 and 3.

Mendelian randomization (MR) has become a widely used technique to infer causal relationship between an exposure (e.g. a risk factor) and an outcome (e.g. a disease) using GWAS summary data, in which usually independent genetic variants (SNPs) are used as instrument variables (IVs) [1–3]. To guarantee correct inference, as shown in Fig 1, a valid IV used in MR must be:

1. associated with the exposure (*X*), i.e. *γ*_*i*_ ≠ 0;
2. not associated with the outcome (*Y*) conditional on the exposure (*X*) and hidden confounder (*U*), i.e. *α*_*i*_ = 0;
3. not associated with any hidden confounder (*U*), i.e. *ϕ*_*i*_ = 0.

When all IVs used in Mendelian randomization are valid (and uncorrelated), the inverse-variance weighted (IVW) method is consistent and most powerful: it combines the IV-specific ratio estimates most efficiently by inverse-variance weighting [4]. However, in the presence of horizontal pleiotropy, where some or all IVs have direct effects on the outcome, the second IV assumption is violated and IVW may not be consistent unless the mean of the direct effects is zero, a scenario of so-called “balanced pleiotropy”. More generally, Egger regression is applied under a weaker assumption that the direct or pleiotropic effects of the genetic variants on the outcome are independent of the genetic associations with the exposure (so-called InSIDE assumption) [5]. It has been noted recently that Egger regression often suffers from a severe loss of power, because it assumes that all IVs are invalid, which may be too extreme. Many other methods have been proposed to deal with the violation of Assumption 2 or both Assumptions 2 and 3, but most of them require either the plurality or the majority assumption [6–9]. We note that Egger regression is the only method allowing all IVs to be invalid (with possibly directional pleiotropy), and is easy to apply, both of which perhaps explain its popularity.

Both IVW and Egger regression, as two of the most popular MR methods, impose too extreme assumptions: while IVW (fixed effects) assumes that all IVs are valid, both IVW (random effects) and Egger regression assume that all IVs are invalid; the truth is perhaps often between the two. We acknowledge and take account of the possibility of having from zero to all invalid IVs. Accordingly, we propose a two-component mixture of regressions model, denoted **mixIE**, which can be viewed as the mixture of IVW (fixed effects) and Egger regression, and thus may be more efficient and more robust to the violation of IV Assumption 2; as Egger regression, the proposed new method requires the IV Assumption 3 (so that InSIDE) to hold, though we will show that it is more robust than Egger regression when the assumption is violated. The model is fitted using a classification expectation-maximization (CEM) algorithm [10, 11], selecting valid and invalid IVs to be used by IVW (fixed effects) and Egger regression respectively. To account for uncertainties in model/IV selection, we propose a model-averaging approach based on a few top selected models, in addition to the default IVW (fixed effects) and Egger regression models. Furthermore, we propose a novel data perturbation scheme to deal with more challenging situations where the true model is only weakly identifiable, e.g. with many IVs having weak pleiotropic effects; it controls the type I error better in these challenging situations.

There are two other related methods based on mixture models as well, namely MR-ContMix [12] and MRMix [13]. MRMix assumes a four-component normal mixture model for the underlying bivariate effect size distribution of all the SNPs for a pair of traits, and MR-ContMix assumes a two-component normal mixture model for the ratio estimates (corresponding to valid and invalid IVs), while our proposed method is based on a mixture of *regressions* model for GWAS summary statistics directly. All three mixture models include a component for valid IVs and could potentially identify valid and invalid IVs. Another novel and powerful method based on constrained maximum likelihood (cML) also selects and (implicitly) removes invalid IVs by estimating the pleiotropic effect (if it exists) for each SNP and then only uses valid IVs for statistical inference [14]. However, [14] shows in their simulations that although MR-ContMix performed well in most scenarios, sometimes it performed poorly probably due to the challenge of its pre-selection of a fixed tuning parameter. At the same time, MRMix often performed well with mostly well controlled type 1 errors and high power, but it might have either largely inflated or too conservative type 1 errors while giving biased estimates. Here we will further compare mixIE with cML, MR-ContMix and MRMix in our numerical studies. At last, MRMix, MR-ContMix and cML all require the valid IV plurality condition, while mixIE, as Egger regression, could potentially handle situations when all IVs are invalid but requiring the InSIDE assumption to hold; we also show that mixIE is more robust than Egger regression when the InSIDE assumption is violated.

The rest of the paper is organized as follows. We first introduce mixIE and its variants based on model averaging and data perturbation. We then show the advantages of our proposed methods over IVW and Egger regression through extensive simulations. Finally we apply and compare various methods on 48 risk factor-disease pairs and 63 trait pairs using multiple publicly available GWAS summary datasets.

## Methods

### Overview

We consider the setup of two-sample Mendelian randomization. Suppose that we have *m* independent SNPs, *G*_1_, …, *G*_*m*_, as IVs, an exposure *X*, an outcome *Y*, and some hidden confounder *U*; *θ* is the causal effect of the exposure on the outcome and is the parameter of interest. The coefficient vector ***γ***_*xg*_ represents the associations between SNPs and the exposure, and ***α***_*yg*_ the direct effects of SNPs on the outcome that are not mediated through the exposure. The true model is

$$\begin{array}{cc} X & =G\gamma_{xg}+U+e_{x},\\ Y & =\theta X+G\alpha_{yg}+U+e_{y}. \end{array}$$ (1)

where **X**, **Y** and **G** are the vectors for the observed exposure, outcome and genotypic scores respectively, and **e**_*x*_ and **e**_*y*_ are the vectors for independent errors.

Given two independent GWAS summary datasets of traits *X* and *Y* with sample sizes *n*_*x*_ and *n*_*y*_ respectively, we extract the data for *m* independent SNPs that are significantly associated with *X*; that is, their marginal association parameter and variance estimates, $\left({\hat{\beta }}_{Xi},{\hat{\sigma }}_{Xi}^{2}\right)$ and $\left({\hat{\beta }}_{Yi},{\hat{\sigma }}_{Yi}^{2}\right)$, *i* = 1, …, *m*. The IVW model is

$$\begin{array}{c} {\hat{\beta }}_{Yi}=\theta {\hat{\beta }}_{Xi}+\epsilon_{Ii};\;\epsilon_{Ii}∼N\left(0,\sigma_{I}^{2}{\hat{\sigma }}_{Yi}^{2}\right). \end{array}$$

Fitting the above model using weighted least squares lead to the IVW estimate ${\hat{\theta }}_{IVW}$, which is consistent for *θ* if all IVs are valid or have balanced pleiotropy. If all IVs are valid, $\sigma_{I}^{2}=1$, which is specified under the fixed-effect (FE) IVW; on the other hand, if some IVs are invalid but have balanced pleiotropy, $\sigma_{I}^{2}>1$ is estimated under the random-effect (RE) IVW. Note that IVW(FE) and IVW(RE) assume that all IVs are valid and invalid respectively. Throughout this paper, if IVW is used alone (as a single MR method), it always refers to its RE version; however, in our proposed mixIE, IVW(FE) is always used.

Egger regression is a simple modification to the above linear model without constraining the intercept to be zero:

$$\begin{array}{c} {\hat{\beta }}_{Yi}=r+\theta {\hat{\beta }}_{Xi}+\epsilon_{Ei};\;\epsilon_{Ei}∼N\left(0,\sigma_{E}^{2}{\hat{\sigma }}_{Yi}^{2}\right). \end{array}$$ (2)

As pleiotropic effects of genetic variants will lead to overdispersion, a random-effects analysis should always be preferred by estimating $\sigma_{E}^{2}\geq 1$ in Egger regression [15]. Under the InSIDE assumption, the Egger estimate ${\hat{\theta }}_{E}$ of *θ* is consistent (as both the sample size and the number of genetic variants tend to infinity).

To take the advantage of both IVW and Egger regression, we propose a two-component mixture regression model for IVW and Egger regression respectively, called **mixIE** for short. The two components correspond to modeling valid IVs and invalid IVs respectively. If we know which IVs are valid and which are invalid, we would use the corresponding IVW(FE) and Egger regression models respectively. In practice, since we do not know, we have a mixture of two regressions and the log-likelihood function (up to a constant) as

$$\begin{array}{cc} l(\theta ,r,c,\pi ;\left\{{\hat{\beta }}_{Xi},{\hat{\beta }}_{Yi},{\hat{\sigma }}_{Yi}^{2}\right\}) & =\sum_{i=1}^{K}-\frac{1}{2}[\frac{{\left({\hat{\beta }}_{Yi}-\theta {\hat{\beta }}_{Xi}-r\right)}^{2}}{c{\hat{\sigma }}_{Yi}^{2}}+\text{log}\left(c{\hat{\sigma }}_{Yi}^{2}\right)]\\ & +\sum_{i=K+1}^{m}-\frac{1}{2}[\frac{{\left({\hat{\beta }}_{Yi}-\theta {\hat{\beta }}_{Xi}\right)}^{2}}{{\hat{\sigma }}_{Yi}^{2}}+\text{log}\left({\hat{\sigma }}_{Yi}^{2}\right)], \end{array}$$ (3)

where, among the unknown parameters, *r* is the average pleiotropic effect of invalid IVs, *c* is the multiplicative over-dispersion parameter, and *π* = *K*/*m* is the proportion of invalid IVs; we use $\left\{{\hat{\beta }}_{Xi},{\hat{\beta }}_{Yi},{\hat{\sigma }}_{Yi}^{2}\right\}$ to represent the observed data. Here for simplicity of notation, we denote the first *K* SNPs, *G*_1_, …, *G*_*K*_, as invalid IVs, and the remaining *G*_*K*+1_, …, *G*_*m*_ as valid IVs. In practice, we do not know which IVs are invalid (and all the parameter values), thus we apply the classification expectation-maximization (CEM), a variant of EM algorithm, to classify IVs (and estimate the parameters) [10, 11]. Since the result of EM or CEM depends on the choice of starting values and there may be multiple models fitting the data well, we propose a model averaging approach, called **mixIE-MA**. We also propose a data perturbation strategy to further take account of the uncertainty in model/IV selection, called **mixIE-MA-DP** later.

### Model fitting

The formulation of our approach lends itself to the classification EM algorithm [10]. Let *z*_*i*_ denote the unobserved indicator of whether IV/SNP *i* being invalid or not. For convenience, let ***α*** = (*θ*, *r*, *c*, *π*) denote the set of all unknown parameters, and $D_{i}=\left({\hat{\beta }}_{Xi},{\hat{\beta }}_{Yi},{\hat{\sigma }}_{Yi}^{2}\right)$ the observed data for SNP *i*. The (*t* + 1)th iteration of CEM algorithm is defined as follows:

- E-step: calculate
  $$\begin{array}{cc} \tau_{i,0}^{\left(t+1\right)} & ≔P(z_{i}=0|D_{i};\alpha^{\left(t\right)})\\ & =\frac{f\left({\hat{\beta }}_{Yi}-\theta^{\left(t\right)}{\hat{\beta }}_{Xi},{\hat{\sigma }}_{Yi}^{2}\right)\cdot \left(1-\pi^{\left(t\right)}\right)}{f\left({\hat{\beta }}_{Yi}-\theta^{\left(t\right)}{\hat{\beta }}_{Xi},{\hat{\sigma }}_{Yi}^{2}\right)\cdot \left(1-\pi^{\left(t\right)}\right)+f\left({\hat{\beta }}_{Yi}-\theta^{\left(t\right)}{\hat{\beta }}_{Xi}-r^{\left(t\right)},c^{\left(t\right)}{\hat{\sigma }}_{Yi}^{2}\right)\cdot \pi^{\left(t\right)}},\\ \tau_{i,1}^{\left(t+1\right)} & ≔P\left(z_{i}=1|D_{i};\alpha^{\left(t\right)}\right)=1-\tau_{i,0}^{\left(t+1\right)}, \end{array}$$
  where *f*(*a*, *σ*^2^) is the density function value at *a* for $N(0,\sigma^{2})$.
- C-step: classify SNP *i* as invalid IV if $\tau_{i,1}^{\left(t+1\right)}\;⩾\;0.5$, otherwise as valid IV, and let $\hat{K}$ denote the number of the classified invalid IVs. Again, for simplicity of notation, after possible rearrangement of the orders of the SNPs, we denote the first $\hat{K}$ SNPs as invalid IVs and the rest $m-\hat{K}$ as valid IVs.
- M-step: update the parameter estimates
  $$\begin{array}{cc} \pi^{\left(t+1\right)} & =\frac{\hat{K}}{m},\\ r^{\left(t+1\right)} & =\frac{\sum_{i=1}^{\hat{K}}\frac{{\hat{\beta }}_{Yi}-\theta^{\left(t\right)}{\hat{\beta }}_{Xi}}{{\hat{\sigma }}_{Yi}^{2}}}{\sum_{i=1}^{\hat{K}}\frac{1}{{\hat{\sigma }}_{Yi}^{2}}},\\ c^{\left(t+1\right)} & =\frac{\sum_{i=1}^{\hat{K}}\frac{{\left({\hat{\beta }}_{Yi}-\theta^{\left(t\right)}{\hat{\beta }}_{Xi}-r^{\left(t+1\right)}\right)}^{2}}{{\hat{\sigma }}_{Yi}^{2}}}{\hat{K}},\\ \theta^{\left(t+1\right)} & =\frac{\sum_{i=1}^{\hat{K}}\frac{\left({\hat{\beta }}_{Yi}-r^{\left(t+1\right)}\right){\hat{\beta }}_{Xi}}{c^{\left(t+1\right)}{\hat{\sigma }}_{Yi}^{2}}+\sum_{i=\hat{K}+1}^{m}\frac{{\hat{\beta }}_{Yi}{\hat{\beta }}_{Xi}}{{\hat{\sigma }}_{Yi}^{2}}}{\sum_{i=1}^{\hat{K}}\frac{{\hat{\beta }}_{Xi}^{2}}{c^{\left(t+1\right)}{\hat{\sigma }}_{Yi}^{2}}+\sum_{i=\hat{K}+1}^{m}\frac{{\hat{\beta }}_{Xi}^{2}}{{\hat{\sigma }}_{Yi}^{2}}}. \end{array}$$

We obtain the final estimates $\left(\hat{\theta },\hat{r},\hat{c},\hat{\pi }\right)=\left(\theta^{\left(t+1\right)},r^{\left(t+1\right)},c^{\left(t+1\right)},\pi^{\left(t+1\right)}\right)$ at the convergence. By default, as in our simulations and real data analyses, we set *r*^(0)^ = 0, *c*^(0)^ = 1, *π*^(0)^ = 0.2 and generate *θ*^(0)^ randomly from $-\text{max}(|{\hat{\beta }}_{Yi}/{\hat{\beta }}_{Xi}|)$ to $\text{max}(|{\hat{\beta }}_{Yi}/{\hat{\beta }}_{Xi}|)$ as well as including 0 and point estimates from IVW and Egger regression. The choice of *π*^(0)^ = 0.2 is because that there is usually a small proportion of invalid IVs in many real data examples as shown later and in [14]. When *r*^(0)^ = 0 and *c*^(0)^ = 1, the two components are the same, thus we run two iterations of the standard EM algorithm before starting the CEM.

To obtain the standard error of the estimated parameters, we take the approach proposed in [16], which requires the computation of the gradients and the information matrix based on the complete data log-likelihood. The details are given in Section A in S1 Text.

### Model averaging

It is well known that the EM algorithm is sensitive to the choice of starting values while there may be multiple good models, therefore we use model averaging to account for this uncertainty. Specifically, we use different starting values to reach a few top candidate models, and by default, we always include the fixed-effect IVW model (i.e. $\hat{\pi }=0,\hat{c}=1,\hat{r}=0$) and the Egger regression model (i.e. $\hat{\pi }=1$) in the list of the top candidate models. A model is judged by its Bayesian information criterion (BIC) [17]:

$$\begin{array}{c} BIC=-2\cdot l\left(\hat{\theta },\hat{r},\hat{c},\hat{\pi };\left\{{\hat{\beta }}_{Xi},{\hat{\beta }}_{Yi},{\hat{\sigma }}_{Yi}^{2}\right\}\right)+\text{log}\left(n_{y}\right)\cdot \left(2+1\left\{\hat{r}\neq 0\right\}+1\left\{\hat{c}>1\right\}\right), \end{array}$$

where the indicator function 1{A}=1 or 0, depending on whether *A* is true or not. Based on the BIC values of the various fitted models, we select up to 5 top models. Following [18], we define the weight for model *k* = 1, …, 5 with BIC value *BIC*_*k*_ as

$$\begin{array}{c} w_{k}=\frac{\text{exp}(-BIC_{k}/2)}{\sum_{j=1}^{5}\text{exp}(-BIC_{j}/2)}. \end{array}$$

Now we combine the estimates ${\hat{\theta }}_{k}$ from candidate models *k* = 1, …, 5 to have the final model-averaging estimate and its standard error as

$$\begin{array}{cc} {\hat{\theta }}_{MA} & =\sum_{k}w_{k}{\hat{\theta }}_{k},\\ SE({\hat{\theta }}_{MA}) & =\sum_{k}w_{k}\sqrt{SE{\left({\hat{\theta }}_{k}\right)}^{2}+{\left({\hat{\theta }}_{k}-{\hat{\theta }}_{MA}\right)}^{2}}. \end{array}$$

### Data perturbation

To further take account of the uncertainty in model selection/averaging, we propose a data perturbation strategy [19]. Instead of applying the usual asymptotics that ignores the uncertainty in model selection/averaging, we perturb the data to mimic generating multiple samples from the data distribution. With each perturbed/generated sample, we repeat an estimation procedure (as applied to the original data) so that the uncertainty in model selection/averaging or other aspects can be taken account when, at the end, the empirical distribution of such estimates from multiple perturbed samples is used for inference. This is similar to and can even trace back to the little bootstrap method [20] in the context of model selection. We found that the (more general) data perturbation scheme proposed in [14] did not perform well for mixIE in some extreme situations (presumably because of the nature of Egger regression with the use of invalid IVs), so we propose a modified one for mixIE specifically.

Since mixIE classifies IVs as valid or invalid, estimating the causal effect $\hat{\theta }$ could be approximated via a fixed effect meta analysis of IVW estimate ${\hat{\theta }}_{I}$ and Egger estimate ${\hat{\theta }}_{E}$ based on the sets of valid IVs and of invalid IVs respectively:

$$\begin{array}{c} \hat{\theta }=(\frac{{\hat{\theta }}_{I}}{SE{\left({\hat{\theta }}_{I}\right)}^{2}}+\frac{{\hat{\theta }}_{E}}{SE{\left({\hat{\theta }}_{E}\right)}^{2}})/(\frac{1}{SE{\left({\hat{\theta }}_{I}\right)}^{2}}+\frac{1}{SE{\left({\hat{\theta }}_{E}\right)}^{2}}). \end{array}$$ (4)

Similarly for mixIE-MA, we could classify IVs based on their model averaged posterior probabilities. Accordingly, we propose the following data perturbation scheme for mixIE-MA:

Step 1: Perturb the observed data by ${\hat{\beta }}_{Yi}^{\left(b\right)}={\hat{\beta }}_{Yi}+\epsilon_{i}^{*}$, where $\epsilon_{i}^{*}∼N\left(0,{\hat{\sigma }}_{Yi}^{2}\right)$, and ${\hat{\sigma }}_{Yi}^{\left(b\right)}=\sqrt{\text{var}({\hat{\beta }}_{Yi}^{\left(b\right)})}=\sqrt{2}\cdot {\hat{\sigma }}_{Yi}$.
Step 2: Apply mixIE-MA algorithm on the *b*-th perturbed dataset $\left({\hat{\beta }}_{Xi},{\hat{\beta }}_{Yi}^{\left(b\right)},{\hat{\sigma }}_{Yi}^{\left(b\right)}\right)$ and obtain the corresponding estimated sets of valid IVs and invalid IVs. The estimated causal effect ${\hat{\theta }}^{\left(b\right)}$ could be approximated by Eq (4) with (${\hat{\theta }}_{I}^{\left(b\right)},SE\left({\hat{\theta }}_{I}^{\left(b\right)}\right)$) and (${\hat{\theta }}_{E}^{\left(b\right)},SE\left({\hat{\theta }}_{E}^{\left(b\right)}\right)$) estimated from IVW and Egger regression respectively.
Step 3: Further perturb the data for the set of invalid IVs by ${\hat{\beta }}_{Yi}^{(b)*}={\hat{\beta }}_{Yi}+{\hat{\sigma }}^{\left(b\right)}\epsilon_{i}^{*}$, where ${\hat{\sigma }}^{\left(b\right)}$ is the estimated inflation factor in Egger regression model (2) for the set of invalid IVs identified above.
Step 4: Apply Egger regression to the new perturbed data ${\hat{\beta }}_{Yi}^{(b)*}$ and obtain ${\hat{\theta }}_{E}^{(b)*}$.
Step 5: Apply fixed-effect meta analysis to combine ${\hat{\theta }}_{I}^{\left(b\right)}$ and ${\hat{\theta }}_{E}^{(b)*}$, obtaining ${\hat{\theta }}^{(b)*}$ for the *b*-th perturbed dataset as follows:
  $$\begin{array}{c} {\hat{\theta }}^{(b)*}=(\frac{{\hat{\theta }}_{I}^{\left(b\right)}}{SE{\left({\hat{\theta }}_{I}^{\left(b\right)}\right)}^{2}}+\frac{{\hat{\theta }}_{E}^{(b)*}}{SE{\left({\hat{\theta }}_{E}^{\left(b\right)}\right)}^{2}})/(\frac{1}{SE{\left({\hat{\theta }}_{I}^{\left(b\right)}\right)}^{2}}+\frac{1}{SE{\left({\hat{\theta }}_{E}^{\left(b\right)}\right)}^{2}}). \end{array}$$

We repeat the above steps *B* times and obtain the mean and standard deviation of ${\left\{{\hat{\theta }}^{(b)*}\right\}}_{b=1}^{B}$ as the final point estimate and standard error for mixIE-MA-DP. We could also use the proportion of the times when SNP *i* being identified as invalid out of the *B* data perturbations as the posterior probability estimate ${\hat{\tau }}_{i,1}$ for mixIE-MA-DP. By default we used *B* = 200 in our simulations and real data analysis.

It is noted that the DP on GWAS *summary* data is equivalent to the bootstrap on the corresponding *individual-level* data. Suppose the GWAS individual level data are {(*Y*_*j*_, *Z*_*ij*_): *j* = 1, 2, …, *n*} for outcome *Y* and SNP/IV *i* (both centered at mean 0). For model *Y*_*j*_ = *Z*_*ij*_ *β*_*Yi*_ + *e*_*Yij*_ with iid $e_{Yij}∼N(0,\sigma_{ei}^{2})$, we obtain the ordinary least square (OLS), or equivalently maximum likelihood, estimate ${\hat{\beta }}_{Yi}∼N\left(\beta_{Yi},\sigma_{Yi}^{2}\right)$. If we apply the parametric bootstrap by generating $Y_{j}^{\left(b\right)}=Z_{ij}{\hat{\beta }}_{Yi}+e_{Yij}^{\left(b\right)}$ with iid $e_{Yij}^{\left(b\right)}∼N\left(0,\sigma_{ei}^{2}\right)$, it is easy to verify that, conditional on ${\hat{\beta }}_{Yi}$, the corresponding OLS estimate ${\hat{\beta }}_{Yi}^{\left(b\right)}∼N\left({\hat{\beta }}_{Yi},\sigma_{Yi}^{2}\right)$, the same distribution (with $\sigma_{Yi}^{2}$ replaced by its estimate ${\hat{\sigma }}_{Yi}^{2}$) used in Step 1 in our DP procedure. In fact, based on the results in [21] (Section 7.2.2), the conclusion holds (asymptotically) for other three types of the bootstrap: residual bootstrap (by resampling residuals), nonparametric bootstrap (by resampling pairs (*Y*_*j*_, *Z*_*ij*_)) and external/wild/multiplier bootstrap.

### Goodness-of-fit testing

In mixIE, invalid IVs are modeled according to Egger regression, which requires the InSIDE assumption. Thus, in principle mixIE and the proposed data perturbation scheme would also require the InSIDE assumption to hold. On the other hand, it is known that the InSIDE assumption is difficult to test [22–24]. A general way for model checking is to compare our proposed model with some other methods that do not require the InSIDE assumption, such as cML, MRMix and MR-ContMix, though it only works if other assumptions for the latter methods hold (e.g. the plurality of valid IV assumption). Specifically, we evaluate the consistency between our estimates with that of another method; any inconsistency might be due to the violation of the InSIDE or other assumptions of the two methods being compared. Here we call it goodness-of-fit (GOF) testing, though it is perhaps more in line with and can be used for triangulation [25].

To compare two different methods on a given dataset, one has to account for the correlation between the two estimates from the two methods, which is not trivial. We propose using a general data perturbation scheme similar to that of [14] (but under no measurement error (NOME) assumption as adopted by IVW and Egger regression, thus by mixIE) for such a purpose. Specifically, we propose the following procedure: Staring with *b* = 1,

Step 1: Perturb the data to generate ${\hat{\beta }}_{Yi}^{\left(b\right)}={\hat{\beta }}_{Yi}+\epsilon_{Yi}^{*}$ with $\epsilon_{Yi}^{*}∼N\left(0,{\hat{\sigma }}_{Yi}^{2}\right)$ independently;
Step 2: Apply mixIE-MA and another method, such as cML-MA, to the perturbed dataset $\left({\hat{\beta }}_{Xi},{\hat{\beta }}_{Yi}^{\left(b\right)},{\hat{\sigma }}_{Yi}^{2}\right)$ and obtain the corresponding $\theta_{m}^{\left(b\right)}$ and $\theta_{c}^{\left(b\right)}$;
Step 3: Calculate their difference, $\delta^{\left(b\right)}=\theta_{m}^{\left(b\right)}-\theta_{c}^{\left(b\right)}$, and let *b* ← *b* + 1.

We repeat the above steps for *B* = 200 times and obtain the empirical distribution of ${\left\{\delta^{(b)*}\right\}}_{b=1}^{B}$. If the 95% percentile interval of {*δ*^(*b*)*^} does not cover 0, then we conclude that the results of mixIE-MA and the other method (not requiring InSIDE) are inconsistent with each other, suggesting possible violation of the InSIDE assumption for mixIE, or of some other assumptions required by the two methods. In such a case, cautions should be taken in interpreting the causal results.

### Other MR methods

We compared mixIE-MA and mixIE-MA-DP with other popular two-sample MR methods, including random-effect IVW model, Egger regression, MR-Mix [13], MR-ContMix [12], weighted-median [6] and a new method called constrained maximum likelihood (cML) [14]. We applied cML with its model averaging version based on BIC, called cML-MA for short, and its data perturbation version, called cML-MA-DP.

### GWAS data

#### Primary real data example

We applied our proposed methods and other MR methods to 48 pairs of risk factor-disease GWAS summary data following [26], including 12 risk factors and 4 diseases. For each risk factor-disease pair, we used the set of LD-independent SNPs as IVs as described in [26] (in their S4 Table), and applied all methods to the GWAS summary statistics of these SNPs.

#### Secondary real data example

Following [14], we also applied our proposed methods to 63 trait pairs whose genetic correlations are not significant, suggesting that they are unlikely to be causally related. For each pair, we used the code provided in its Supplementary to extract the GWAS summary statistics for analysis.

### Simulation set-ups

#### Main simulations

Similar to the set-ups in [27], we simulated data according to Eq (5),

$$\begin{array}{cc} U & =G\phi_{ug}+e_{u},\\ X & =G\gamma_{xg}+U+e_{x},\\ Y & =\theta X+G\alpha_{yg}+U+e_{y}, \end{array}$$ (5)

in which

1. the genotype scores of *m* SNPs/IVs (**G**) were generated independently from Binomial(2,*f*_*i*_), where for each SNP *i* its MAF *f*_*i*_ was generated independently from a uniform distribution U(0.1,0.3);
2. the IV strengths ***γ***_*xg*_ were generated from a left-truncated normal distribution: for *m* = 10, an IV strength was generated from $N(0,0.15^{2})$ left-truncated at 0.15 (i.e. any value generated would be larger than 0.15); for *m* = 30, it was generated from $N(0,0.1^{2})$ left-truncated at 0.1; for *m* = 100, it was from $N(0,0.05^{2})$ left-truncated at 0.05;
3. for *K* = *m* ⋅ (p_invalid) invalid IVs, we consider three scenarios:
  (a) Balanced pleiotropy and InSIDE satisfied, where pleiotropy effects ***α***_*yg*_ were generated independently from $N(0,{\sqrt{0.15}}^{2})$ and *ϕ_ug_* = 0;
  (b) Directional pleiotropy and InSIDE satisfied, where ***α***_*yg*_ were generated from $N(0.1,{\sqrt{0.075}}^{2})$ and *ϕ_ug_* = 0;
  (c) Directional pleiotropy and InSIDE violated, where ***α***_*yg*_ were generated from $N(0.1,{\sqrt{0.075}}^{2})$ and *ϕ_ug_* were generated from U(0,b);
4. **e**_*u*_, **e**_*x*_, **e**_*y*_ were generated from N(0,1) independently.

The summary data for genetic associations were calculated for the exposure and the outcome on non-overlapping sets of individuals, each consisting of *n* individuals. For scenarios (a) and (b), we varied *θ* from {0,0.2}, p_invalid from {0, 0.3, 0.5, 0.7, 1} and sample size *n*_*x*_ = *n*_*y*_ = *n* = 10 000 or 50 000. To further compare the power, we also tried a smaller effect size *θ* from {±0.01, ±0.05, ±0.1, ±0.15}, p_invalid from {0.3,0.5,0.7} for *m* = 30 and *n* = 50 000. For scenario (c), we considered different correlated pleiotropy effects by varying *b* from {0.1,0.4,0.7}.

As one reviewer suggested, we also considered different sample sizes for the exposure and the outcome. Details are given in Section B.1.4 in S1 Text.

#### Secondary simulations with weak invalid IVs

Following [14], we simulated data with many invalid IVs with weak effects, called “weak invalid IVs” throughout. We generated *m* = 50 IVs with 60% invalid IVs and with sample size *n* = 20 000. The IV strengths *γ*_*i*_’s were generated independently from N(0,0.01). Pleiotropic effects *α*_*i*_’s for the first 30 IVs were generated independently from $N(0,h_{y}/m)$. Then we set ${\hat{\sigma }}_{Xi}={\hat{\sigma }}_{Yi}=1/\sqrt{n}$ and generated GWAS summary data ${\hat{\beta }}_{Xi}∼N\left(\gamma_{i},{\hat{\sigma }}_{Xi}^{2}\right)$ and ${\hat{\beta }}_{Yi}∼N\left(\theta \cdot \gamma_{i}+\alpha_{i},{\hat{\sigma }}_{Yi}^{2}\right)$, where *θ* is the causal effect of interest. We varied *h*_*y*_ from {0.1,0.2,0.4} and *θ* from {-0.2,-0.1,-0.05,0,0.05,0.1,0.2}.

For each setup, we did 1000 simulations. We compare our proposed methods mixIE-MA and its data perturbation version mixIE-MA-DP with cML-MA, cML-MA-DP, Egger, IVW, MR-Mix, MR-ContMix and weighted-median. For mixIE-MA, mixIE-MA-DP and Egger regression, we used the original coding of SNPs throughout the simulations.

## Results

### Simulations

#### Main simulations

We compared our proposed methods with 6 most popular and new MR methods under three scenarios: InSIDE satisfied, directional pleiotropy; InSIDE satisfied, balanced pleiotropy and InSIDE violated, directional pleiotropy. Here we only show some representative results for *n* = 50000 while all others are given in Section B.1 in S1 Text.

#### InSIDE satisfied

**Fig 2:**
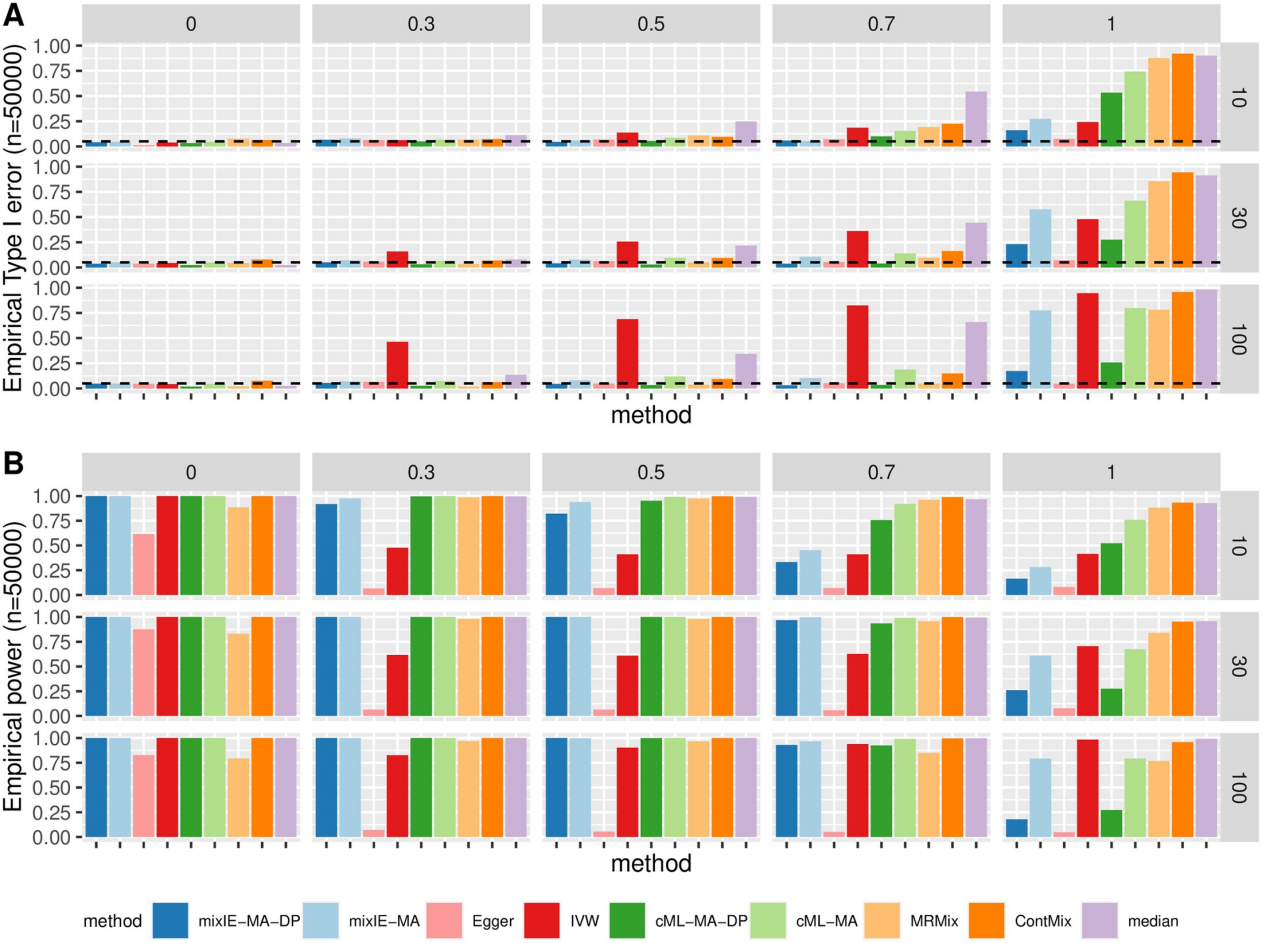
Simulation results with directional pleiotropy and InSIDE satisfied. A: Empirical type-I error; B: Power with sample size *n* = 50 000. Each row corresponds to *m* = 10, 30, 100 SNPs and each column corresponds to 0, 30%, 50%, 70%, 100% invalid IVs.

Fig 2 shows the empirical type 1 error and power of different methods for directional pleiotropy under the InSIDE assumption. First, Egger regression was the only method that could control type 1 error across all scenarios, followed by mixIE-MA-DP and cML-MA-DP that could control type 1 error well except in the more extreme scenarios with all IVs being invalid. But Egger regression also had the lowest power across all scenarios. As expected, IVW had inflated type 1 error in the presence of directional pleiotropy (but not in that of balanced pleiotropy). However, as shown in Section B.1.2 in S1 Text, IVW had very low power in the scenarios of balanced pleiotropy even though it could control the type 1 error, while mixIE-MA-DP was able to control the type 1 error well and had much higher power except when all IVs were invalid. Our proposed method mixIE-MA had inflated type 1 error as the proportion of invalid IVs increased, but this could be improved by mixIE-MA-DP with data perturbation; the latter point was also reflected by cML-MA and cML-MA-DP. MR-Mix was able to control type 1 error in many scenarios except when *m* = 10 and/or all IVs were invalid, and it also performed too conservatively in the cases with all valid IVs. MR-ContMix performed well with 30% invalid IVs but began to have inflated type 1 error when more than 50% IVs were invalid; and weighted-median also had much inflated type 1 error when there were more than half of invalid IVs probably due to the violation of its majority of valid IV assumption. Second, data perturbation was able to further take account of the uncertainty in model selection. We see that mixIE-MA could have inflated type 1 error as the proportion of invalid IVs increased, probably because of incorrectly classifying IVs. In contrast, mixIE-MA-DP was able to control type 1 error in all scenarios but that of p_invalid = 1, and even when all IVs were invalid, it still had much lower type 1 error than the original method. On the other hand, data perturbation might lose some power as compared with the original method, but the loss was acceptable especially when we had a large sample size as shown here. We can see that even when 70% IVs were invalid, there was not much power loss.

**Fig 3:**
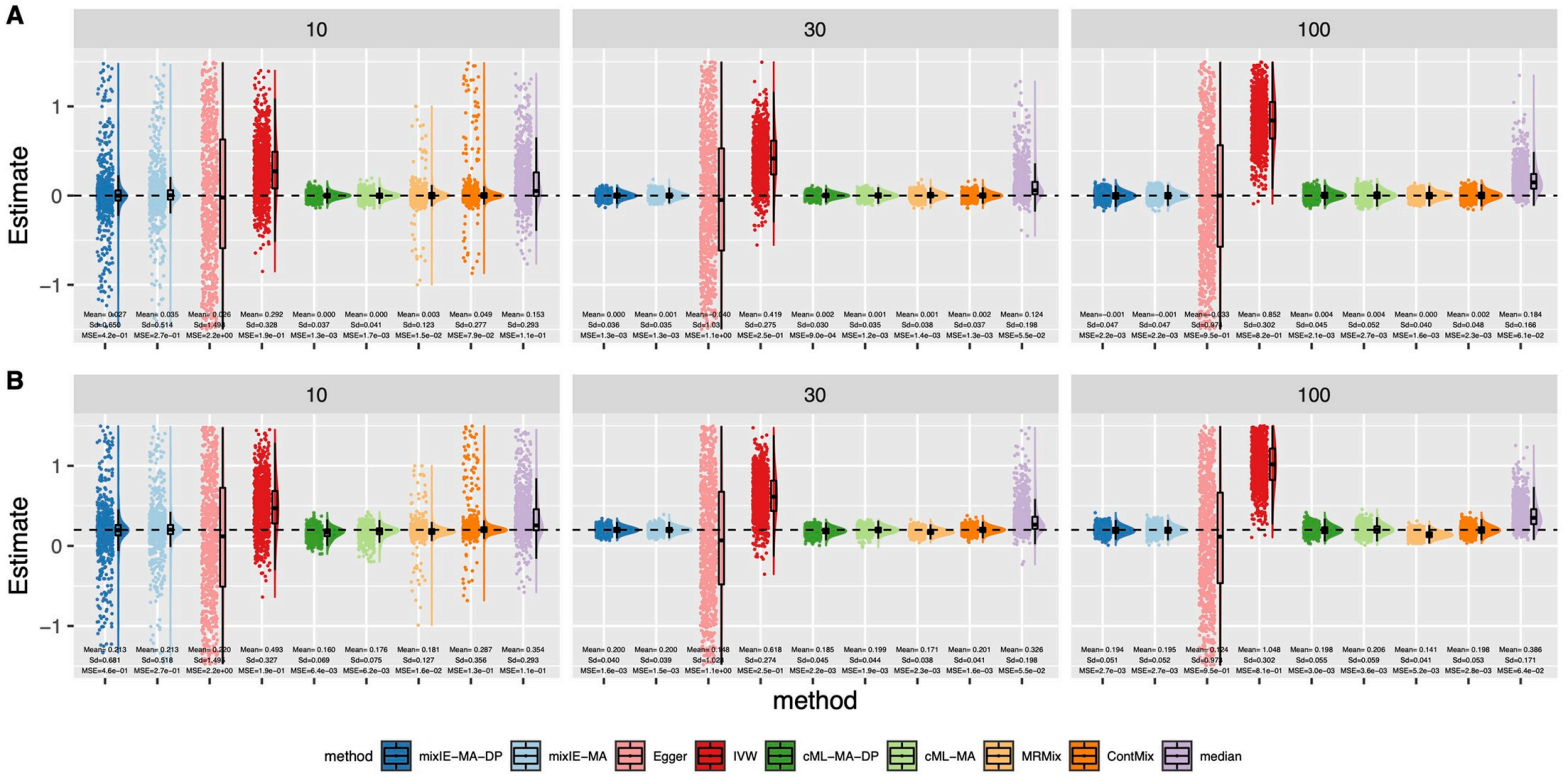
Simulation results with directional pleiotropy and InSIDE satisfied. Empirical distributions of the estimates of the causal effect *θ* by the methods with *n* = 50000 and 70% invalid IVs. A: *θ* = 0. B: *θ* = 0.2.

Fig 3 shows the distributions of the causal parameter estimates by each method when 70% IVs were invalid with directional pleiotropy under InSIDE. In general, cML-MA and cML-MA-DP had the smallest MSE as shown here and in S1 Text. The performance of our proposed methods was less stable with a small number of IVs (i.e. *m* = 10). However, as *m* increased, mixIE-MA-DP had comparable MSEs with that of cML-MA-DP. In addition, as shown in S1 Text, mixIE-MA-DP could have slightly higher power than cML-MA-DP when the sample sizes or effect sizes were small. MR-ContMix also yielded (almost) unbiased estimates with small variances as mixIE and cML methods, while MRMix was slightly biased towards 0 as shown in Fig 3B. And again, Egger regression and IVW gave a very wide range of estimates, and the IVW estimates were biased in the presence of directional pleiotropy.

**Fig 4:**
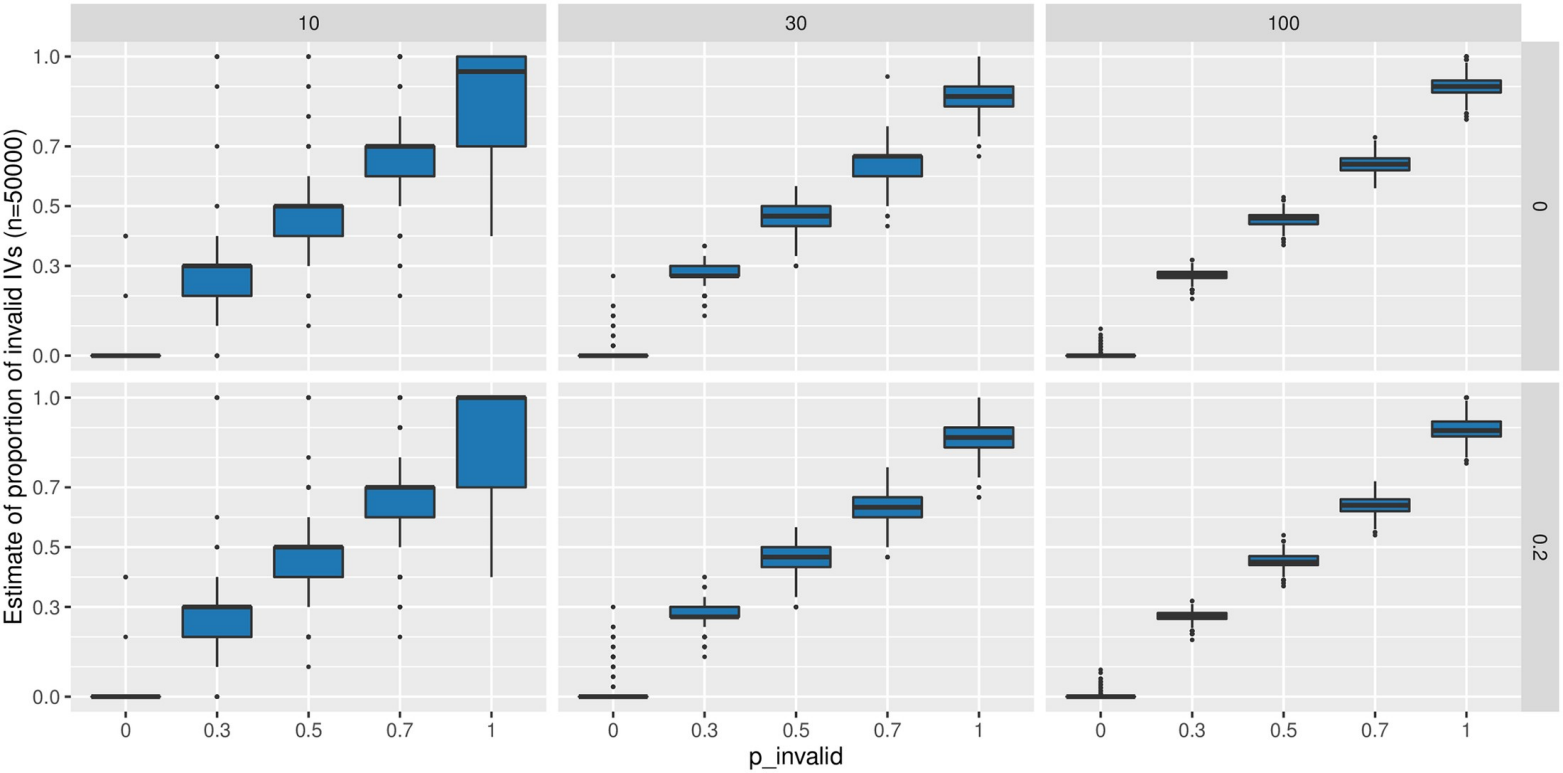
Estimates of the proportion of invalid IVs by mixIE-MA with *n* = 50 000 under directional pleiotropy and InSIDE satisfied. The upper row corresponds to *θ* = 0 and the lower one to *θ* = 0.2; each column corresponds to *m* = 10, 30, 100 respectively.

**Fig 5:**
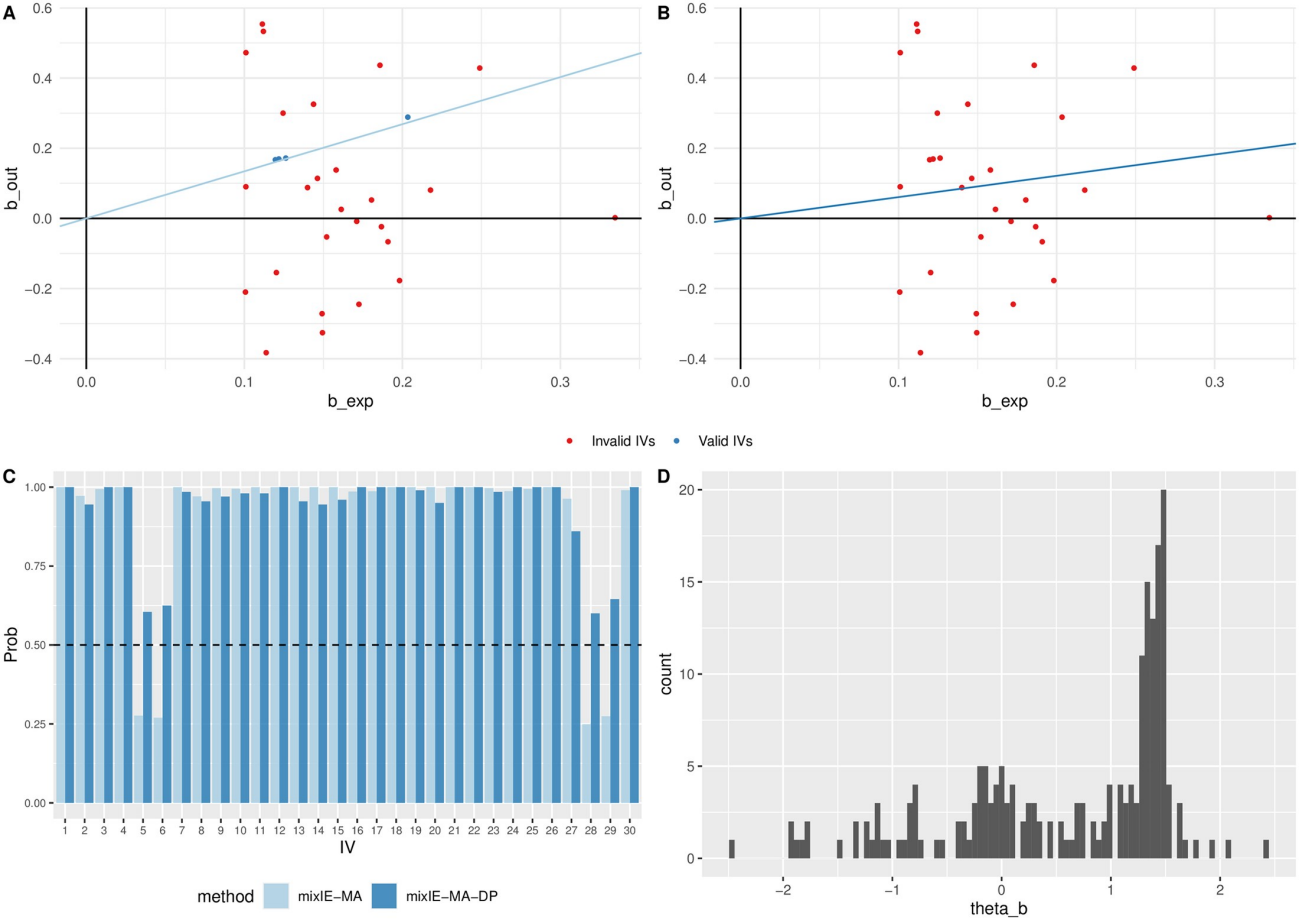
A simulated data example with *n* = 50 000, *m* = 30, *θ* = 0, p_invalid = 1 under directional pleiotropy and InSIDE satisfied. A: Causal estimate and identified invalid IVs by mixIE-MA. B: Causal estimate and identified invalid IVs by mixIE-MA-DP. C: Posterior probability of each IV being invalid. D: Histogram of ${\hat{\theta }}^{(b)*}$ from 200 perturbations.

Fig 4 shows the estimated proportions of invalid IVs by mixIE-MA in different scenarios. We can see that it was able to estimate the proportion of invalid IVs reasonably well, but under-estimated it when the proportion of invalid IVs was high. We argue that this was probably due to the weak identifiability of the mixture model: in such a scenario, the few points close to any line going through the origin point could be reasonably regarded as valid IVs. As shown in Fig 5A, although mixIE-MA identified most of the IVs to be invalid, the 4 (blue) points were identified to be valid IVs, driving a causal estimate of 1.34 with p-value < 0.05 and resulting in a type 1 error. While in Fig 5B, mixIE-MA-DP classified all IVs as invalid and gave a point estimate of 0.61 with p-value 0.54. In Fig 5C, using data perturbation, mixIE-MA-DP was able to identify more than 100 times out of 200 perturbations that those 4 IVs were invalid. As shown in the histogram of the causal estimates ${\hat{\theta }}^{(b)*}$ from 200 data perturbations in Fig 5D, there is a peak around the original estimate 1.34, a small peak around the true value 0, and some negative estimates. Again, from this example we can see that, first, data perturbation was able to account for some model selection uncertainties; second, when mixIE-MA estimated a high proportion of invalid IVs, the result might be unreliable.

#### InSIDE violated

**Fig 6:**
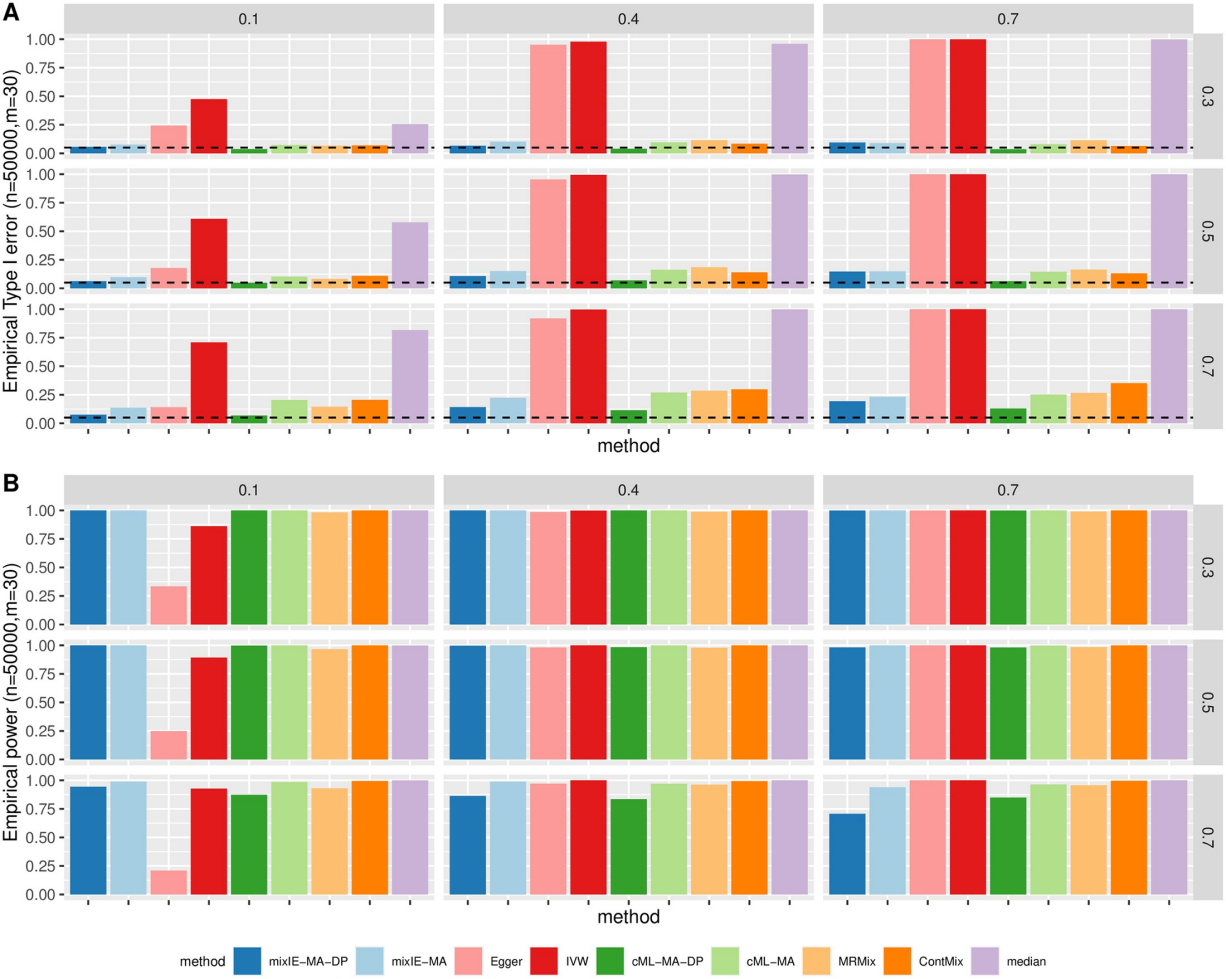
Simulation results with directional pleiotropy and InSIDE violated. A: Empirical type-I error; B: Power with sample size *n* = 50 000 and *m* = 30. Each column corresponds to *b* = 0.1, 0.4, 0.7 and each row corresponds to 30%, 50%, 70% invalid IVs.

**Fig 7:**
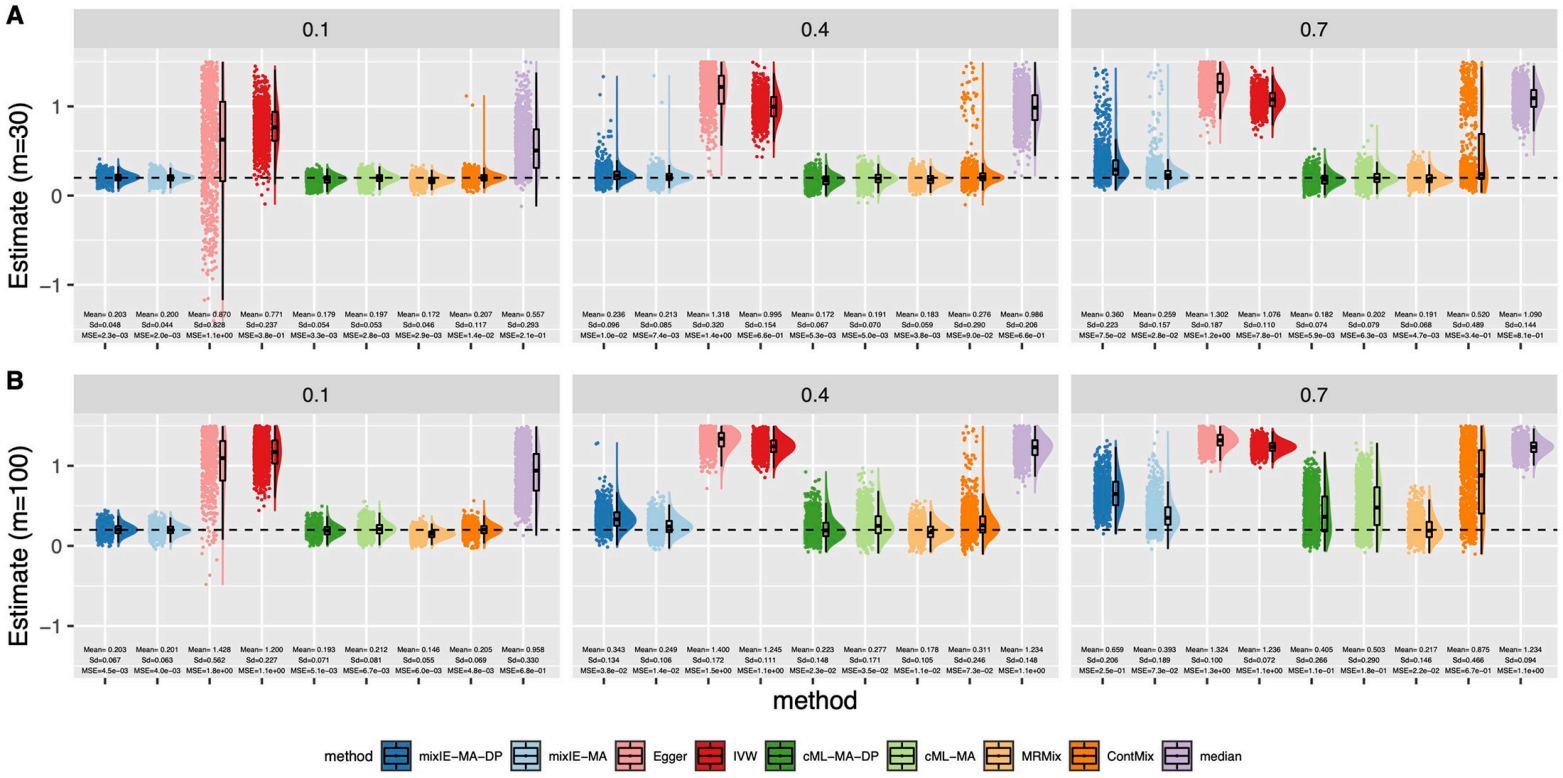
Simulation results with directional pleiotropy and InSIDE violated. Empirical distributions of the estimates of the causal effect *θ* by the methods with *n* = 50 000, *θ* = 0.2 and 70% invalid IVs. A: *m* = 30. B: *m* = 100. Each column corresponds to *b* = 0.1, 0.4, 0.7.

Fig 6 shows the empirical type 1 error and power of different methods in the presence of directional pleiotropy and with the violation of the InSIDE assumption to different degrees for *m* = 30 IVs. Other results are shown in Section B.1.3 in S1 Text. First, when the correlated pleiotropy (*ϕ*_*ug*_) was relatively small compared to the directional pleiotropy (e.g. when *b* = 0.1), our proposed method mixIE-MA-DP was still able to control the type 1 error and achieve high power. Also as shown in Fig 7, mixIE gave unbiased estimates when *b* = 0.1. In contrast, Egger regression yielded inflated type 1 error and highly biased estimates, so did IVW and weighted-median. Other methods such as MRMix and MR-ContMix also had inflated type 1 error when the proportion of invalid IVs was high. However, as the degree of InSIDE assumption violation increased (i.e., the effect size of correlated pleiotropy increased), as expected, the performance of our proposed method went down as with larger inflated type 1 error and more biased estimates. On the other hand, it still performed more robustly than Egger regression, which almost completely broke down. We also point out that, unlike when the InSIDE assumption held as shown before, the data perturbation version mixIE-MA-DP performed worse than mixIE-MA when the effect size of correlated pleiotropy was large. This is probably due to the fact that the proposed data perturbation scheme depends on the InSIDE assumption. In terms of estimation and inference, cML-MA-DP performed most robustly among all methods when the proportion of invalid IVs was small (e.g. 30%) regardless of the degrees of the violation of the InSIDE assumption—it could control the type 1 error well while yielding unbiased estimates. In terms of estimation, MRMix performed robustly as well—it could give (almost) unbiased estimates in most of the scenarios.

We conclude that when the InSIDE assumption is violated moderately and/or the proportion of invalid IVs is small, our proposed methods still have an edge over most of the popular MR methods, especially compared with Egger regression and IVW. As the degree of the violation increases, it still performs more robustly than Egger regression, IVW and weighted-median.

In summary, with the motivation of balancing and combining IVW and Egger regression, in most of the scenarios, our proposed methods were able to boost statistical power dramatically over Egger regression while controlling the type 1 error satisfactorily. The proposed methods were also able to control the type 1 error much better than IVW (except when all IVs were invalid with balanced pleiotropy and InSIDE satisfied) and improved the power in many scenarios. In general, as we mentioned before, under the InSIDE assumption, when mixIE-MA gives a high estimated proportion of invalid IVs (e.g. > 70%), we suggest that mixIE-MA-DP could give more reliable results and we could even go with Egger regression when (almost) all IVs are invalid, which might be conservative; on the other hand, when mixIE-MA gives a small estimated proportion of invalid IVs (e.g. < 20%), the original method and its data perturbation version mixIE-MA-DP are expected to give good and similar results.

#### Model checking

**Fig 8:**
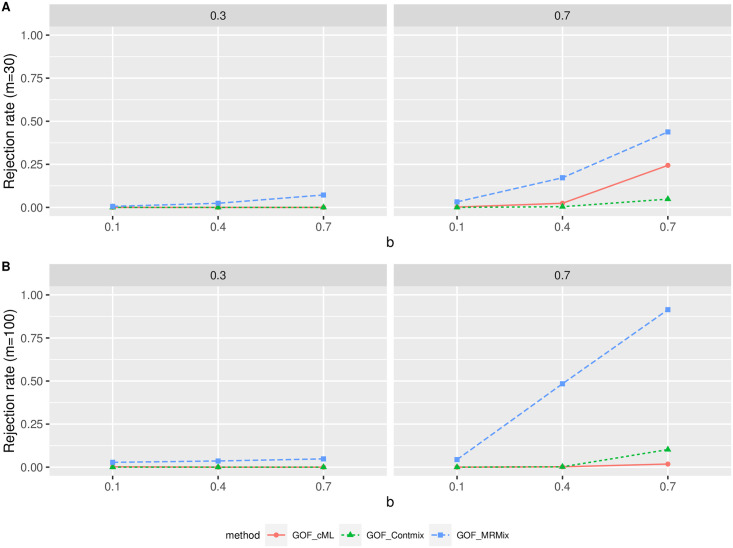
Simulation results for GOF testing with directional pleiotropy and InSIDE violated (while the plurality assumption for other three methods holding). *θ* = 0.2 and *n* = 50 000. The y-axis gives the rejection rate that the results from two methods were consistent, while the x-axis gives the increasing degree of InSIDE being violated. A: *m* = 30. B: *m* = 100. The two columns correspond to 30% and 70% invalid IVs respectively.

We applied the proposed GOF testing procedure under the main simulation scenario (c) with directional pleiotropy and InSIDE violated, under which the plurality of valid IV assumption held. We used *n* = 50 000 and *θ* = 0.2, and varying proportions of invalid IVs and degrees of violation of the InSIDE assumption. We compared mixIE-MA with three other methods, cML-MA, MRMix and MR-ContMix, and the corresponding tests are referred as GOF-cML, GOF-MRMix and GOF-ContMix respectively. Fig 8 shows the rejection rates of the three tests for 30 or 100 IVs. First, when the degree of violation of InSIDE assumption was small (*b* = 0.1) and/or the proportion of invalid IVs was small (30%), mixIE and cML, MR-ContMix had good consistency, while there was some inconsistency between mixIE and MRMix. This was perhaps because the estimates of MRMix were biased towards the null. Second, when the proportion of invalid IVs was high (70%), the rejection rate of GOF-MRMix increased as the degree of the InSIDE violation (i.e. *b*) increased, since the estimates of mixIE became more biased while MRMix still yielded stable estimates as shown in Fig 7. Meanwhile, GOF-cML and GOF-ContMix had low rejection rates when *m* = 100 because the other two methods both gave biased estimates as mixIE with the same bias direction and to a similar degree (Fig 7B). In short, the proposed GOF testing was able to capture inconsistency among different methods, which could be due to the violation of the InSIDE assumption if other assumptions of mixIE and the other method held. It also depended on the performance of the other method being compared with mixIE.

#### Simulations with weak invalid IVs

**Fig 9:**
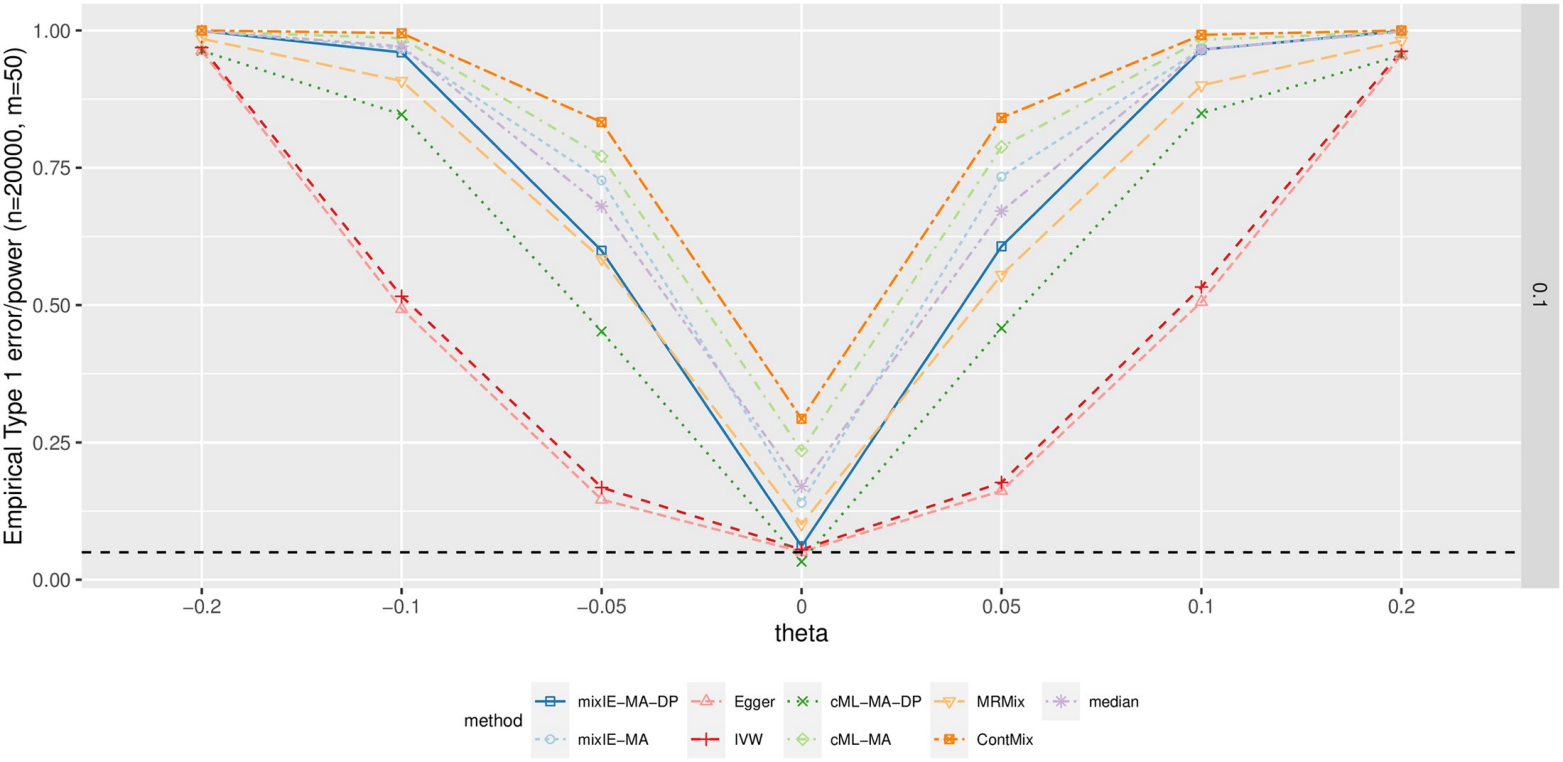
Simulation results with many invalid IVs having weak pleiotropic effects. Empirical type-I error (for *θ* = 0) and power (for *θ* ≠ 0) curves with sample size *n* = 20000 and *h*_*y*_ = 0.1.

Fig 9 shows the empirical type 1 error and power when *h*_*y*_ = 0.1 for different methods; the complete results are given in Section B.2 in S1 Text. In agreement with [14], cML-MA-DP, Egger regression, IVW and our proposed method mixIE-MA-DP could control the type 1 error across different scenarios, while MR-ContMix gave the highest type 1 error. mixIE-MA-DP was much more powerful than both Egger regression and IVW. It was also slightly more powerful than cML-MA-DP, especially with weaker direct effects of invalid IVs when *h*_*y*_ = 0.1, in which case it was more challenging for cML-MA-DP to identify invalid IVs. Also, as shown in Supplementary, mixIE-MA-DP was unbiased while cML-MA-DP had slight under-estimation biases towards the null. We also note that the power for Egger regression shown here was larger than the one shown in [14] because we did not re-orient SNPs for Egger regression for the purpose of fair comparison.

#### Computational time

We did simulations to compare the computational time for each method. The details are given in Section E in S1 Text. In summary, our proposed methods run reasonably fast. The computational time of mixIE-MA is comparable to that of cML-MA and faster than weighted-median and MRMix, but slower than IVW, Egger regression and MR-ContMix. As expected, with data perturbation it takes longer to run mixIE-MA-DP, though it is still quite feasible: with 200 perturbations and 50 starting points within each perturbation, it only took less than a minute with 10 to 100 IVs on a Macbook laptop.

### Primary real data example

#### Main analysis

We compared our proposed methods with other methods to identify causal effects of 12 risk factors for cardiometabolic diseases on coronary artery disease (CAD), stroke and type 2 diabetes (T2D), as well as asthma, which largely served as a negative control. The sample sizes for the traits are summarized in Table 1. Following [14], we classified the 48 pairs into 4 categories: 19 pairs considered causal or likely causal as supported by the literature, 17 pairs correlated but unknown to be causal or with conflicting evidence, 10 pairs unrelated, and 2 pairs considered non-causal.

**Table 1:** Genome wide association studies for 4 common diseases and 12 risk factors.

Abbreviation	Trait	Sample size	Number of variants used in MR^1^	Reference
TG	triglycerides	188577	122–128	[28]
LDL	low-density lipoproteins	188577	173–184	[28]
HDL	high-density lipoproteins	188577	188–197	[28]
Height	body height	253288	977–986	[29]
BMI	body mass index	322154	88–90	[30]
BF	body fat percentage	100716	9–10	[31]
BW	birth weight	153781	54–65	[32]
DBP	diastolic blood pressure	757601	1108–1345	[33]
SBP	systolic blood pressure	757601	1106–1324	[33]
FG	fasting glucose	46186	17	[34]
Smoke	ever regular smoker	1232091	114–129	[35]
Alcohol	drinks per week	941280	44–54	[35]
CAD	coronary artery disease	547261		[36]
Stroke	any stroke	446696		[37]
T2D	type 2 diabetes	69033		[38]
Asthma	asthma	142486		[39]

^1^ This is the range of the number of variants used in the analysis for the risk factor (exposure), which would be slightly different based on the disease (outcome). Specific numbers are given in Table AG in S1 Text.

**Fig 10:**
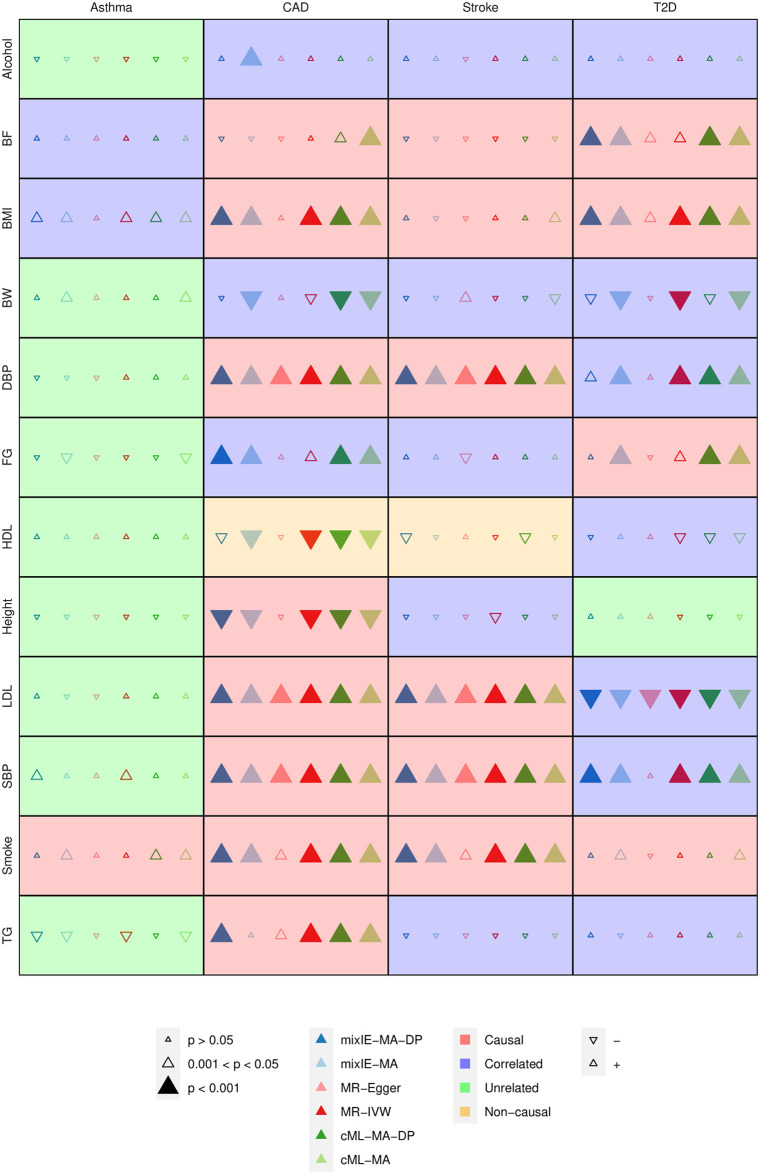
Results of various methods to detect causal relationships among 48 risk factor-disease pairs.

In the main analysis, we re-oriented SNPs such that all IVs were positively associated with the exposure as recommended for Egger regression [15]; the results when we did not re-orient the SNPs are shown in Section C.2.3 in S1 Text as a sensitivity analysis. In Fig 10 we compare mixIE-MA-DP and mixIE-MA with Egger regression, IVW, cML-MA-DP and cML-MA. Table 2 compares the total numbers of the pairs identified to be significant by different methods at the significance level 0.001 (with a Bonferroni adjustment 0.05/48 ≈ 0.001). First, mixIE-MA and mixIE-MA-DP identified more causal risk factor-disease pairs than Egger regression and IVW. Egger regression only identified 6 known or likely causal pairs, while our proposed methods identified 13 pairs. As an example, for the causal BF-T2D pair, both mixIE-MA and mixIE-MA-DP gave some significant results while both Egger regression and IVW yielded only marginally significant ones. Both mixIE-MA and cML-MA identified the same single invalid IV and gave similar estimates. In contrast, IVW would be affected by the invalid IV and thus gave a smaller estimate with an only marginally significant p-value. See Section C.1.1 in S1 Text for more details.

**Table 2:** Numbers of significant pairs among 48 risk factor-disease pairs at the significance cutoff of p-value < 0.001.

	Causal	Correlated	Unrelated	Non-causal
mixIE-MA	13	7	0	1
mixIE-MA-DP	13	3	0	0
Egger	6	1	0	0
IVW	12	4	0	1
cML-MA	15	6	0	1
cML-MA-DP	14	5	0	1
MR-ContMix	11	5	0	1
Weighted-Median	12	2	0	1

Second, as shown in Table AG in S1 Text, among the 48 risk factor-disease pairs, most of the pairs had less than 20% IVs identified to be invalid by mixIE-MA. In agreement with the simulations, mixIE-MA-DP and mixIE-MA gave similar results in general when the (estimated) proportion of invalid IVs was small. However, they might give different results for some of the pairs with a high proportion of invalid IVs. For example, for (causal) FG-T2D pair, mixIE-MA identified about 40% of 17 SNPs to be invalid IVs and a significant causal effect (p-value < 0.001), while mixIE-MA-DP did not give a significant result. Another example is for the (causal) TG-CAD pair, mixIE-MA identified about 50% of 128 SNPs to be invalid IVs and had a p-value > 0.05, but mixIE-MA-DP gave a p-value < 0.001. See Section C.1.2 in S1 Text for more details.

Third, it is notable that mixIE-MA-DP was one of the only two methods that did not give a false positive for the HDL-CAD pair. The other one was Egger regression, which however is in general low-powered.

For model checking, due to the heavy computation burden of GOF-MRMix, we only applied GOF-cML and GOF-ContMix to compare our proposed mixIE with cML and MR-ContMix. It turned out that, at the 95% confidence level, there was only one pair, SBP-CAD, for which the (causal) estimate of mixIE was inconsistent with those from cML and MR-ContMix, though the three estimates were 0.031, 0.037 and 0.039 with only small differences, and all three were statistically significant (with 1324 SNPs/IVs). Nevertheless, cautions should be taken in interpreting the results for this pair.

Some sensitivity analysis results are included in Section C.2 in S1 Text.

### Secondary real data example

**Fig 11:**
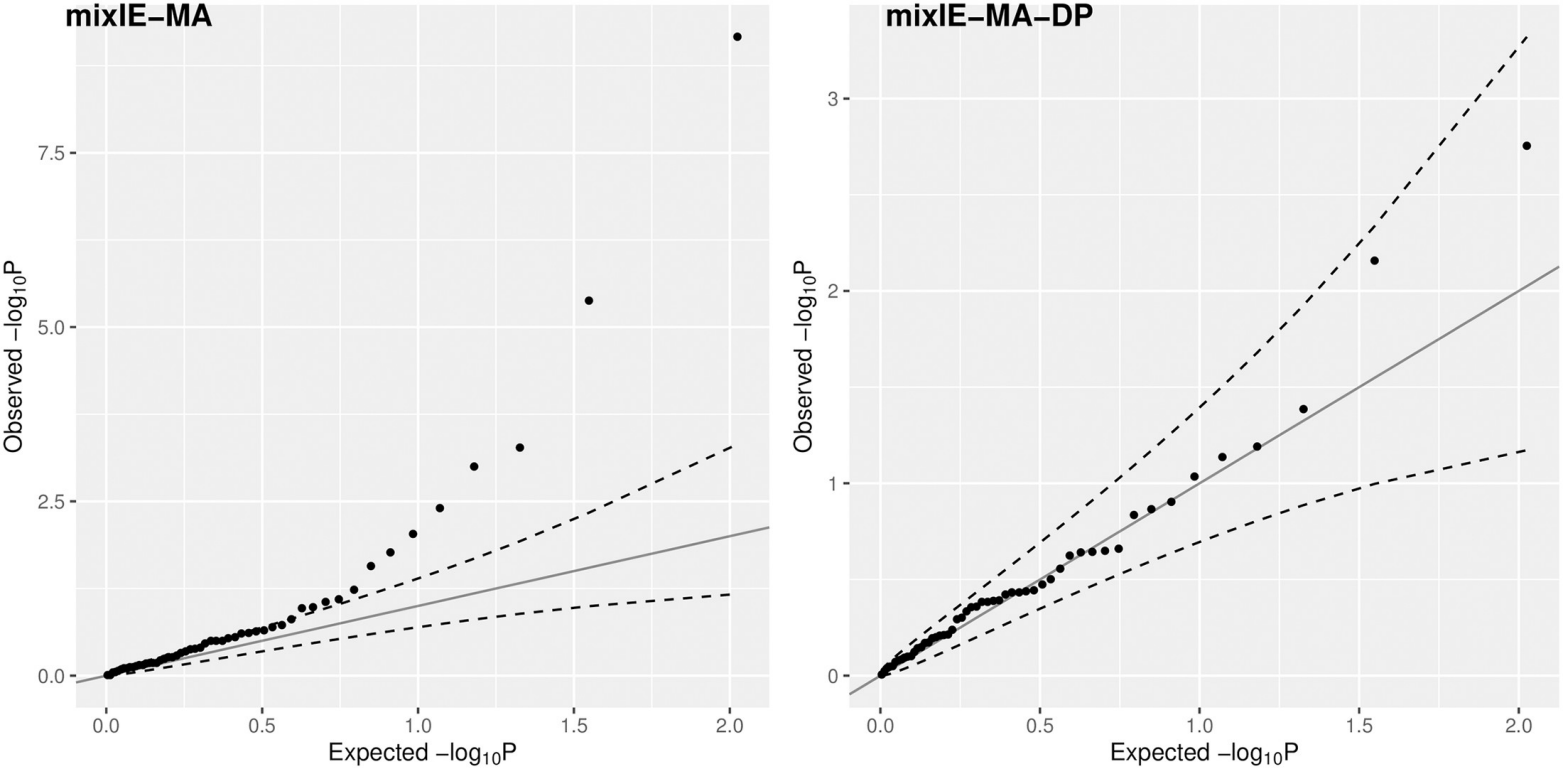
Q-Q plots for 53 (likely) null trait-pairs in the secondary real data examples. Left panel: mixIE-MA; right panel: mixIE-MA-DP.

Now we consider an example of 63 trait pairs that are not genetically correlated and thus are unlikely to be causally related. It can serve as a negative control to examine whether an MR method can control type I error satisfactorily. The main challenge with this example arises due to that some invalid IVs with only weak pleiotropic/direct effects are difficult to identify. The trait pairs include 13 traits: fasting proinsulin (FP), height, homeostasis model assessment of beta-cell function (HOMA), LDL, rheumatoid arthritis (RA), schizophrenia (SCZ), T2D, age at smoking, anorexia nervosa, childhood IQ, ever/never smoked, former current smoker, and infant head circumference. We applied the mixIE methods to these 63 genetically uncorrelated trait pairs. Following [14], we show the Q-Q plots of our proposed methods for the 53 pairs, which excluded 10 pairs with only 2 IVs. As shown in Fig 11, consistent with our simulation results, while mixIE-MA seemed to have inflated type I error, its data perturbation version mixIE-MA-DP appeared to be able to control the type I error well. The detailed results are given in Table AI in S1 Text. In contrast, as shown in the Supplementary of [14], MRMix and MR-ContMix yielded inflated type I errors too.

## Discussion

We have proposed mixIE, a new method to combine two of the most popular MR approaches, namely IVW and Egger regression, aiming to maintain each one’s strengths while overcoming their main limitations. We have found that model averaging performed better than the usual asymptotics-based statistical inference for finite samples, and thus proposed a model averaging approach as the default to implement mixIE, denoted mixIE-MA. We have also proposed a data perturbation-based version, mixIE-MA-DP, which can be more robust in accounting for model selection uncertainties, especially in more challenging situations (e.g. with many invalid IVs of small effects) for model selection with small sample sizes. We note that our proposed data perturbation scheme is novel in that it is applicable to GWAS summary data while dealing with the presence of both valid and invalid IVs at the same time. As shown in simulations and real data analyses, mixIE improved the power of Egger regression while controlling type I error rates well in most of the scenarios. It can handle directional pleiotropic effects by identifying invalid IVs while IVW cannot (with severely inflated type I error rates). Even in cases with balanced pleiotropy, mixIE could be often much more powerful than IVW (with a random-effect model). It also had some edge over its strong competitor cML in some scenarios with many weak invalid IVs. We have further demonstrated the usefulness of data perturbation through simulations and real data examples where it could better identify invalid IVs in some challenging scenarios. We also proposed a model checking procedure to compare our method with other methods, which do not require the InSIDE assumption, by evaluating the consistency of their estimates.

There are a few limitations of our proposed mixIE. First, like Egger regression, strictly speaking, our proposed method requires the InSIDE assumption to hold, and thus can be problematic with correlated pleiotropy (when the InSIDE assumption is violated). However, as shown in our simulations, even when the InSIDE assumption did not hold, mixIE still could often control type 1 error relatively close to a nominal level with decent power unless the degree of violation is dramatic; the reason is due to its (often much larger) dependence on the IVW estimate with valid IVs (than that on the Egger regression estimate). Relatedly, it is unclear yet how commonly correlated pleiotropy is present and what its typical effect size would be in real data. Although our proposed goodness-of-fit testing is able to detect inconsistency between the results from mixIE and another method, suggesting possible violation of the InSIDE assumption, it can be due to other reasons (i.e. other assumptions being violated). Second, inherited from Egger regression, mixIE may be sensitive to the coding/orientation of IVs. But as shown in the real data application, our proposed method was more robust than Egger regression, again due to its dependence on the IVW estimate with (detected) valid IVs. Until a better approach is available, we would follow the same guideline to SNP reorientation for Egger regression. Third, as a mixture model, mixIE may suffer from the weak identifiability issue, especially when the proportion of invalid IVs is close to 1. We alleviate this problem by a model averaging approach with both IVW and Egger regression models added in the list of the candidate models, as well as by data perturbation to better identify the set of (weak) invalid IVs. Fourth, as in typical MR, we assume and choose SNPs to be independent throughout this article. Extensions to the use of correlated SNPs may gain power in other applications, including transcriptome-wide association studies [40–44]. Furthermore, like most of summary-data based MR, we select and apply the instrument variables from and to the same exposure GWAS dataset, which could lead to biased inference because of selection bias [45]. Finally, more applications to real data, including comparisons with other MR methods, are warranted [3, 14, 27].

## Supporting information

S1 Text Supplementary file describing standard error estimation, additional simulation results, additional real data analysis results and computational time. (PDF)
