# Supplementary material for: Combining the strengths of inverse-variance weighting and Egger regression in Mendelian randomization using a mixture of regressions model
Source: PLoS Genet. 2021 Nov 18;17(11):e1009922. doi: 10.1371/journal.pgen.1009922 (PMC8639093; doi:10.1371/journal.pgen.1009922)
Supplement: S1 Text — (PDF) [file pgen.1009922.s001.pdf]

Supplementary materials: Combining the strengths of inverse-variance weighting and Egger regression in Mendelian randomization using a mixture of regressions model

A Standard errors of the CEM estimates

To obtain the standard errors of the estimated parameters, we take the approach proposed by [1]. Let $\boldsymbol{\alpha} = (\theta, r, c, \pi)$ be the set of all unknown parameters, $\mathbf{D}_i = (\hat{\beta}_{Xi}, \hat{\beta}_{Yi}, \hat{\sigma}^2_{Yi})$ be the observed GWAS summary data, $z_i = 1$ if SNP i is invalid, $z_i = 0$ otherwise. The complete-data log-likelihood is:

$$l_c(\boldsymbol{\alpha}; \mathbf{D}, \mathbf{z}) = \sum_{i=1}^{m} [z_i l_{1i}(\boldsymbol{\alpha}; \mathbf{D}_i) + z_i \log\pi + (1 - z_i) l_{0i}(\boldsymbol{\alpha}; \mathbf{D}_i) + (1 - z_i)\log(1 - \pi)],$$

where $l_{0i}(\boldsymbol{\alpha}; \mathbf{D}_i) = -\frac{1}{2}\left[\frac{(\hat{\beta}_{Yi} - \theta\hat{\beta}_{Xi})^2}{\hat{\sigma}^2_{Yi}} + \log(\hat{\sigma}^2_{Yi})\right], l_{1i}(\boldsymbol{\alpha}; \mathbf{D}_i) = -\frac{1}{2}\left[\frac{(\hat{\beta}_{Yi} - \theta\hat{\beta}_{Xi} - r)^2}{c\hat{\sigma}^2_{Yi}} + \log(c\hat{\sigma}^2_{Yi})\right].$
Following the similar notations in the original paper, we let

$$\boldsymbol{S}_c(\boldsymbol{\alpha}; \mathbf{D}, \mathbf{z}) = \partial l_c(\boldsymbol{\alpha}; \mathbf{D}, \mathbf{z})/\partial\boldsymbol{\alpha},$$

$$\boldsymbol{B}_c(\boldsymbol{\alpha}; \mathbf{D}, \mathbf{z}) = -\partial^2 l_c(\boldsymbol{\alpha}; \mathbf{D}, \mathbf{z})/\partial\boldsymbol{\alpha}\partial\boldsymbol{\alpha}^T.$$

be the gradient vector of the *complete*-data log-likelihood and the matrix of the negative of the second-order partial derivatives of *complete*-data log-likelihood respectively. And similarly, we denote the corresponding derivatives for *incomplete*-data (or *observed* data) as $\boldsymbol{S}_{obs}(\boldsymbol{\alpha}; \mathbf{D})$ and $\boldsymbol{B}_{obs}(\boldsymbol{\alpha}; \mathbf{D})$. From (3.2) in [1], we have

$$\boldsymbol{B}_{obs}(\boldsymbol{\alpha}; \mathbf{D}) = \mathbb{E}_{\boldsymbol{\alpha}}[\boldsymbol{B}_c|\mathbf{D}] - \mathbb{E}_{\boldsymbol{\alpha}}[\boldsymbol{S}_c\boldsymbol{S}_c^T|\mathbf{D}] + \boldsymbol{S}_{obs}(\boldsymbol{\alpha}; \mathbf{D})\boldsymbol{S}^T_{obs}(\boldsymbol{\alpha}; \mathbf{D}). \quad (1)$$

Since $\boldsymbol{S}_{obs}(\hat{\boldsymbol{\alpha}}; \mathbf{D}) = 0$, the last term of Equation (1) disappears and the observed information matrix can be computed as

$$\boldsymbol{B}_{obs}(\hat{\boldsymbol{\alpha}}; \mathbf{D}) = [\mathbb{E}_{\boldsymbol{\alpha}}[\boldsymbol{B}_c|\mathbf{D}]]_{\boldsymbol{\alpha}=\hat{\boldsymbol{\alpha}}} - \left[\mathbb{E}_{\boldsymbol{\alpha}}[\boldsymbol{S}_c\boldsymbol{S}_c^T|\mathbf{D}]\right]_{\boldsymbol{\alpha}=\hat{\boldsymbol{\alpha}}}. \quad (2)$$

In our case, we have

$$\begin{aligned}
\boldsymbol{S}_c &= \left[\frac{\partial l_c}{\partial \theta}, \frac{\partial l_c}{\partial r}, \frac{\partial l_c}{\partial c}, \frac{\partial l_c}{\partial \pi}\right]^T, \\
\frac{\partial l_c}{\partial \theta} &= \sum_{i=1}^m \left[z_i \frac{(\hat{\beta}_{Yi} - \theta\hat{\beta}_{Xi} - r)\hat{\beta}_{Xi}}{c\hat{\sigma}_{Yi}^2} + (1-z_i)\frac{(\hat{\beta}_{Yi} - \theta\hat{\beta}_{Xi})\hat{\beta}_{Xi}}{\hat{\sigma}_{Yi}^2} \right], \\
\frac{\partial l_c}{\partial r} &= \sum_{i=1}^m z_i \frac{(\hat{\beta}_{Yi} - \theta\hat{\beta}_{Xi} - r)}{c\hat{\sigma}_{Yi}^2}, \\
\frac{\partial l_c}{\partial c} &= -\frac{1}{2}\sum_{i=1}^m z_i \left[-\frac{(\hat{\beta}_{Yi} - \theta\hat{\beta}_{Xi} - r)^2}{c^2\hat{\sigma}_{Yi}^2} + \frac{1}{c} \right], \\
\frac{\partial l_c}{\partial \pi} &= \sum_{i=1}^m \left(\frac{z_i}{\pi} - \frac{1-z_i}{1-\pi} \right).
\end{aligned}$$

And we take the expectation of $\mathbf{z}$ given $\mathbf{D}$ and $\hat{\boldsymbol{\alpha}}$ by substituting z_i with $\hat{\tau}_{i,1}$ and plug in our estimates $\hat{\boldsymbol{\alpha}}$ to obtain $\left[\mathbb{E}_{\boldsymbol{\alpha}}[\boldsymbol{S}_c\boldsymbol{S}_c^T|\mathbf{D}]\right]_{\boldsymbol{\alpha}=\hat{\boldsymbol{\alpha}}}$.

For $\boldsymbol{B}_c$, we calculate the second derivative as follows:

$$\begin{aligned}
\frac{\partial^2(\text{-}l_c)}{\partial \theta^2} &= \sum_{i=1}^m \left[z_i \frac{\hat{\beta}_{Xi}^2}{c\hat{\sigma}_{Yi}^2} + (1-z_i)\frac{\hat{\beta}_{Xi}^2}{\hat{\sigma}_{Yi}^2} \right], \quad \frac{\partial^2(\text{-}l_c)}{\partial r^2} = \sum_{i=1}^m \frac{z_i}{c\hat{\sigma}_{Yi}^2}, \\
\frac{\partial^2(\text{-}l_c)}{\partial c^2} &= \sum_{i=1}^m z_i \left[\frac{(\hat{\beta}_{Yi} - \theta\hat{\beta}_{Xi} - r)^2}{c^3\hat{\sigma}_{Yi}^2} - \frac{1}{2c^2} \right], \quad \frac{\partial^2(\text{-}l_c)}{\partial \pi^2} = \sum_{i=1}^m \left(\frac{z_i}{\pi^2} - \frac{1-z_i}{(1-\pi)^2} \right), \\
\frac{\partial^2(\text{-}l_c)}{\partial \theta \partial r} &= \sum_{i=1}^m \frac{z_i\hat{\beta}_{Xi}}{c\hat{\sigma}_{Yi}^2}, \quad \frac{\partial^2(\text{-}l_c)}{\partial \theta \partial c} = \sum_{i=1}^m \frac{z_i(\hat{\beta}_{Yi} - \theta\hat{\beta}_{Xi} - r)\hat{\beta}_{Xi}}{c^2\hat{\sigma}_{Yi}^2}, \\
\frac{\partial^2(\text{-}l_c)}{\partial r \partial c} &= \sum_{i=1}^m \frac{z_i(\hat{\beta}_{Yi} - \theta\hat{\beta}_{Xi} - r)}{c^2\hat{\sigma}_{Yi}^2}.
\end{aligned}$$

And similarly, we take the expectation of $\mathbf{z}$ given $\mathbf{D}$ and $\hat{\boldsymbol{\alpha}}$ by substituting z_i with $\hat{\tau}_{i,1}$ and plug in $\hat{\boldsymbol{\alpha}}$ to obtain $[\mathbb{E}_{\boldsymbol{\alpha}}[\boldsymbol{B}_c|\mathbf{D}]]_{\boldsymbol{\alpha}=\hat{\boldsymbol{\alpha}}}$. The above idea is developed based on the traditional EM algorithm, however in CEM, we still follow the similar idea, except that we plug in $\hat{\pi} = \hat{K}/m$. Sometimes, by plugging in $\hat{\pi} = \hat{K}/m$ we might end up with a negative variance, then we use $\hat{\pi} = \sum_{i=1}^m \hat{\tau}_{i,1}$ as in the EM.

B Simulation

B.1 Main Simulation

B.1.1 Directional pleiotropy, InSIDE satisfied

Table A. In each cell, from top to bottom are empirical type-I error/power, mean($\hat{\theta}$), SD($\hat{\theta}$), mean(SE($\hat{\theta}$)), coverage rate, MSE, when $n = 10\,000$, p_invalid=0

m	θ	mixIE-MA	mixIE-MA-DP	cML-MA	cML-MA-DP	Egger	IVW	median	MRMix	ContMix
10	0.0	0.054<br>-0.001<br>0.036<br>0.035<br>0.946<br>0.001	0.051<br>-0.001<br>0.035<br>0.036<br>0.949<br>0.001	0.054<br>-0.001<br>0.035<br>0.035<br>0.946<br>0.001	0.032<br>-0.001<br>0.033<br>0.037<br>0.968<br>0.001	0.015<br>0.002<br>0.146<br>0.149<br>0.961<br>0.021	0.045<br>-0.001<br>0.035<br>0.037<br>0.955<br>0.001	0.031<br>-0.001<br>0.041<br>0.046<br>0.969<br>0.002	0.075<br>-0.001<br>0.063<br>0.067<br>0.925<br>0.004	0.058<br>-0.002<br>0.065<br>NA<br>0.900<br>0.004
	0.2	0.992<br>0.197<br>0.040<br>0.039<br>0.936<br>0.002	0.986<br>0.197<br>0.040<br>0.041<br>0.946<br>0.002	0.996<br>0.198<br>0.040<br>0.040<br>0.934<br>0.002	0.992<br>0.194<br>0.041<br>0.044<br>0.950<br>0.002	0.132<br>0.170<br>0.164<br>0.167<br>0.956<br>0.028	0.994<br>0.197<br>0.039<br>0.041<br>0.947<br>0.002	0.964<br>0.195<br>0.047<br>0.052<br>0.965<br>0.002	0.785<br>0.173<br>0.075<br>0.133<br>0.881<br>0.006	0.967<br>0.229<br>0.054<br>NA<br>0.882<br>0.004
30	0.0	0.050<br>-0.001<br>0.030<br>0.030<br>0.950<br>0.001	0.034<br>0.000<br>0.029<br>0.033<br>0.966<br>0.001	0.047<br>0.000<br>0.029<br>0.030<br>0.953<br>0.001	0.018<br>0.000<br>0.028<br>0.034<br>0.982<br>0.001	0.041<br>-0.002<br>0.097<br>0.098<br>0.946<br>0.009	0.044<br>0.000<br>0.028<br>0.030<br>0.956<br>0.001	0.027<br>0.000<br>0.037<br>0.042<br>0.973<br>0.001	0.030<br>-0.004<br>0.052<br>0.086<br>0.970<br>0.003	0.072<br>-0.001<br>0.056<br>NA<br>0.928<br>0.003
	0.2	0.995<br>0.193<br>0.034<br>0.034<br>0.941<br>0.001	0.998<br>0.194<br>0.033<br>0.038<br>0.960<br>0.001	1.000<br>0.200<br>0.033<br>0.034<br>0.946<br>0.001	1.000<br>0.195<br>0.033<br>0.040<br>0.972<br>0.001	0.259<br>0.143<br>0.112<br>0.110<br>0.919<br>0.016	1.000<br>0.195<br>0.032<br>0.034<br>0.951<br>0.001	0.984<br>0.191<br>0.042<br>0.048<br>0.962<br>0.002	0.726<br>0.174<br>0.074<br>0.127<br>0.934<br>0.006	0.991<br>0.235<br>0.054<br>NA<br>0.916<br>0.004
100	0.0	0.081<br>0.000<br>0.045<br>0.030<br>0.919<br>0.002	0.046<br>0.000<br>0.033<br>0.040<br>0.954<br>0.001	0.072<br>0.000<br>0.036<br>0.032<br>0.928<br>0.001	0.019<br>0.000<br>0.035<br>0.042<br>0.981<br>0.001	0.033<br>0.000<br>0.073<br>0.076<br>0.964<br>0.005	0.057<br>0.000<br>0.032<br>0.031<br>0.943<br>0.001	0.035<br>-0.001<br>0.041<br>0.046<br>0.965<br>0.002	0.029<br>0.004<br>0.067<br>0.574<br>0.971<br>0.005	0.069<br>0.001<br>0.043<br>NA<br>0.911<br>0.002
	0.2	0.993<br>0.181<br>0.048<br>0.035<br>0.872<br>0.003	0.892<br>0.181<br>0.038<br>0.049<br>0.925<br>0.002	0.999<br>0.199<br>0.041<br>0.037<br>0.930<br>0.002	0.995<br>0.193<br>0.040<br>0.048<br>0.980<br>0.002	0.151<br>0.083<br>0.082<br>0.085<br>0.710<br>0.020	0.998<br>0.180<br>0.036<br>0.035<br>0.891<br>0.002	0.943<br>0.170<br>0.045<br>0.052<br>0.937<br>0.003	0.672<br>0.180<br>0.080<br>0.172<br>0.938<br>0.007	0.988<br>0.186<br>0.048<br>NA<br>0.861<br>0.002

Table B. In each cell, from top to bottom are empirical type-I error/power, mean($\hat{\theta}$), SD($\hat{\theta}$), mean(SE($\hat{\theta}$)), coverage rate, MSE, when $n = 10\,000$, p_invalid=0.3

m	θ	mixIE-MA	mixIE-MA-DP	cML-MA	cML-MA-DP	Egger	IVW	median	MRMix	ContMix
10	0.0	0.086	0.055	0.073	0.031	0.041	0.069	0.100	0.071	0.069
		0.004	0.011	0.002	0.002	-0.013	0.119	0.018	0.000	0.001
		0.111	0.188	0.047	0.043	0.949	0.208	0.071	0.062	0.056
		0.062	0.124	0.043	0.046	0.782	0.196	0.055	0.081	NA
		0.914	0.945	0.927	0.969	0.924	0.931	0.900	0.929	0.915
		0.012	0.036	0.002	0.002	0.900	0.057	0.005	0.004	0.003
	0.2	0.854	0.661	0.967	0.870	0.049	0.443	0.917	0.827	0.933
		0.200	0.206	0.202	0.191	0.156	0.318	0.217	0.183	0.207
		0.145	0.203	0.057	0.057	0.950	0.208	0.079	0.065	0.069
		0.074	0.147	0.048	0.058	0.786	0.197	0.062	0.064	NA
		0.903	0.943	0.913	0.954	0.926	0.940	0.906	0.919	0.880
		0.021	0.041	0.003	0.003	0.903	0.057	0.006	0.004	0.005
30	0.0	0.085	0.054	0.089	0.024	0.066	0.169	0.087	0.036	0.074
		0.001	0.002	0.001	0.002	-0.003	0.177	0.023	-0.001	0.001
		0.043	0.044	0.043	0.040	0.622	0.193	0.059	0.055	0.045
		0.038	0.044	0.037	0.045	0.582	0.180	0.052	0.066	NA
		0.915	0.946	0.911	0.976	0.927	0.831	0.913	0.964	0.923
		0.002	0.002	0.002	0.002	0.387	0.069	0.004	0.003	0.002
	0.2	0.988	0.970	0.992	0.950	0.067	0.561	0.966	0.812	0.983
		0.196	0.197	0.203	0.194	0.144	0.372	0.217	0.180	0.196
		0.049	0.050	0.051	0.049	0.622	0.193	0.064	0.058	0.050
		0.042	0.050	0.042	0.054	0.584	0.181	0.058	0.066	NA
		0.900	0.939	0.892	0.962	0.923	0.845	0.938	0.931	0.926
		0.002	0.002	0.003	0.002	0.389	0.067	0.004	0.004	0.003
100	0.0	0.073	0.053	0.114	0.020	0.057	0.409	0.113	0.032	0.082
		-0.001	0.001	0.004	0.004	0.025	0.337	0.042	0.004	0.000
		0.051	0.052	0.057	0.053	0.489	0.192	0.066	0.064	0.052
		0.047	0.051	0.045	0.064	0.473	0.193	0.063	0.080	NA
		0.927	0.947	0.886	0.980	0.941	0.591	0.887	0.968	0.914
		0.003	0.003	0.003	0.003	0.239	0.150	0.006	0.004	0.003
	0.2	0.921	0.892	0.966	0.823	0.065	0.778	0.875	0.572	0.941
		0.181	0.181	0.211	0.202	0.108	0.517	0.217	0.166	0.180
		0.056	0.058	0.065	0.062	0.488	0.192	0.073	0.063	0.058
		0.051	0.057	0.050	0.073	0.474	0.194	0.070	0.082	NA
		0.898	0.926	0.874	0.974	0.940	0.634	0.938	0.952	0.909
		0.004	0.004	0.004	0.004	0.247	0.137	0.006	0.005	0.004

Table C. In each cell, from top to bottom are empirical type-I error/power, mean($\hat{\theta}$), SD($\hat{\theta}$), mean(SE($\hat{\theta}$)), coverage rate, MSE, when $n = 10\,000$, p_invalid=0.5

m	θ	mixIE-MA	mixIE-MA-DP	cML-MA	cML-MA-DP	Egger	IVW	median	MRMix	ContMix
10	0.0	0.077	0.039	0.113	0.046	0.061	0.131	0.250	0.102	0.111
		0.047	0.069	0.007	0.007	-0.003	0.201	0.065	0.007	0.016
		0.359	0.457	0.061	0.054	1.175	0.269	0.162	0.075	0.159
		0.140	0.287	0.050	0.056	1.028	0.258	0.066	0.073	NA
		0.923	0.961	0.887	0.954	0.901	0.869	0.750	0.898	0.889
		0.131	0.213	0.004	0.003	1.380	0.113	0.031	0.006	0.026
	0.2	0.658	0.388	0.885	0.692	0.072	0.381	0.886	0.732	0.881
		0.237	0.249	0.204	0.185	0.166	0.400	0.266	0.187	0.224
		0.388	0.459	0.076	0.073	1.174	0.268	0.163	0.081	0.188
		0.154	0.313	0.056	0.072	1.029	0.258	0.074	0.075	NA
		0.905	0.957	0.852	0.920	0.901	0.873	0.768	0.892	0.876
		0.152	0.213	0.006	0.006	1.379	0.112	0.031	0.007	0.036
30	0.0	0.113	0.052	0.138	0.025	0.044	0.239	0.200	0.061	0.121
		0.005	0.009	0.005	0.005	0.009	0.291	0.058	0.005	0.003
		0.060	0.061	0.059	0.051	0.783	0.241	0.105	0.065	0.059
		0.049	0.063	0.045	0.057	0.747	0.230	0.063	0.071	NA
		0.887	0.948	0.862	0.975	0.945	0.761	0.800	0.939	0.919
		0.004	0.004	0.003	0.003	0.612	0.143	0.014	0.004	0.003
	0.2	0.924	0.840	0.950	0.763	0.047	0.556	0.926	0.723	0.928
		0.200	0.205	0.207	0.191	0.156	0.486	0.257	0.178	0.198
		0.067	0.068	0.069	0.066	0.781	0.241	0.111	0.067	0.068
		0.054	0.071	0.050	0.071	0.747	0.231	0.071	0.073	NA
		0.874	0.942	0.842	0.941	0.947	0.764	0.813	0.904	0.910
		0.004	0.005	0.005	0.004	0.611	0.140	0.015	0.005	0.005
100	0.0	0.111	0.046	0.216	0.025	0.051	0.602	0.282	0.037	0.140
		0.002	0.005	0.017	0.018	0.001	0.549	0.106	0.005	0.010
		0.072	0.074	0.088	0.077	0.610	0.245	0.104	0.074	0.074
		0.061	0.078	0.057	0.092	0.596	0.245	0.080	0.087	NA
		0.889	0.954	0.784	0.975	0.944	0.398	0.718	0.963	0.898
		0.005	0.005	0.008	0.006	0.371	0.361	0.022	0.005	0.006
	0.2	0.749	0.591	0.881	0.566	0.064	0.854	0.871	0.452	0.829
		0.182	0.186	0.237	0.223	0.084	0.729	0.286	0.156	0.191
		0.079	0.081	0.104	0.091	0.610	0.245	0.113	0.076	0.082
		0.067	0.085	0.063	0.105	0.597	0.246	0.087	0.088	NA
		0.873	0.950	0.749	0.966	0.941	0.419	0.796	0.913	0.894
		0.007	0.007	0.012	0.009	0.385	0.340	0.020	0.008	0.007

Table D. In each cell, from top to bottom are empirical type-I error/power, mean($\hat{\theta}$), SD($\hat{\theta}$), mean(SE($\hat{\theta}$)), coverage rate, MSE, when $n = 10\,000$, p_invalid=0.7

m	θ	mixIE-MA	mixIE-MA-DP	cML-MA	cML-MA-DP	Egger	IVW	median	MRMix	ContMix
10	0.0	0.083	0.056	0.206	0.080	0.062	0.182	0.476	0.240	0.272
		0.099	0.130	0.015	0.011	0.040	0.285	0.164	0.035	0.104
		0.633	0.809	0.111	0.083	1.424	0.317	0.298	0.209	0.413
		0.384	0.684	0.062	0.072	1.225	0.306	0.078	0.082	NA
		0.917	0.944	0.794	0.920	0.905	0.818	0.524	0.760	0.837
		0.410	0.671	0.013	0.007	2.028	0.181	0.116	0.045	0.181
	0.2	0.308	0.151	0.724	0.477	0.061	0.381	0.838	0.675	0.796
		0.269	0.298	0.203	0.174	0.210	0.484	0.364	0.205	0.344
		0.639	0.828	0.131	0.113	1.421	0.316	0.299	0.204	0.463
		0.398	0.710	0.070	0.093	1.225	0.306	0.087	0.087	NA
		0.908	0.943	0.729	0.853	0.908	0.829	0.551	0.766	0.837
		0.413	0.694	0.017	0.013	2.018	0.180	0.116	0.042	0.235
30	0.0	0.139	0.048	0.220	0.044	0.037	0.313	0.396	0.101	0.196
		0.014	0.050	0.010	0.012	-0.018	0.400	0.154	0.004	0.014
		0.119	0.156	0.094	0.076	0.879	0.281	0.209	0.106	0.143
		0.073	0.134	0.056	0.079	0.878	0.270	0.080	0.097	NA
		0.861	0.952	0.780	0.956	0.950	0.687	0.604	0.899	0.833
		0.014	0.027	0.009	0.006	0.772	0.239	0.067	0.011	0.021
	0.2	0.733	0.491	0.788	0.463	0.050	0.600	0.886	0.553	0.812
		0.214	0.248	0.214	0.185	0.131	0.596	0.353	0.177	0.220
		0.130	0.154	0.119	0.100	0.878	0.281	0.213	0.113	0.203
		0.082	0.145	0.062	0.098	0.878	0.270	0.089	0.095	NA
		0.850	0.942	0.709	0.921	0.952	0.698	0.631	0.879	0.801
		0.017	0.026	0.014	0.010	0.774	0.235	0.069	0.013	0.042
100	0.0	0.172	0.036	0.378	0.042	0.060	0.761	0.569	0.066	0.310
		0.002	0.007	0.078	0.058	0.028	0.771	0.268	0.007	0.029
		0.129	0.126	0.276	0.142	0.740	0.297	0.217	0.113	0.131
		0.087	0.140	0.078	0.154	0.694	0.285	0.108	0.118	NA
		0.828	0.964	0.622	0.958	0.936	0.239	0.431	0.934	0.864
		0.017	0.016	0.082	0.024	0.548	0.682	0.119	0.013	0.018
	0.2	0.527	0.224	0.766	0.318	0.063	0.901	0.906	0.279	0.723
		0.186	0.188	0.342	0.278	0.110	0.951	0.455	0.152	0.216
		0.142	0.138	0.323	0.176	0.739	0.297	0.226	0.115	0.142
		0.093	0.154	0.089	0.173	0.695	0.286	0.116	0.118	NA
		0.808	0.970	0.539	0.929	0.935	0.263	0.500	0.884	0.865
		0.020	0.019	0.124	0.037	0.554	0.652	0.116	0.015	0.020

Table E. In each cell, from top to bottom are empirical type-I error/power, mean($\hat{\theta}$), SD($\hat{\theta}$), mean(SE($\hat{\theta}$)), coverage rate, MSE, when $n = 10\,000$, p_invalid=1

m	θ	mixIE-MA	mixIE-MA-DP	cML-MA	cML-MA-DP	Egger	IVW	median	MRMix	ContMix
10	0.0	0.203	0.088	0.624	0.311	0.054	0.223	0.792	0.742	0.867
		0.143	0.120	0.150	0.107	0.012	0.399	0.381	0.188	0.558
		1.038	1.298	0.425	0.323	1.579	0.385	0.462	0.536	0.970
		0.837	1.227	0.090	0.144	1.436	0.362	0.092	0.129	NA
		0.797	0.912	0.376	0.689	0.911	0.777	0.208	0.258	0.208
		1.096	1.696	0.203	0.116	2.489	0.307	0.359	0.322	1.251
	0.2	0.235	0.094	0.654	0.354	0.055	0.393	0.873	0.781	0.890
		0.317	0.293	0.255	0.176	0.181	0.599	0.581	0.279	0.818
		1.075	1.297	0.489	0.373	1.575	0.384	0.463	0.520	0.947
		0.855	1.246	0.097	0.156	1.436	0.362	0.102	0.127	NA
		0.803	0.921	0.317	0.573	0.910	0.779	0.223	0.259	0.220
		1.168	1.689	0.242	0.140	2.480	0.307	0.359	0.276	1.279
30	0.0	0.645	0.216	0.722	0.247	0.051	0.441	0.834	0.721	0.915
		0.398	0.382	0.224	0.143	0.033	0.577	0.523	0.209	0.749
		0.705	0.576	0.548	0.352	1.023	0.336	0.419	0.586	1.085
		0.268	0.636	0.093	0.192	1.023	0.316	0.108	0.289	NA
		0.355	0.784	0.278	0.753	0.938	0.559	0.166	0.279	0.264
		0.654	0.477	0.350	0.144	1.047	0.446	0.449	0.387	1.738
	0.2	0.663	0.272	0.761	0.292	0.058	0.678	0.904	0.713	0.920
		0.580	0.546	0.339	0.218	0.180	0.773	0.713	0.253	1.006
		0.700	0.582	0.582	0.398	1.020	0.336	0.419	0.566	1.101
		0.287	0.657	0.096	0.202	1.023	0.317	0.118	0.292	NA
		0.374	0.797	0.222	0.686	0.945	0.562	0.184	0.297	0.280
		0.634	0.459	0.358	0.159	1.040	0.441	0.439	0.323	1.860
100	0.0	0.685	0.106	0.953	0.503	0.061	0.896	0.939	0.538	0.910
		0.556	0.559	2.103	1.082	0.004	1.099	0.973	0.147	1.107
		2.001	1.151	1.570	1.081	0.831	0.351	0.455	0.680	1.205
		0.398	1.412	0.231	0.523	0.811	0.336	0.163	0.519	NA
		0.315	0.894	0.047	0.497	0.939	0.104	0.061	0.462	0.166
		4.308	1.635	6.887	2.339	0.690	1.332	1.153	0.483	2.677
	0.2	0.648	0.114	0.968	0.604	0.064	0.963	0.976	0.512	0.929
		0.674	0.689	2.327	1.333	0.088	1.280	1.144	0.180	1.294
		2.011	1.145	1.476	1.072	0.831	0.351	0.454	0.650	1.202
		0.426	1.406	0.220	0.491	0.811	0.336	0.172	1.017	NA
		0.344	0.901	0.037	0.434	0.939	0.112	0.071	0.507	0.188
		4.266	1.548	6.701	2.433	0.703	1.289	1.098	0.423	2.639

Table F. In each cell, from top to bottom are empirical type-I error/power, mean($\hat{\theta}$), SD($\hat{\theta}$), mean(SE($\hat{\theta}$)), coverage rate, MSE, when $n = 50\,000$, p_invalid=0

m	θ	mixIE-MA	mixIE-MA-DP	cML-MA	cML-MA-DP	Egger	IVW	median	MRMix	ContMix
10	0.0	0.046<br>0.000<br>0.015<br>0.015<br>0.954<br>0.000	0.041<br>0.000<br>0.015<br>0.016<br>0.959<br>0.000	0.046<br>0.000<br>0.015<br>0.015<br>0.954<br>0.000	0.033<br>0.000<br>0.015<br>0.016<br>0.967<br>0.000	0.012<br>0.002<br>0.068<br>0.070<br>0.963<br>0.005	0.039<br>0.000<br>0.015<br>0.016<br>0.961<br>0.000	0.033<br>0.000<br>0.019<br>0.021<br>0.967<br>0.000	0.077<br>0.000<br>0.027<br>0.040<br>0.923<br>0.001	0.063<br>0.000<br>0.029<br>NA<br>0.887<br>0.001
	0.2	1.000<br>0.200<br>0.018<br>0.017<br>0.938<br>0.000	0.999<br>0.200<br>0.018<br>0.018<br>0.949<br>0.000	1.000<br>0.200<br>0.018<br>0.018<br>0.945<br>0.000	1.000<br>0.199<br>0.018<br>0.019<br>0.953<br>0.000	0.616<br>0.195<br>0.077<br>0.079<br>0.960<br>0.006	1.000<br>0.200<br>0.018<br>0.019<br>0.954<br>0.000	1.000<br>0.200<br>0.022<br>0.023<br>0.962<br>0.000	0.885<br>0.173<br>0.054<br>0.794<br>0.809<br>0.004	1.000<br>0.208<br>0.021<br>NA<br>0.552<br>0.001
30	0.0	0.051<br>0.000<br>0.013<br>0.013<br>0.949<br>0.000	0.037<br>0.000<br>0.013<br>0.014<br>0.963<br>0.000	0.049<br>0.000<br>0.013<br>0.013<br>0.951<br>0.000	0.023<br>0.000<br>0.013<br>0.014<br>0.977<br>0.000	0.037<br>-0.001<br>0.049<br>0.049<br>0.954<br>0.002	0.043<br>0.000<br>0.013<br>0.014<br>0.957<br>0.000	0.024<br>0.000<br>0.017<br>0.019<br>0.976<br>0.000	0.043<br>0.000<br>0.025<br>0.052<br>0.957<br>0.001	0.078<br>0.000<br>0.027<br>NA<br>0.895<br>0.001
	0.2	1.000<br>0.199<br>0.015<br>0.015<br>0.942<br>0.000	1.000<br>0.199<br>0.015<br>0.016<br>0.957<br>0.000	1.000<br>0.200<br>0.015<br>0.015<br>0.954<br>0.000	1.000<br>0.199<br>0.015<br>0.017<br>0.965<br>0.000	0.877<br>0.185<br>0.056<br>0.056<br>0.952<br>0.003	1.000<br>0.199<br>0.015<br>0.015<br>0.952<br>0.000	1.000<br>0.198<br>0.019<br>0.021<br>0.976<br>0.000	0.830<br>0.173<br>0.062<br>0.062<br>0.833<br>0.005	1.000<br>0.217<br>0.019<br>NA<br>0.883<br>0.001
100	0.0	0.047<br>0.000<br>0.014<br>0.014<br>0.953<br>0.000	0.047<br>0.000<br>0.014<br>0.015<br>0.953<br>0.000	0.044<br>0.000<br>0.014<br>0.014<br>0.956<br>0.000	0.018<br>0.000<br>0.014<br>0.016<br>0.982<br>0.000	0.046<br>0.000<br>0.047<br>0.047<br>0.953<br>0.002	0.043<br>0.000<br>0.014<br>0.014<br>0.957<br>0.000	0.025<br>0.000<br>0.018<br>0.021<br>0.975<br>0.000	0.021<br>-0.001<br>0.026<br>0.075<br>0.979<br>0.001	0.076<br>0.001<br>0.029<br>NA<br>0.869<br>0.001
	0.2	1.000<br>0.196<br>0.017<br>0.016<br>0.937<br>0.000	1.000<br>0.196<br>0.016<br>0.016<br>0.947<br>0.000	1.000<br>0.200<br>0.017<br>0.016<br>0.949<br>0.000	1.000<br>0.198<br>0.017<br>0.019<br>0.977<br>0.000	0.827<br>0.155<br>0.053<br>0.053<br>0.852<br>0.005	1.000<br>0.196<br>0.016<br>0.016<br>0.949<br>0.000	1.000<br>0.194<br>0.021<br>0.024<br>0.967<br>0.000	0.794<br>0.176<br>0.054<br>0.143<br>0.896<br>0.004	1.000<br>0.236<br>0.025<br>NA<br>0.752<br>0.002

Table G. In each cell, from top to bottom are empirical type-I error/power, mean($\hat{\theta}$), SD($\hat{\theta}$), mean(SE($\hat{\theta}$)), coverage rate, MSE, when $n = 50\,000$, p_invalid=0.3

m	θ	mixIE-MA	mixIE-MA-DP	cML-MA	cML-MA-DP	Egger	IVW	median	MRMix	ContMix
10	0.0	0.080<br>0.001<br>0.048<br>0.023<br>0.920<br>0.002	0.066<br>0.004<br>0.062<br>0.039<br>0.934<br>0.004	0.067<br>0.000<br>0.020<br>0.019<br>0.933<br>0.000	0.050<br>0.000<br>0.020<br>0.020<br>0.950<br>0.000	0.063<br>-0.019<br>1.028<br>0.832<br>0.900<br>1.057	0.062<br>0.127<br>0.205<br>0.196<br>0.938<br>0.058	0.111<br>0.009<br>0.049<br>0.025<br>0.889<br>0.002	0.067<br>-0.001<br>0.027<br>0.029<br>0.933<br>0.001	0.074<br>-0.001<br>0.022<br>NA<br>0.906<br>0.000
	0.2	0.977<br>0.201<br>0.052<br>0.028<br>0.919<br>0.003	0.918<br>0.201<br>0.079<br>0.046<br>0.931<br>0.006	1.000<br>0.199<br>0.024<br>0.021<br>0.916<br>0.001	0.997<br>0.196<br>0.025<br>0.025<br>0.947<br>0.001	0.066<br>0.176<br>1.024<br>0.832<br>0.899<br>1.049	0.476<br>0.327<br>0.205<br>0.197<br>0.941<br>0.058	0.997<br>0.210<br>0.051<br>0.029<br>0.892<br>0.003	0.987<br>0.182<br>0.027<br>0.027<br>0.878<br>0.001	1.000<br>0.200<br>0.025<br>NA<br>0.895<br>0.001
30	0.0	0.070<br>0.000<br>0.018<br>0.017<br>0.930<br>0.000	0.050<br>0.000<br>0.018<br>0.018<br>0.950<br>0.000	0.066<br>0.000<br>0.018<br>0.017<br>0.934<br>0.000	0.031<br>0.000<br>0.017<br>0.018<br>0.969<br>0.000	0.057<br>-0.007<br>0.672<br>0.640<br>0.930<br>0.451	0.158<br>0.185<br>0.181<br>0.177<br>0.842<br>0.067	0.079<br>0.010<br>0.025<br>0.023<br>0.921<br>0.001	0.034<br>0.000<br>0.026<br>0.031<br>0.966<br>0.001	0.069<br>0.000<br>0.018<br>NA<br>0.915<br>0.000
	0.2	1.000<br>0.199<br>0.020<br>0.018<br>0.933<br>0.000	1.000<br>0.199<br>0.021<br>0.020<br>0.945<br>0.000	1.000<br>0.200<br>0.020<br>0.019<br>0.928<br>0.000	1.000<br>0.198<br>0.020<br>0.022<br>0.962<br>0.000	0.064<br>0.180<br>0.670<br>0.639<br>0.931<br>0.449	0.616<br>0.384<br>0.180<br>0.177<br>0.845<br>0.066	1.000<br>0.210<br>0.028<br>0.026<br>0.932<br>0.001	0.981<br>0.180<br>0.026<br>0.034<br>0.914<br>0.001	1.000<br>0.199<br>0.021<br>NA<br>0.917<br>0.000
100	0.0	0.070<br>0.000<br>0.022<br>0.020<br>0.930<br>0.000	0.054<br>0.000<br>0.022<br>0.022<br>0.946<br>0.000	0.073<br>0.001<br>0.022<br>0.020<br>0.927<br>0.000	0.026<br>0.001<br>0.021<br>0.024<br>0.974<br>0.000	0.065<br>-0.004<br>0.655<br>0.643<br>0.934<br>0.429	0.463<br>0.372<br>0.197<br>0.199<br>0.537<br>0.178	0.134<br>0.023<br>0.033<br>0.030<br>0.866<br>0.002	0.018<br>0.001<br>0.029<br>0.040<br>0.982<br>0.001	0.062<br>0.000<br>0.023<br>NA<br>0.903<br>0.001
	0.2	0.999<br>0.196<br>0.024<br>0.022<br>0.930<br>0.001	1.000<br>0.196<br>0.024<br>0.025<br>0.945<br>0.001	1.000<br>0.201<br>0.025<br>0.022<br>0.925<br>0.001	1.000<br>0.199<br>0.024<br>0.027<br>0.969<br>0.001	0.070<br>0.152<br>0.654<br>0.642<br>0.939<br>0.429	0.826<br>0.568<br>0.197<br>0.199<br>0.548<br>0.174	1.000<br>0.218<br>0.036<br>0.033<br>0.899<br>0.002	0.970<br>0.163<br>0.030<br>0.040<br>0.875<br>0.002	1.000<br>0.196<br>0.025<br>NA<br>0.907<br>0.001

Table H. In each cell, from top to bottom are empirical type-I error/power, mean($\hat{\theta}$), SD($\hat{\theta}$), mean(SE($\hat{\theta}$)), coverage rate, MSE, when $n = 50\,000$, p_invalid=0.5

m	θ	mixIE-MA	mixIE-MA-DP	cML-MA	cML-MA-DP	Egger	IVW	median	MRMix	ContMix
10	0.0	0.060	0.045	0.086	0.054	0.067	0.136	0.247	0.109	0.096
		0.003	0.010	0.001	0.001	-0.032	0.209	0.051	0.000	0.002
		0.090	0.129	0.026	0.025	1.264	0.274	0.154	0.034	0.042
		0.040	0.089	0.023	0.024	1.057	0.255	0.032	0.029	NA
		0.940	0.955	0.914	0.946	0.891	0.864	0.753	0.891	0.892
		0.008	0.017	0.001	0.001	1.598	0.119	0.026	0.001	0.002
	0.2	0.939	0.822	0.991	0.952	0.068	0.410	0.991	0.975	0.998
		0.201	0.204	0.197	0.188	0.161	0.409	0.252	0.182	0.203
		0.076	0.148	0.036	0.040	1.262	0.273	0.155	0.033	0.053
		0.050	0.098	0.026	0.034	1.056	0.255	0.036	0.032	NA
		0.941	0.957	0.875	0.921	0.892	0.869	0.759	0.851	0.888
		0.006	0.022	0.001	0.002	1.592	0.118	0.027	0.001	0.003
30	0.0	0.076	0.041	0.095	0.029	0.062	0.257	0.216	0.051	0.093
		0.000	0.000	0.000	0.000	-0.026	0.303	0.031	0.000	0.000
		0.023	0.023	0.023	0.021	0.879	0.239	0.058	0.028	0.023
		0.021	0.025	0.020	0.023	0.825	0.229	0.030	0.032	NA
		0.924	0.959	0.905	0.971	0.928	0.743	0.784	0.949	0.905
		0.001	0.001	0.001	0.000	0.773	0.149	0.004	0.001	0.001
	0.2	1.000	1.000	1.000	1.000	0.064	0.608	1.000	0.982	1.000
		0.199	0.199	0.200	0.195	0.161	0.502	0.232	0.175	0.199
		0.026	0.027	0.027	0.027	0.878	0.238	0.062	0.029	0.026
		0.023	0.028	0.022	0.028	0.824	0.228	0.033	0.033	NA
		0.909	0.950	0.895	0.955	0.930	0.746	0.788	0.852	0.913
		0.001	0.001	0.001	0.001	0.771	0.148	0.005	0.001	0.001
100	0.0	0.080	0.045	0.119	0.032	0.048	0.687	0.343	0.037	0.093
		0.001	0.001	0.002	0.003	-0.022	0.613	0.058	0.001	0.002
		0.031	0.031	0.032	0.030	0.834	0.250	0.053	0.032	0.031
		0.028	0.032	0.026	0.032	0.819	0.254	0.039	0.039	NA
		0.920	0.955	0.881	0.968	0.951	0.313	0.657	0.963	0.902
		0.001	0.001	0.001	0.001	0.695	0.437	0.006	0.001	0.001
	0.2	0.997	1.000	1.000	0.999	0.054	0.903	1.000	0.966	1.000
		0.197	0.197	0.204	0.200	0.135	0.808	0.256	0.151	0.198
		0.034	0.034	0.035	0.034	0.832	0.249	0.057	0.032	0.034
		0.031	0.035	0.028	0.037	0.819	0.254	0.042	0.039	NA
		0.913	0.949	0.886	0.958	0.945	0.319	0.695	0.754	0.899
		0.001	0.001	0.001	0.001	0.696	0.432	0.006	0.003	0.001

Table I. In each cell, from top to bottom are empirical type-I error/power, mean($\hat{\theta}$), SD($\hat{\theta}$), mean(SE($\hat{\theta}$)), coverage rate, MSE, when $n = 50\,000$, p_invalid=0.7

m	θ	mixIE-MA	mixIE-MA-DP	cML-MA	cML-MA-DP	Egger	IVW	median	MRMix	ContMix
10	0.0	0.055	0.058	0.154	0.100	0.072	0.185	0.544	0.192	0.225
		0.035	0.027	0.000	0.000	0.026	0.292	0.153	0.003	0.049
		0.514	0.650	0.041	0.037	1.498	0.328	0.293	0.123	0.277
		0.272	0.455	0.029	0.031	1.250	0.300	0.041	0.035	NA
		0.945	0.942	0.846	0.900	0.892	0.815	0.456	0.808	0.890
		0.266	0.423	0.002	0.001	2.241	0.193	0.109	0.015	0.079
	0.2	0.453	0.333	0.920	0.757	0.070	0.410	0.967	0.961	0.989
		0.213	0.213	0.176	0.160	0.220	0.493	0.354	0.181	0.287
		0.518	0.681	0.075	0.069	1.495	0.327	0.293	0.127	0.356
		0.282	0.484	0.033	0.050	1.249	0.300	0.046	0.036	NA
		0.937	0.937	0.732	0.809	0.892	0.815	0.472	0.759	0.899
		0.268	0.464	0.006	0.006	2.232	0.192	0.110	0.016	0.134
30	0.0	0.105	0.037	0.139	0.038	0.054	0.361	0.443	0.096	0.162
		0.001	0.000	0.001	0.002	-0.040	0.419	0.124	0.001	0.002
		0.035	0.036	0.035	0.030	1.031	0.275	0.198	0.038	0.037
		0.030	0.041	0.026	0.031	0.973	0.269	0.042	0.040	NA
		0.895	0.963	0.861	0.962	0.931	0.639	0.557	0.904	0.879
		0.001	0.001	0.001	0.001	1.063	0.251	0.055	0.001	0.001
	0.2	0.997	0.968	0.991	0.936	0.057	0.627	0.994	0.957	0.999
		0.200	0.200	0.199	0.185	0.148	0.618	0.326	0.171	0.201
		0.039	0.040	0.044	0.045	1.028	0.274	0.198	0.038	0.041
		0.032	0.046	0.029	0.044	0.972	0.269	0.046	0.038	NA
		0.889	0.962	0.815	0.947	0.932	0.643	0.574	0.801	0.870
		0.002	0.002	0.002	0.002	1.059	0.250	0.055	0.002	0.002
100	0.0	0.103	0.031	0.187	0.034	0.051	0.824	0.658	0.048	0.149
		-0.001	-0.001	0.004	0.004	-0.033	0.852	0.184	0.000	0.002
		0.047	0.047	0.052	0.045	0.974	0.302	0.166	0.040	0.048
		0.042	0.053	0.034	0.048	0.953	0.296	0.059	0.046	NA
		0.897	0.969	0.813	0.966	0.943	0.176	0.342	0.952	0.891
		0.002	0.002	0.003	0.002	0.948	0.817	0.061	0.002	0.002
	0.2	0.966	0.930	0.990	0.924	0.051	0.940	0.998	0.850	0.996
		0.195	0.194	0.206	0.198	0.124	1.048	0.386	0.141	0.198
		0.052	0.051	0.059	0.055	0.973	0.302	0.171	0.041	0.053
		0.045	0.058	0.037	0.057	0.953	0.296	0.064	0.046	NA
		0.883	0.971	0.793	0.953	0.940	0.183	0.374	0.704	0.890
		0.003	0.003	0.004	0.003	0.952	0.809	0.064	0.005	0.003

Table J. In each cell, from top to bottom are empirical type-I error/power, mean($\hat{\theta}$), SD($\hat{\theta}$), mean(SE($\hat{\theta}$)), coverage rate, MSE, when $n = 50\,000$, p_invalid=1

m	θ	mixIE-MA	mixIE-MA-DP	cML-MA	cML-MA-DP	Egger	IVW	median	MRMix	ContMix
10	0.0	0.274	0.160	0.742	0.533	0.076	0.242	0.901	0.877	0.921
		0.092	0.067	0.071	0.058	-0.007	0.409	0.362	0.102	0.520
		1.181	1.431	0.336	0.289	1.772	0.387	0.471	0.509	1.049
		0.748	1.126	0.040	0.078	1.481	0.356	0.048	0.050	NA
		0.726	0.840	0.258	0.467	0.873	0.758	0.099	0.123	0.114
		1.403	2.050	0.118	0.087	3.137	0.317	0.352	0.270	1.369
	0.2	0.281	0.164	0.760	0.522	0.080	0.415	0.928	0.882	0.933
		0.253	0.254	0.123	0.101	0.187	0.610	0.562	0.196	0.799
		1.171	1.452	0.384	0.329	1.768	0.385	0.469	0.493	1.003
		0.772	1.143	0.044	0.086	1.480	0.355	0.054	0.051	NA
		0.753	0.851	0.159	0.309	0.874	0.757	0.106	0.112	0.115
		1.372	2.109	0.153	0.118	3.122	0.317	0.351	0.243	1.362
30	0.0	0.576	0.231	0.663	0.275	0.071	0.478	0.912	0.855	0.943
		0.333	0.245	0.064	0.035	-0.014	0.597	0.547	0.172	0.788
		0.962	0.774	0.338	0.219	1.235	0.321	0.410	0.559	1.118
		0.364	0.706	0.052	0.098	1.136	0.315	0.062	0.083	NA
		0.424	0.769	0.337	0.725	0.916	0.522	0.088	0.145	0.188
		1.036	0.659	0.118	0.049	1.524	0.460	0.467	0.342	1.870
	0.2	0.609	0.260	0.675	0.276	0.077	0.705	0.958	0.840	0.953
		0.484	0.415	0.110	0.063	0.175	0.797	0.746	0.221	0.998
		0.948	0.749	0.367	0.247	1.233	0.321	0.410	0.533	1.091
		0.353	0.687	0.057	0.105	1.135	0.315	0.068	0.126	NA
		0.404	0.775	0.210	0.433	0.915	0.517	0.094	0.148	0.185
		0.978	0.607	0.142	0.080	1.519	0.459	0.466	0.284	1.826
100	0.0	0.775	0.173	0.799	0.257	0.047	0.945	0.982	0.782	0.958
		0.555	0.590	0.277	0.157	-0.008	1.216	1.111	0.103	1.463
		1.842	1.236	0.682	0.423	1.079	0.345	0.454	0.656	1.537
		0.374	1.300	0.072	0.220	1.112	0.346	0.105	0.209	NA
		0.225	0.827	0.201	0.743	0.946	0.055	0.018	0.218	0.121
		3.698	1.874	0.541	0.203	1.163	1.598	1.440	0.440	4.502
	0.2	0.793	0.178	0.792	0.272	0.049	0.984	0.992	0.768	0.958
		0.629	0.777	0.366	0.208	0.148	1.412	1.304	0.121	1.659
		1.921	1.204	0.707	0.454	1.078	0.345	0.455	0.645	1.525
		0.354	1.296	0.079	0.230	1.112	0.346	0.111	0.218	NA
		0.208	0.836	0.151	0.676	0.950	0.055	0.019	0.236	0.136
		3.872	1.782	0.527	0.206	1.164	1.588	1.426	0.422	4.451

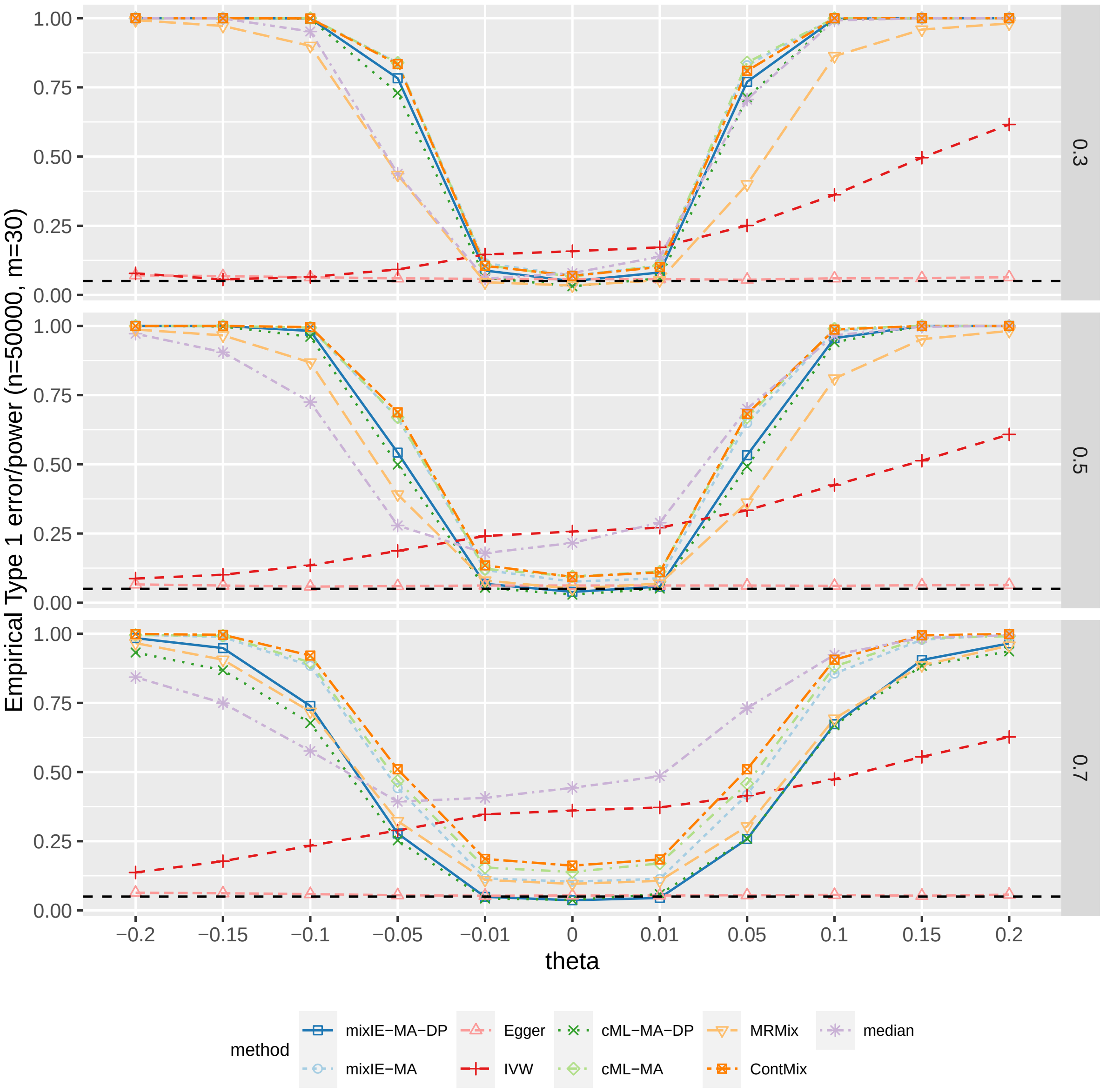

Fig A. Further comparison of power: Empirical type-I error (for $\theta = 0$ and power (for $\theta \neq 0$) curves with sample size $n = 50\,000$ and $m = 30$. Each row corresponds to p_invalid=0.3,0.5,0.7.

B.1.2 Balanced pleiotropy, InSIDE satisfied

Table K. In each cell, from top to bottom are empirical type-I error/power, mean($\hat{\theta}$), SD($\hat{\theta}$), mean(SE($\hat{\theta}$)), coverage rate, MSE, when $n = 10\,000$, p_invalid=0

m	θ	mixIE-MA	mixIE-MA-DP	cML-MA	cML-MA-DP	Egger	IVW	median	MRMix	ContMix
10	0.0	0.054<br>-0.001<br>0.036<br>0.035<br>0.946<br>0.001	0.051<br>-0.001<br>0.035<br>0.036<br>0.949<br>0.001	0.054<br>-0.001<br>0.035<br>0.035<br>0.946<br>0.001	0.032<br>-0.001<br>0.033<br>0.037<br>0.968<br>0.001	0.015<br>0.002<br>0.146<br>0.149<br>0.961<br>0.021	0.045<br>-0.001<br>0.035<br>0.037<br>0.955<br>0.001	0.031<br>-0.001<br>0.041<br>0.046<br>0.969<br>0.002	0.075<br>-0.001<br>0.063<br>0.067<br>0.925<br>0.004	0.058<br>-0.002<br>0.065<br>NA<br>0.928<br>0.004
	0.2	0.992<br>0.197<br>0.040<br>0.039<br>0.936<br>0.002	0.986<br>0.197<br>0.040<br>0.041<br>0.946<br>0.002	0.996<br>0.198<br>0.040<br>0.040<br>0.934<br>0.002	0.992<br>0.194<br>0.041<br>0.044<br>0.950<br>0.002	0.132<br>0.170<br>0.164<br>0.167<br>0.956<br>0.028	0.994<br>0.197<br>0.039<br>0.041<br>0.947<br>0.002	0.964<br>0.195<br>0.047<br>0.052<br>0.965<br>0.002	0.785<br>0.173<br>0.075<br>0.133<br>0.881<br>0.006	0.967<br>0.229<br>0.054<br>NA<br>0.916<br>0.004
30	0.0	0.050<br>-0.001<br>0.030<br>0.030<br>0.950<br>0.001	0.034<br>0.000<br>0.029<br>0.033<br>0.966<br>0.001	0.047<br>0.000<br>0.029<br>0.030<br>0.953<br>0.001	0.018<br>0.000<br>0.028<br>0.034<br>0.982<br>0.001	0.041<br>-0.002<br>0.097<br>0.098<br>0.946<br>0.009	0.044<br>0.000<br>0.028<br>0.030<br>0.956<br>0.001	0.027<br>0.000<br>0.037<br>0.042<br>0.973<br>0.001	0.030<br>-0.004<br>0.052<br>0.086<br>0.970<br>0.003	0.072<br>-0.001<br>0.056<br>NA<br>0.911<br>0.003
	0.2	0.995<br>0.193<br>0.034<br>0.034<br>0.941<br>0.001	0.998<br>0.194<br>0.033<br>0.038<br>0.960<br>0.001	1.000<br>0.200<br>0.033<br>0.034<br>0.946<br>0.001	1.000<br>0.195<br>0.033<br>0.040<br>0.972<br>0.001	0.259<br>0.143<br>0.112<br>0.110<br>0.919<br>0.016	1.000<br>0.195<br>0.032<br>0.034<br>0.951<br>0.001	0.984<br>0.191<br>0.042<br>0.048<br>0.962<br>0.002	0.726<br>0.174<br>0.074<br>0.127<br>0.934<br>0.006	0.991<br>0.235<br>0.054<br>NA<br>0.861<br>0.004
100	0.0	0.081<br>0.000<br>0.045<br>0.030<br>0.919<br>0.002	0.046<br>0.000<br>0.033<br>0.040<br>0.954<br>0.001	0.072<br>0.000<br>0.036<br>0.032<br>0.928<br>0.001	0.019<br>0.000<br>0.035<br>0.042<br>0.981<br>0.001	0.033<br>0.000<br>0.073<br>0.076<br>0.964<br>0.005	0.057<br>0.000<br>0.032<br>0.031<br>0.943<br>0.001	0.035<br>-0.001<br>0.041<br>0.046<br>0.965<br>0.002	0.029<br>0.004<br>0.067<br>0.574<br>0.971<br>0.005	0.069<br>0.001<br>0.043<br>NA<br>0.900<br>0.002
	0.2	0.993<br>0.181<br>0.048<br>0.035<br>0.872<br>0.003	0.892<br>0.181<br>0.038<br>0.049<br>0.925<br>0.002	0.999<br>0.199<br>0.041<br>0.037<br>0.930<br>0.002	0.995<br>0.193<br>0.040<br>0.048<br>0.980<br>0.002	0.151<br>0.083<br>0.082<br>0.085<br>0.710<br>0.020	0.998<br>0.180<br>0.036<br>0.035<br>0.891<br>0.002	0.943<br>0.170<br>0.045<br>0.052<br>0.937<br>0.003	0.672<br>0.180<br>0.080<br>0.172<br>0.938<br>0.007	0.988<br>0.186<br>0.048<br>NA<br>0.883<br>0.002

Table L. In each cell, from top to bottom are empirical type-I error/power, mean($\hat{\theta}$), SD($\hat{\theta}$), mean(SE($\hat{\theta}$)), coverage rate, MSE, when $n = 10\,000$, p_invalid=0.3

m	θ	mixIE-MA	mixIE-MA-DP	cML-MA	cML-MA-DP	Egger	IVW	median	MRMix	ContMix
10	0.0	0.069<br>-0.001<br>0.077<br>0.055<br>0.931<br>0.006	0.051<br>-0.005<br>0.125<br>0.100<br>0.949<br>0.016	0.066<br>0.000<br>0.047<br>0.043<br>0.934<br>0.002	0.034<br>0.000<br>0.043<br>0.047<br>0.966<br>0.002	0.044<br>-0.030<br>1.273<br>1.056<br>0.932<br>1.619	0.058<br>-0.011<br>0.299<br>0.265<br>0.942<br>0.089	0.088<br>-0.004<br>0.082<br>0.056<br>0.912<br>0.007	0.064<br>0.001<br>0.059<br>0.060<br>0.936<br>0.003	0.074<br>0.000<br>0.053<br>NA<br>0.934<br>0.003
	0.2	0.897<br>0.188<br>0.163<br>0.072<br>0.915<br>0.027	0.721<br>0.188<br>0.143<br>0.123<br>0.946<br>0.021	0.965<br>0.199<br>0.056<br>0.049<br>0.919<br>0.003	0.866<br>0.188<br>0.056<br>0.059<br>0.956<br>0.003	0.047<br>0.140<br>1.264<br>1.056<br>0.934<br>1.599	0.179<br>0.189<br>0.296<br>0.265<br>0.941<br>0.087	0.858<br>0.194<br>0.089<br>0.063<br>0.920<br>0.008	0.847<br>0.184<br>0.062<br>0.063<br>0.920<br>0.004	0.948<br>0.201<br>0.060<br>NA<br>0.933<br>0.004
30	0.0	0.080<br>0.001<br>0.043<br>0.039<br>0.920<br>0.002	0.052<br>0.001<br>0.043<br>0.045<br>0.948<br>0.002	0.082<br>0.000<br>0.043<br>0.039<br>0.918<br>0.002	0.024<br>0.001<br>0.040<br>0.046<br>0.976<br>0.002	0.067<br>-0.001<br>0.851<br>0.790<br>0.921<br>0.723	0.061<br>-0.008<br>0.273<br>0.244<br>0.939<br>0.075	0.074<br>0.000<br>0.061<br>0.054<br>0.926<br>0.004	0.035<br>0.000<br>0.050<br>0.065<br>0.965<br>0.003	0.071<br>0.001<br>0.045<br>NA<br>0.914<br>0.002
	0.2	0.984<br>0.196<br>0.048<br>0.043<br>0.917<br>0.002	0.972<br>0.195<br>0.048<br>0.049<br>0.953<br>0.002	0.991<br>0.201<br>0.049<br>0.043<br>0.919<br>0.002	0.948<br>0.192<br>0.048<br>0.055<br>0.966<br>0.002	0.068<br>0.146<br>0.845<br>0.789<br>0.923<br>0.716	0.169<br>0.189<br>0.271<br>0.243<br>0.939<br>0.074	0.889<br>0.192<br>0.066<br>0.060<br>0.934<br>0.004	0.794<br>0.175<br>0.052<br>0.067<br>0.937<br>0.003	0.983<br>0.196<br>0.049<br>NA<br>0.920<br>0.002
100	0.0	0.092<br>-0.001<br>0.057<br>0.052<br>0.908<br>0.003	0.065<br>-0.001<br>0.058<br>0.056<br>0.935<br>0.003	0.128<br>0.000<br>0.064<br>0.050<br>0.872<br>0.004	0.020<br>-0.001<br>0.060<br>0.070<br>0.980<br>0.004	0.051<br>0.037<br>0.655<br>0.640<br>0.947<br>0.430	0.058<br>0.002<br>0.268<br>0.261<br>0.942<br>0.072	0.058<br>-0.001<br>0.073<br>0.069<br>0.942<br>0.005	0.028<br>0.001<br>0.064<br>0.077<br>0.972<br>0.004	0.094<br>-0.001<br>0.058<br>NA<br>0.904<br>0.003
	0.2	0.879<br>0.180<br>0.062<br>0.055<br>0.895<br>0.004	0.843<br>0.179<br>0.063<br>0.060<br>0.927<br>0.004	0.924<br>0.204<br>0.071<br>0.054<br>0.867<br>0.005	0.739<br>0.194<br>0.067<br>0.078<br>0.970<br>0.005	0.056<br>0.120<br>0.653<br>0.640<br>0.949<br>0.432	0.123<br>0.184<br>0.267<br>0.261<br>0.942<br>0.072	0.646<br>0.172<br>0.079<br>0.075<br>0.914<br>0.007	0.491<br>0.149<br>0.066<br>0.081<br>0.914<br>0.007	0.892<br>0.178<br>0.063<br>NA<br>0.880<br>0.004

Table M. In each cell, from top to bottom are empirical type-I error/power, mean($\hat{\theta}$), SD($\hat{\theta}$), mean(SE($\hat{\theta}$)), coverage rate, MSE, when $n = 10\,000$, p_invalid=0.5

m	θ	mixIE-MA	mixIE-MA-DP	cML-MA	cML-MA-DP	Egger	IVW	median	MRMix	ContMix
10	0.0	0.062<br>0.008<br>0.247<br>0.111<br>0.938<br>0.061	0.048<br>-0.005<br>0.400<br>0.242<br>0.952<br>0.160	0.105<br>0.005<br>0.063<br>0.052<br>0.895<br>0.004	0.045<br>0.004<br>0.056<br>0.057<br>0.955<br>0.003	0.054<br>-0.036<br>1.629<br>1.409<br>0.908<br>2.652	0.085<br>-0.014<br>0.385<br>0.353<br>0.915<br>0.149	0.211<br>-0.003<br>0.180<br>0.069<br>0.789<br>0.033	0.102<br>0.005<br>0.078<br>0.068<br>0.899<br>0.006	0.110<br>0.007<br>0.104<br>NA<br>0.929<br>0.011
	0.2	0.673<br>0.186<br>0.336<br>0.150<br>0.925<br>0.113	0.468<br>0.182<br>0.443<br>0.286<br>0.947<br>0.197	0.860<br>0.200<br>0.077<br>0.058<br>0.869<br>0.006	0.675<br>0.181<br>0.074<br>0.074<br>0.931<br>0.006	0.057<br>0.134<br>1.620<br>1.407<br>0.914<br>2.626	0.129<br>0.187<br>0.382<br>0.353<br>0.919<br>0.146	0.756<br>0.197<br>0.185<br>0.076<br>0.793<br>0.034	0.752<br>0.183<br>0.078<br>0.072<br>0.879<br>0.006	0.880<br>0.207<br>0.109<br>NA<br>0.922<br>0.012
30	0.0	0.101<br>0.003<br>0.060<br>0.051<br>0.899<br>0.004	0.045<br>0.003<br>0.061<br>0.063<br>0.955<br>0.004	0.125<br>0.003<br>0.061<br>0.048<br>0.875<br>0.004	0.036<br>0.003<br>0.054<br>0.060<br>0.964<br>0.003	0.059<br>0.006<br>1.082<br>1.028<br>0.931<br>1.169	0.070<br>-0.020<br>0.341<br>0.317<br>0.931<br>0.117	0.139<br>0.000<br>0.100<br>0.066<br>0.861<br>0.010	0.066<br>0.002<br>0.061<br>0.069<br>0.934<br>0.004	0.111<br>0.003<br>0.062<br>NA<br>0.909<br>0.004
	0.2	0.911<br>0.198<br>0.067<br>0.056<br>0.893<br>0.004	0.803<br>0.198<br>0.068<br>0.070<br>0.946<br>0.005	0.933<br>0.204<br>0.070<br>0.053<br>0.856<br>0.005	0.731<br>0.187<br>0.067<br>0.073<br>0.945<br>0.005	0.057<br>0.153<br>1.075<br>1.026<br>0.937<br>1.157	0.122<br>0.178<br>0.339<br>0.316<br>0.932<br>0.115	0.738<br>0.193<br>0.108<br>0.072<br>0.864<br>0.012	0.701<br>0.171<br>0.066<br>0.071<br>0.894<br>0.005	0.938<br>0.199<br>0.070<br>NA<br>0.900<br>0.005
100	0.0	0.108<br>-0.001<br>0.080<br>0.068<br>0.892<br>0.006	0.047<br>0.000<br>0.082<br>0.086<br>0.953<br>0.007	0.188<br>0.000<br>0.098<br>0.065<br>0.812<br>0.010	0.017<br>-0.001<br>0.086<br>0.103<br>0.983<br>0.007	0.053<br>0.011<br>0.833<br>0.817<br>0.942<br>0.693	0.058<br>-0.008<br>0.343<br>0.334<br>0.942<br>0.118	0.116<br>-0.003<br>0.112<br>0.089<br>0.884<br>0.013	0.040<br>0.001<br>0.073<br>0.085<br>0.960<br>0.005	0.132<br>0.001<br>0.083<br>NA<br>0.899<br>0.007
	0.2	0.685<br>0.179<br>0.086<br>0.072<br>0.874<br>0.008	0.525<br>0.180<br>0.087<br>0.092<br>0.949<br>0.008	0.777<br>0.212<br>0.107<br>0.069<br>0.795<br>0.011	0.388<br>0.195<br>0.095<br>0.114<br>0.974<br>0.009	0.058<br>0.094<br>0.832<br>0.817<br>0.944<br>0.703	0.086<br>0.174<br>0.342<br>0.334<br>0.939<br>0.118	0.461<br>0.171<br>0.117<br>0.094<br>0.879<br>0.015	0.363<br>0.137<br>0.076<br>0.087<br>0.886<br>0.010	0.751<br>0.181<br>0.089<br>NA<br>0.883<br>0.008

Table N. In each cell, from top to bottom are empirical type-I error/power, mean($\hat{\theta}$), SD($\hat{\theta}$), mean(SE($\hat{\theta}$)), coverage rate, MSE, when $n = 10\,000$, p_invalid=0.7

m	θ	mixIE-MA	mixIE-MA-DP	cML-MA	cML-MA-DP	Egger	IVW	median	MRMix	ContMix
10	0.0	0.083	0.059	0.158	0.079	0.070	0.079	0.402	0.212	0.253
		0.011	-0.003	-0.002	-0.002	0.041	-0.014	-0.007	-0.004	-0.007
		0.718	0.964	0.102	0.085	2.008	0.453	0.362	0.186	0.472
		0.395	0.749	0.067	0.076	1.699	0.424	0.084	0.088	NA
		0.917	0.941	0.842	0.921	0.890	0.921	0.598	0.788	0.856
		0.516	0.929	0.010	0.007	4.031	0.205	0.131	0.035	0.223
	0.2	0.276	0.156	0.648	0.399	0.069	0.113	0.696	0.646	0.765
		0.185	0.168	0.180	0.153	0.211	0.189	0.192	0.168	0.209
		0.782	1.035	0.129	0.106	1.997	0.450	0.361	0.190	0.486
		0.454	0.816	0.074	0.095	1.695	0.423	0.091	0.088	NA
		0.922	0.951	0.795	0.856	0.888	0.920	0.598	0.758	0.843
		0.611	1.071	0.017	0.014	3.984	0.202	0.131	0.037	0.236
30	0.0	0.108	0.042	0.186	0.038	0.042	0.089	0.245	0.079	0.183
		0.004	0.002	0.005	0.005	-0.018	-0.035	-0.001	0.007	0.004
		0.106	0.109	0.093	0.077	1.241	0.397	0.198	0.086	0.110
		0.076	0.111	0.062	0.083	1.223	0.374	0.084	0.089	NA
		0.892	0.958	0.814	0.962	0.944	0.911	0.755	0.921	0.889
		0.011	0.012	0.009	0.006	1.538	0.158	0.039	0.008	0.012
	0.2	0.711	0.470	0.753	0.399	0.049	0.102	0.624	0.560	0.782
		0.202	0.196	0.202	0.173	0.131	0.164	0.192	0.168	0.207
		0.117	0.123	0.110	0.096	1.235	0.395	0.204	0.090	0.126
		0.083	0.123	0.067	0.101	1.222	0.374	0.091	0.088	NA
		0.890	0.955	0.764	0.930	0.945	0.912	0.760	0.878	0.893
		0.014	0.015	0.012	0.010	1.529	0.157	0.042	0.009	0.016
100	0.0	0.156	0.041	0.333	0.023	0.062	0.069	0.211	0.077	0.294
		-0.007	-0.008	-0.011	-0.009	0.044	-0.004	-0.003	-0.003	-0.004
		0.138	0.143	0.207	0.154	1.031	0.413	0.196	0.109	0.150
		0.100	0.157	0.090	0.173	0.962	0.393	0.117	0.116	NA
		0.844	0.959	0.667	0.977	0.935	0.931	0.789	0.923	0.839
		0.019	0.020	0.043	0.024	1.063	0.171	0.038	0.012	0.022
	0.2	0.450	0.177	0.585	0.137	0.069	0.092	0.348	0.230	0.558
		0.175	0.171	0.220	0.188	0.127	0.178	0.168	0.122	0.173
		0.149	0.151	0.224	0.169	1.028	0.413	0.202	0.110	0.155
		0.103	0.166	0.095	0.187	0.962	0.393	0.122	0.115	NA
		0.813	0.961	0.628	0.964	0.932	0.929	0.796	0.867	0.828
		0.023	0.023	0.050	0.029	1.062	0.171	0.042	0.018	0.025

Table O. In each cell, from top to bottom are empirical type-I error/power, mean($\hat{\theta}$), SD($\hat{\theta}$), mean(SE($\hat{\theta}$)), coverage rate, MSE, when $n = 10\,000$, p_invalid=1

m	θ	mixIE-MA	mixIE-MA-DP	cML-MA	cML-MA-DP	Egger	IVW	median	MRMix	ContMix
10	0.0	0.203	0.101	0.656	0.336	0.056	0.091	0.765	0.728	0.870
		0.012	0.011	0.008	0.011	0.018	-0.024	-0.006	0.019	-0.034
		1.379	1.769	0.545	0.394	2.258	0.548	0.647	0.566	1.390
		1.003	1.632	0.097	0.170	2.038	0.511	0.103	0.127	NA
		0.797	0.899	0.344	0.664	0.909	0.909	0.235	0.272	0.268
		1.899	3.125	0.297	0.155	5.092	0.300	0.418	0.320	1.931
	0.2	0.210	0.097	0.654	0.315	0.061	0.107	0.766	0.737	0.883
		0.168	0.180	0.080	0.052	0.187	0.180	0.198	0.061	0.227
		1.412	1.806	0.564	0.409	2.248	0.545	0.646	0.568	1.395
		1.051	1.661	0.105	0.181	2.035	0.510	0.109	0.152	NA
		0.801	0.908	0.296	0.555	0.909	0.911	0.251	0.252	0.271
		1.994	3.260	0.332	0.189	5.049	0.297	0.417	0.341	1.944
30	0.0	0.585	0.159	0.688	0.188	0.053	0.065	0.689	0.635	0.881
		0.018	-0.012	-0.012	-0.002	0.048	-0.038	-0.028	0.024	0.010
		1.056	0.789	0.657	0.373	1.460	0.476	0.595	0.572	1.488
		0.357	0.795	0.114	0.227	1.451	0.446	0.123	0.243	NA
		0.415	0.841	0.312	0.812	0.941	0.935	0.311	0.365	0.211
		1.115	0.622	0.431	0.139	2.132	0.228	0.355	0.328	2.212
	0.2	0.609	0.156	0.673	0.188	0.055	0.084	0.695	0.669	0.894
		0.150	0.152	0.062	0.030	0.195	0.162	0.168	0.075	0.253
		1.000	0.785	0.674	0.382	1.454	0.474	0.591	0.586	1.484
		0.334	0.811	0.123	0.237	1.449	0.445	0.129	0.216	NA
		0.412	0.850	0.291	0.730	0.944	0.935	0.316	0.327	0.220
		1.002	0.618	0.473	0.175	2.112	0.226	0.350	0.358	2.203
100	0.0	0.735	0.092	0.924	0.218	0.061	0.067	0.577	0.518	0.906
		0.019	-0.103	0.036	-0.027	0.004	-0.004	0.003	0.012	0.031
		2.956	1.696	2.704	1.195	1.175	0.491	0.629	0.724	1.783
		0.523	2.004	0.305	0.844	1.146	0.469	0.191	0.636	NA
		0.265	0.908	0.076	0.782	0.936	0.933	0.423	0.482	0.227
		8.727	2.885	7.308	1.428	1.380	0.241	0.396	0.524	3.177
	0.2	0.732	0.090	0.920	0.212	0.063	0.081	0.598	0.546	0.913
		-0.007	0.030	0.359	0.115	0.088	0.179	0.177	0.028	0.242
		2.908	1.673	2.610	1.165	1.175	0.490	0.629	0.726	1.781
		0.506	1.976	0.316	0.833	1.146	0.469	0.196	0.600	NA
		0.276	0.909	0.085	0.788	0.937	0.935	0.435	0.464	0.243
		8.493	2.826	6.829	1.364	1.391	0.241	0.396	0.556	3.171

Table P. In each cell, from top to bottom are empirical type-I error/power, mean($\hat{\theta}$), SD($\hat{\theta}$), mean(SE($\hat{\theta}$)), coverage rate, MSE, when $n = 50\,000$, p_invalid=0

m	θ	mixIE-MA	mixIE-MA-DP	cML-MA	cML-MA-DP	Egger	IVW	median	MRMix	ContMix
10	0.0	0.046	0.041	0.046	0.033	0.012	0.039	0.033	0.077	0.063
		0.000	0.000	0.000	0.000	0.002	0.000	0.000	0.000	0.000
		0.015	0.015	0.015	0.015	0.068	0.015	0.019	0.027	0.029
		0.015	0.016	0.015	0.016	0.070	0.016	0.021	0.040	NA
		0.954	0.959	0.954	0.967	0.963	0.961	0.967	0.923	0.895
		0.000	0.000	0.000	0.000	0.005	0.000	0.000	0.001	0.001
	0.2	1.000	0.999	1.000	1.000	0.616	1.000	1.000	0.885	1.000
		0.200	0.200	0.200	0.199	0.195	0.200	0.200	0.173	0.208
		0.018	0.018	0.018	0.018	0.077	0.018	0.022	0.054	0.021
		0.017	0.018	0.018	0.019	0.079	0.019	0.023	0.794	NA
		0.938	0.949	0.945	0.953	0.960	0.954	0.962	0.809	0.883
		0.000	0.000	0.000	0.000	0.006	0.000	0.000	0.004	0.001
30	0.0	0.051	0.037	0.049	0.023	0.037	0.043	0.024	0.043	0.078
		0.000	0.000	0.000	0.000	-0.001	0.000	0.000	0.000	0.000
		0.013	0.013	0.013	0.013	0.049	0.013	0.017	0.025	0.027
		0.013	0.014	0.013	0.014	0.049	0.014	0.019	0.052	NA
		0.949	0.963	0.951	0.977	0.954	0.957	0.976	0.957	0.869
		0.000	0.000	0.000	0.000	0.002	0.000	0.000	0.001	0.001
	0.2	1.000	1.000	1.000	1.000	0.877	1.000	1.000	0.830	1.000
		0.199	0.199	0.200	0.199	0.185	0.199	0.198	0.173	0.217
		0.015	0.015	0.015	0.015	0.056	0.015	0.019	0.062	0.019
		0.015	0.016	0.015	0.017	0.056	0.015	0.021	0.062	NA
		0.942	0.957	0.954	0.965	0.952	0.952	0.976	0.833	0.752
		0.000	0.000	0.000	0.000	0.003	0.000	0.000	0.005	0.001
100	0.0	0.047	0.047	0.044	0.018	0.046	0.043	0.025	0.021	0.076
		0.000	0.000	0.000	0.000	0.000	0.000	0.000	-0.001	0.001
		0.014	0.014	0.014	0.014	0.047	0.014	0.018	0.026	0.029
		0.014	0.015	0.014	0.016	0.047	0.014	0.021	0.075	NA
		0.953	0.953	0.956	0.982	0.953	0.957	0.975	0.979	0.887
		0.000	0.000	0.000	0.000	0.002	0.000	0.000	0.001	0.001
	0.2	1.000	1.000	1.000	1.000	0.827	1.000	1.000	0.794	1.000
		0.196	0.196	0.200	0.198	0.155	0.196	0.194	0.176	0.236
		0.017	0.016	0.017	0.017	0.053	0.016	0.021	0.054	0.025
		0.016	0.016	0.016	0.019	0.053	0.016	0.024	0.143	NA
		0.937	0.947	0.949	0.977	0.852	0.949	0.967	0.896	0.552
		0.000	0.000	0.000	0.000	0.005	0.000	0.000	0.004	0.002

Table Q. In each cell, from top to bottom are empirical type-I error/power, mean($\hat{\theta}$), SD($\hat{\theta}$), mean(SE($\hat{\theta}$)), coverage rate, MSE, when $n = 50\,000$, p_invalid=0.3

m	θ	mixIE-MA	mixIE-MA-DP	cML-MA	cML-MA-DP	Egger	IVW	median	MRMix	ContMix
10	0.0	0.066	0.070	0.067	0.051	0.066	0.040	0.100	0.067	0.081
		0.003	0.002	0.000	0.000	-0.024	0.000	0.000	-0.001	0.000
		0.067	0.073	0.020	0.020	1.416	0.296	0.074	0.026	0.022
		0.027	0.033	0.019	0.020	1.121	0.264	0.026	0.026	NA
		0.934	0.930	0.933	0.949	0.891	0.960	0.900	0.933	0.916
		0.004	0.005	0.000	0.000	2.003	0.088	0.006	0.001	0.000
	0.2	0.995	0.943	1.000	0.999	0.075	0.198	0.991	0.990	1.000
		0.201	0.200	0.199	0.196	0.171	0.202	0.200	0.181	0.200
		0.057	0.084	0.024	0.025	1.404	0.293	0.074	0.028	0.025
		0.025	0.041	0.022	0.025	1.118	0.264	0.029	0.027	NA
		0.925	0.925	0.925	0.944	0.892	0.964	0.905	0.859	0.918
		0.003	0.007	0.001	0.001	1.970	0.086	0.006	0.001	0.001
30	0.0	0.056	0.039	0.054	0.025	0.063	0.073	0.068	0.030	0.068
		0.000	0.000	0.000	0.000	0.003	0.000	0.000	0.000	0.000
		0.018	0.018	0.018	0.017	0.927	0.259	0.027	0.025	0.019
		0.017	0.019	0.017	0.019	0.870	0.240	0.024	0.031	NA
		0.944	0.961	0.946	0.975	0.928	0.927	0.932	0.970	0.896
		0.000	0.000	0.000	0.000	0.859	0.067	0.001	0.001	0.000
	0.2	1.000	1.000	1.000	1.000	0.065	0.163	1.000	0.984	1.000
		0.199	0.199	0.200	0.198	0.190	0.201	0.198	0.175	0.199
		0.021	0.021	0.021	0.020	0.920	0.257	0.030	0.026	0.021
		0.019	0.021	0.019	0.022	0.867	0.239	0.027	0.031	NA
		0.933	0.950	0.936	0.960	0.927	0.928	0.930	0.860	0.903
		0.000	0.000	0.000	0.000	0.846	0.066	0.001	0.001	0.000
100	0.0	0.067	0.046	0.067	0.022	0.059	0.064	0.078	0.017	0.066
		0.000	0.000	0.000	0.000	-0.013	0.010	0.000	0.000	-0.001
		0.024	0.024	0.024	0.023	0.889	0.280	0.035	0.029	0.024
		0.022	0.024	0.022	0.026	0.872	0.270	0.033	0.040	NA
		0.933	0.954	0.933	0.978	0.938	0.936	0.922	0.983	0.907
		0.001	0.001	0.001	0.001	0.789	0.078	0.001	0.001	0.001
	0.2	1.000	1.000	1.000	1.000	0.060	0.143	0.999	0.949	1.000
		0.195	0.195	0.200	0.197	0.144	0.207	0.193	0.150	0.195
		0.026	0.026	0.026	0.026	0.885	0.278	0.039	0.030	0.026
		0.024	0.026	0.024	0.029	0.870	0.269	0.035	0.041	NA
		0.937	0.948	0.934	0.971	0.937	0.936	0.921	0.770	0.900
		0.001	0.001	0.001	0.001	0.786	0.078	0.002	0.003	0.001

Table R. In each cell, from top to bottom are empirical type-I error/power, mean($\hat{\theta}$), SD($\hat{\theta}$), mean(SE($\hat{\theta}$)), coverage rate, MSE, when $n = 50\,000$, p_invalid=0.5

m	θ	mixIE-MA	mixIE-MA-DP	cML-MA	cML-MA-DP	Egger	IVW	median	MRMix	ContMix
10	0.0	0.070<br>-0.003<br>0.200<br>0.040<br>0.930<br>0.040	0.051<br>0.000<br>0.169<br>0.084<br>0.949<br>0.028	0.093<br>0.000<br>0.027<br>0.023<br>0.907<br>0.001	0.071<br>0.000<br>0.025<br>0.024<br>0.929<br>0.001	0.069<br>-0.052<br>1.740<br>1.444<br>0.900<br>3.028	0.077<br>-0.003<br>0.392<br>0.348<br>0.923<br>0.153	0.218<br>0.002<br>0.173<br>0.033<br>0.782<br>0.030	0.104<br>0.001<br>0.033<br>0.039<br>0.896<br>0.001	0.101<br>0.000<br>0.027<br>NA<br>0.904<br>0.001
	0.2	0.950<br>0.193<br>0.153<br>0.050<br>0.924<br>0.024	0.865<br>0.191<br>0.132<br>0.088<br>0.951<br>0.018	0.992<br>0.197<br>0.036<br>0.026<br>0.882<br>0.001	0.965<br>0.189<br>0.038<br>0.033<br>0.918<br>0.002	0.071<br>0.141<br>1.730<br>1.441<br>0.898<br>2.993	0.130<br>0.200<br>0.389<br>0.347<br>0.921<br>0.151	0.964<br>0.202<br>0.174<br>0.036<br>0.777<br>0.030	0.982<br>0.179<br>0.033<br>0.029<br>0.801<br>0.002	0.999<br>0.201<br>0.031<br>NA<br>0.905<br>0.001
30	0.0	0.072<br>0.000<br>0.024<br>0.022<br>0.928<br>0.001	0.047<br>0.000<br>0.025<br>0.026<br>0.953<br>0.001	0.085<br>0.000<br>0.024<br>0.021<br>0.915<br>0.001	0.029<br>0.000<br>0.022<br>0.024<br>0.971<br>0.000	0.058<br>-0.020<br>1.212<br>1.138<br>0.930<br>1.468	0.079<br>-0.008<br>0.340<br>0.314<br>0.921<br>0.115	0.150<br>-0.001<br>0.052<br>0.031<br>0.850<br>0.003	0.038<br>0.000<br>0.028<br>0.034<br>0.962<br>0.001	0.091<br>0.000<br>0.025<br>NA<br>0.908<br>0.001
	0.2	1.000<br>0.199<br>0.027<br>0.024<br>0.921<br>0.001	1.000<br>0.199<br>0.027<br>0.028<br>0.958<br>0.001	1.000<br>0.200<br>0.027<br>0.024<br>0.906<br>0.001	1.000<br>0.195<br>0.028<br>0.029<br>0.954<br>0.001	0.062<br>0.168<br>1.206<br>1.134<br>0.932<br>1.453	0.120<br>0.194<br>0.337<br>0.314<br>0.923<br>0.114	0.984<br>0.198<br>0.056<br>0.034<br>0.844<br>0.003	0.974<br>0.167<br>0.030<br>0.035<br>0.773<br>0.002	1.000<br>0.199<br>0.027<br>NA<br>0.907<br>0.001
100	0.0	0.073<br>0.001<br>0.035<br>0.032<br>0.927<br>0.001	0.044<br>0.001<br>0.035<br>0.036<br>0.956<br>0.001	0.106<br>0.001<br>0.036<br>0.029<br>0.894<br>0.001	0.028<br>0.001<br>0.033<br>0.036<br>0.972<br>0.001	0.054<br>-0.036<br>1.151<br>1.124<br>0.943<br>1.324	0.057<br>0.010<br>0.355<br>0.347<br>0.943<br>0.126	0.130<br>0.003<br>0.056<br>0.043<br>0.870<br>0.003	0.027<br>0.002<br>0.031<br>0.040<br>0.973<br>0.001	0.084<br>0.001<br>0.035<br>NA<br>0.905<br>0.001
	0.2	0.997<br>0.197<br>0.037<br>0.033<br>0.924<br>0.001	0.996<br>0.197<br>0.037<br>0.038<br>0.954<br>0.001	0.999<br>0.203<br>0.038<br>0.031<br>0.894<br>0.001	0.996<br>0.198<br>0.037<br>0.041<br>0.965<br>0.001	0.052<br>0.121<br>1.148<br>1.123<br>0.943<br>1.322	0.097<br>0.208<br>0.354<br>0.347<br>0.942<br>0.125	0.955<br>0.196<br>0.059<br>0.046<br>0.881<br>0.003	0.928<br>0.137<br>0.032<br>0.041<br>0.637<br>0.005	1.000<br>0.196<br>0.037<br>NA<br>0.892<br>0.001

Table S. In each cell, from top to bottom are empirical type-I error/power, mean($\hat{\theta}$), SD($\hat{\theta}$), mean(SE($\hat{\theta}$)), coverage rate, MSE, when $n = 50\,000$, p_invalid=0.7

m	θ	mixIE-MA	mixIE-MA-DP	cML-MA	cML-MA-DP	Egger	IVW	median	MRMix	ContMix
10	0.0	0.064<br>-0.013<br>0.644<br>0.322<br>0.936<br>0.414	0.058<br>0.001<br>0.908<br>0.553<br>0.942<br>0.823	0.130<br>-0.001<br>0.039<br>0.030<br>0.870<br>0.002	0.076<br>-0.001<br>0.036<br>0.032<br>0.924<br>0.001	0.065<br>0.029<br>2.099<br>1.735<br>0.886<br>4.403	0.095<br>-0.003<br>0.468<br>0.415<br>0.905<br>0.219	0.453<br>-0.008<br>0.359<br>0.045<br>0.547<br>0.129	0.150<br>-0.003<br>0.090<br>0.035<br>0.850<br>0.008	0.194<br>-0.014<br>0.263<br>NA<br>0.905<br>0.069
	0.2	0.467<br>0.169<br>0.709<br>0.349<br>0.933<br>0.504	0.329<br>0.177<br>0.912<br>0.575<br>0.934<br>0.831	0.919<br>0.177<br>0.073<br>0.034<br>0.770<br>0.006	0.765<br>0.163<br>0.067<br>0.050<br>0.833<br>0.006	0.065<br>0.222<br>2.089<br>1.731<br>0.886<br>4.359	0.117<br>0.201<br>0.465<br>0.414<br>0.909<br>0.216	0.901<br>0.194<br>0.357<br>0.048<br>0.569<br>0.127	0.965<br>0.165<br>0.114<br>0.034<br>0.738<br>0.014	0.984<br>0.199<br>0.312<br>NA<br>0.892<br>0.097
30	0.0	0.092<br>0.000<br>0.037<br>0.031<br>0.908<br>0.001	0.044<br>-0.001<br>0.039<br>0.042<br>0.956<br>0.002	0.128<br>-0.001<br>0.038<br>0.029<br>0.872<br>0.001	0.054<br>-0.001<br>0.033<br>0.033<br>0.946<br>0.001	0.064<br>-0.047<br>1.454<br>1.358<br>0.922<br>2.113	0.064<br>-0.017<br>0.391<br>0.374<br>0.936<br>0.153	0.285<br>-0.009<br>0.164<br>0.042<br>0.715<br>0.027	0.090<br>0.000<br>0.037<br>0.038<br>0.910<br>0.001	0.145<br>0.000<br>0.038<br>NA<br>0.905<br>0.001
	0.2	0.991<br>0.199<br>0.042<br>0.034<br>0.895<br>0.002	0.962<br>0.198<br>0.043<br>0.047<br>0.950<br>0.002	0.995<br>0.196<br>0.046<br>0.031<br>0.820<br>0.002	0.908<br>0.182<br>0.048<br>0.045<br>0.912<br>0.003	0.065<br>0.142<br>1.447<br>1.355<br>0.923<br>2.094	0.104<br>0.185<br>0.389<br>0.374<br>0.938<br>0.152	0.901<br>0.190<br>0.167<br>0.045<br>0.726<br>0.028	0.936<br>0.161<br>0.039<br>0.041<br>0.734<br>0.003	0.997<br>0.199<br>0.042<br>NA<br>0.890<br>0.002
100	0.0	0.093<br>0.001<br>0.055<br>0.049<br>0.907<br>0.003	0.041<br>0.002<br>0.055<br>0.060<br>0.959<br>0.003	0.170<br>0.003<br>0.059<br>0.040<br>0.830<br>0.003	0.034<br>0.003<br>0.053<br>0.056<br>0.966<br>0.003	0.054<br>-0.043<br>1.334<br>1.324<br>0.944<br>1.778	0.070<br>0.015<br>0.424<br>0.409<br>0.930<br>0.180	0.229<br>0.005<br>0.109<br>0.059<br>0.771<br>0.012	0.041<br>0.003<br>0.041<br>0.047<br>0.959<br>0.002	0.152<br>0.003<br>0.055<br>NA<br>0.889<br>0.003
	0.2	0.933<br>0.197<br>0.058<br>0.051<br>0.905<br>0.003	0.888<br>0.198<br>0.058<br>0.064<br>0.960<br>0.003	0.969<br>0.204<br>0.065<br>0.042<br>0.806<br>0.004	0.855<br>0.194<br>0.061<br>0.064<br>0.954<br>0.004	0.051<br>0.113<br>1.332<br>1.323<br>0.944<br>1.780	0.099<br>0.213<br>0.424<br>0.408<br>0.930<br>0.180	0.810<br>0.199<br>0.114<br>0.062<br>0.785<br>0.013	0.781<br>0.126<br>0.042<br>0.047<br>0.616<br>0.007	0.977<br>0.199<br>0.059<br>NA<br>0.889<br>0.004

Table T. In each cell, from top to bottom are empirical type-I error/power, mean($\hat{\theta}$), SD($\hat{\theta}$), mean(SE($\hat{\theta}$)), coverage rate, MSE, when $n = 50\,000$, p_invalid=1

m	θ	mixIE-MA	mixIE-MA-DP	cML-MA	cML-MA-DP	Egger	IVW	median	MRMix	ContMix
10	0.0	0.263	0.176	0.793	0.601	0.077	0.100	0.889	0.853	0.934
		-0.002	0.002	-0.032	-0.030	-0.003	-0.012	-0.032	-0.027	-0.024
		1.695	2.052	0.461	0.392	2.533	0.554	0.673	0.549	1.502
		1.096	1.589	0.044	0.097	2.105	0.503	0.055	0.058	NA
		0.737	0.824	0.207	0.399	0.872	0.900	0.111	0.147	0.220
		2.869	4.207	0.214	0.154	6.408	0.306	0.454	0.302	2.254
	0.2	0.265	0.175	0.771	0.542	0.075	0.114	0.872	0.844	0.924
		0.149	0.169	0.008	-0.001	0.191	0.193	0.173	-0.007	0.250
		1.684	2.055	0.459	0.389	2.523	0.550	0.670	0.559	1.527
		1.107	1.604	0.047	0.105	2.101	0.502	0.058	0.100	NA
		0.738	0.820	0.153	0.319	0.873	0.902	0.120	0.121	0.214
		2.835	4.220	0.248	0.191	6.357	0.303	0.450	0.355	2.333
30	0.0	0.573	0.244	0.662	0.295	0.072	0.054	0.839	0.876	0.963
		-0.020	-0.006	-0.003	-0.007	-0.018	-0.023	-0.022	-0.012	-0.069
		1.425	1.245	0.398	0.265	1.764	0.456	0.583	0.579	1.563
		0.538	1.031	0.062	0.114	1.614	0.446	0.070	0.080	NA
		0.427	0.756	0.338	0.705	0.911	0.946	0.161	0.124	0.131
		2.028	1.549	0.159	0.070	3.107	0.209	0.340	0.336	2.444
	0.2	0.574	0.244	0.648	0.266	0.080	0.082	0.810	0.869	0.952
		0.150	0.154	0.021	0.019	0.172	0.181	0.182	0.000	0.201
		1.404	1.210	0.410	0.282	1.759	0.455	0.582	0.599	1.558
		0.529	1.027	0.066	0.119	1.611	0.445	0.073	0.075	NA
		0.424	0.761	0.219	0.434	0.912	0.946	0.171	0.117	0.137
		1.970	1.464	0.200	0.112	3.090	0.207	0.338	0.398	2.424
100	0.0	0.765	0.158	0.777	0.215	0.052	0.063	0.718	0.783	0.948
		-0.013	-0.007	0.043	0.033	-0.010	0.025	0.048	0.034	0.067
		2.487	1.815	0.763	0.434	1.531	0.488	0.636	0.670	2.164
		0.540	1.807	0.093	0.262	1.575	0.487	0.117	0.199	NA
		0.235	0.842	0.223	0.785	0.945	0.937	0.282	0.217	0.123
		6.179	3.291	0.584	0.189	2.343	0.239	0.406	0.449	4.681
	0.2	0.778	0.166	0.777	0.196	0.050	0.091	0.738	0.776	0.945
		0.256	0.137	0.086	0.059	0.147	0.223	0.242	0.032	0.321
		2.480	1.798	0.781	0.443	1.530	0.488	0.637	0.671	2.169
		0.512	1.804	0.096	0.279	1.574	0.487	0.120	0.398	NA
		0.226	0.842	0.188	0.754	0.948	0.937	0.292	0.223	0.137
		6.148	3.232	0.623	0.216	2.343	0.238	0.407	0.478	4.714

B.1.3 Directional pleiotropy, InSIDE violated

Table U. In each cell, from top to bottom are empirical type-I error/power, mean($\hat{\theta}$), SD($\hat{\theta}$), mean(SE($\hat{\theta}$)), coverage rate, MSE, when $n = 50\,000$, $m = 10$, p_invalid=0.3

θ	b	mixIE-MA	mixIE-MA-DP	cML-MA	cML-MA-DP	Egger	IVW	median	MRMix	ContMix
0.0	0.1	0.082	0.068	0.075	0.058	0.122	0.176	0.185	0.089	0.081
		0.007	0.008	-0.001	-0.001	0.415	0.206	0.029	-0.001	-0.001
		0.099	0.081	0.021	0.020	0.944	0.222	0.097	0.027	0.023
		0.028	0.045	0.019	0.020	0.776	0.191	0.027	0.026	NA
		0.918	0.932	0.925	0.942	0.843	0.824	0.815	0.911	0.902
		0.010	0.007	0.000	0.000	1.062	0.092	0.010	0.001	0.001
	0.4	0.088	0.083	0.074	0.047	0.598	0.690	0.681	0.097	0.080
		0.012	0.036	0.000	0.000	1.028	0.426	0.285	0.000	0.000
		0.083	0.133	0.021	0.020	0.668	0.238	0.308	0.027	0.023
		0.028	0.062	0.019	0.020	0.425	0.176	0.032	0.025	NA
		0.912	0.917	0.926	0.953	0.328	0.310	0.319	0.903	0.907
		0.007	0.019	0.000	0.000	1.502	0.238	0.176	0.001	0.001
	0.7	0.082	0.096	0.070	0.057	0.850	0.914	0.915	0.092	0.080
		0.027	0.056	0.000	0.000	1.099	0.580	0.572	0.000	0.000
		0.143	0.176	0.021	0.021	0.466	0.229	0.330	0.027	0.023
		0.037	0.080	0.020	0.020	0.266	0.158	0.029	0.026	NA
		0.918	0.904	0.930	0.943	0.109	0.086	0.085	0.908	0.909
		0.021	0.034	0.000	0.000	1.425	0.389	0.435	0.001	0.001
0.2	0.1	0.986	0.918	0.999	0.992	0.146	0.646	0.998	0.986	1.000
		0.198	0.205	0.198	0.194	0.610	0.406	0.231	0.182	0.200
		0.085	0.081	0.025	0.026	0.942	0.222	0.099	0.027	0.029
		0.027	0.046	0.021	0.025	0.776	0.191	0.031	0.028	NA
		0.911	0.926	0.911	0.932	0.845	0.828	0.809	0.860	0.912
		0.007	0.007	0.001	0.001	1.055	0.092	0.011	0.001	0.001
	0.4	0.970	0.878	0.997	0.987	0.697	0.916	0.997	0.994	1.000
		0.213	0.232	0.198	0.195	1.227	0.627	0.488	0.185	0.202
		0.092	0.134	0.028	0.029	0.668	0.238	0.306	0.029	0.034
		0.033	0.063	0.022	0.025	0.425	0.176	0.036	0.025	NA
		0.907	0.916	0.906	0.923	0.328	0.312	0.323	0.852	0.901
		0.009	0.019	0.001	0.001	1.499	0.239	0.177	0.001	0.001
	0.7	0.959	0.810	0.998	0.991	0.912	0.981	0.998	0.997	1.000
		0.230	0.265	0.198	0.195	1.299	0.781	0.774	0.185	0.202
		0.149	0.195	0.028	0.030	0.466	0.229	0.329	0.029	0.051
		0.043	0.088	0.022	0.025	0.266	0.157	0.033	0.026	NA
		0.910	0.898	0.908	0.922	0.108	0.087	0.090	0.865	0.908
		0.023	0.042	0.001	0.001	1.424	0.390	0.437	0.001	0.003

Table V. In each cell, from top to bottom are empirical type-I error/power, mean($\hat{\theta}$), SD($\hat{\theta}$), mean(SE($\hat{\theta}$)), coverage rate, MSE, when $n = 50\,000$, $m = 10$, p_invalid=0.5

θ	b	mixIE-MA	mixIE-MA-DP	cML-MA	cML-MA-DP	Egger	IVW	median	MRMix	ContMix
0.0	0.1	0.073<br>0.015<br>0.174<br>0.041<br>0.927<br>0.030	0.051<br>0.033<br>0.203<br>0.085<br>0.949<br>0.042	0.100<br>0.000<br>0.026<br>0.023<br>0.900<br>0.001	0.057<br>0.000<br>0.024<br>0.024<br>0.943<br>0.001	0.090<br>0.428<br>1.104<br>0.968<br>0.861<br>1.400	0.288<br>0.310<br>0.270<br>0.236<br>0.712<br>0.169	0.452<br>0.131<br>0.233<br>0.034<br>0.548<br>0.071	0.111<br>0.000<br>0.032<br>0.029<br>0.889<br>0.001	0.107<br>0.002<br>0.059<br>NA<br>0.919<br>0.004
	0.4	0.098<br>0.038<br>0.154<br>0.057<br>0.902<br>0.025	0.085<br>0.082<br>0.234<br>0.116<br>0.915<br>0.062	0.108<br>0.002<br>0.028<br>0.023<br>0.892<br>0.001	0.067<br>0.001<br>0.026<br>0.024<br>0.933<br>0.001	0.497<br>1.038<br>0.569<br>0.482<br>0.402<br>1.401	0.801<br>0.546<br>0.237<br>0.189<br>0.199<br>0.355	0.904<br>0.492<br>0.321<br>0.031<br>0.096<br>0.345	0.150<br>0.002<br>0.036<br>0.027<br>0.850<br>0.001	0.130<br>0.012<br>0.101<br>NA<br>0.884<br>0.010
	0.7	0.107<br>0.089<br>0.250<br>0.087<br>0.893<br>0.070	0.134<br>0.149<br>0.321<br>0.152<br>0.866<br>0.125	0.105<br>0.002<br>0.029<br>0.024<br>0.895<br>0.001	0.072<br>0.001<br>0.027<br>0.025<br>0.928<br>0.001	0.834<br>1.090<br>0.357<br>0.295<br>0.117<br>1.314	0.957<br>0.684<br>0.200<br>0.154<br>0.043<br>0.508	0.980<br>0.706<br>0.265<br>0.026<br>0.020<br>0.569	0.122<br>0.002<br>0.035<br>0.028<br>0.878<br>0.001	0.121<br>0.032<br>0.162<br>NA<br>0.905<br>0.027
0.2	0.1	0.955<br>0.213<br>0.170<br>0.045<br>0.921<br>0.029	0.837<br>0.223<br>0.196<br>0.086<br>0.947<br>0.039	0.985<br>0.194<br>0.041<br>0.026<br>0.858<br>0.002	0.924<br>0.183<br>0.044<br>0.035<br>0.899<br>0.002	0.114<br>0.623<br>1.102<br>0.967<br>0.862<br>1.392	0.607<br>0.511<br>0.270<br>0.236<br>0.717<br>0.169	0.989<br>0.333<br>0.234<br>0.038<br>0.551<br>0.072	0.978<br>0.181<br>0.034<br>0.029<br>0.841<br>0.002	0.997<br>0.206<br>0.095<br>NA<br>0.908<br>0.009
	0.4	0.918<br>0.246<br>0.176<br>0.069<br>0.896<br>0.033	0.764<br>0.282<br>0.249<br>0.120<br>0.908<br>0.069	0.989<br>0.195<br>0.044<br>0.026<br>0.818<br>0.002	0.948<br>0.188<br>0.045<br>0.032<br>0.859<br>0.002	0.612<br>1.237<br>0.568<br>0.482<br>0.404<br>1.398	0.943<br>0.747<br>0.237<br>0.189<br>0.196<br>0.355	0.997<br>0.693<br>0.320<br>0.036<br>0.099<br>0.346	0.992<br>0.186<br>0.038<br>0.027<br>0.796<br>0.002	0.998<br>0.229<br>0.161<br>NA<br>0.870<br>0.027
	0.7	0.857<br>0.304<br>0.268<br>0.100<br>0.881<br>0.082	0.678<br>0.362<br>0.335<br>0.163<br>0.871<br>0.139	0.983<br>0.194<br>0.045<br>0.027<br>0.824<br>0.002	0.944<br>0.189<br>0.046<br>0.032<br>0.848<br>0.002	0.908<br>1.289<br>0.357<br>0.295<br>0.117<br>1.312	0.994<br>0.884<br>0.200<br>0.154<br>0.042<br>0.508	0.999<br>0.907<br>0.265<br>0.030<br>0.021<br>0.569	0.993<br>0.186<br>0.037<br>0.028<br>0.825<br>0.002	0.998<br>0.270<br>0.236<br>NA<br>0.861<br>0.061

Table W. In each cell, from top to bottom are empirical type-I error/power, mean($\hat{\theta}$), SD($\hat{\theta}$), mean(SE($\hat{\theta}$)), coverage rate, MSE, when $n = 50\,000$, $m = 10$, p_invalid=0.7

θ	b	mixIE-MA	mixIE-MA-DP	cML-MA	cML-MA-DP	Egger	IVW	median	MRMix	ContMix
0.0	0.1	0.073<br>0.085<br>0.361<br>0.195<br>0.927<br>0.137	0.061<br>0.128<br>0.521<br>0.381<br>0.939<br>0.288	0.146<br>-0.001<br>0.040<br>0.029<br>0.854<br>0.002	0.096<br>-0.001<br>0.036<br>0.030<br>0.904<br>0.001	0.074<br>0.506<br>1.255<br>1.119<br>0.878<br>1.828	0.363<br>0.408<br>0.301<br>0.264<br>0.637<br>0.257	0.696<br>0.287<br>0.341<br>0.039<br>0.304<br>0.199	0.200<br>0.009<br>0.117<br>0.032<br>0.800<br>0.014	0.239<br>0.100<br>0.391<br>NA<br>0.860<br>0.162
	0.4	0.153<br>0.241<br>0.408<br>0.224<br>0.847<br>0.225	0.201<br>0.336<br>0.535<br>0.301<br>0.799<br>0.398	0.196<br>0.003<br>0.047<br>0.030<br>0.804<br>0.002	0.129<br>0.002<br>0.041<br>0.031<br>0.871<br>0.002	0.431<br>1.059<br>0.572<br>0.536<br>0.449<br>1.448	0.873<br>0.636<br>0.228<br>0.191<br>0.127<br>0.457	0.981<br>0.628<br>0.304<br>0.031<br>0.019<br>0.487	0.253<br>0.022<br>0.130<br>0.031<br>0.747<br>0.017	0.401<br>0.277<br>0.485<br>NA<br>0.766<br>0.312
	0.7	0.294<br>0.418<br>0.468<br>0.259<br>0.706<br>0.394	0.339<br>0.524<br>0.541<br>0.311<br>0.661<br>0.566	0.167<br>0.003<br>0.057<br>0.032<br>0.833<br>0.003	0.130<br>0.001<br>0.044<br>0.033<br>0.870<br>0.002	0.810<br>1.091<br>0.342<br>0.325<br>0.129<br>1.308	0.985<br>0.751<br>0.179<br>0.147<br>0.015<br>0.597	0.996<br>0.776<br>0.230<br>0.024<br>0.004<br>0.655	0.234<br>0.031<br>0.157<br>0.033<br>0.766<br>0.026	0.477<br>0.462<br>0.519<br>NA<br>0.702<br>0.482
0.2	0.1	0.518<br>0.286<br>0.379<br>0.218<br>0.932<br>0.151	0.372<br>0.325<br>0.552<br>0.396<br>0.934<br>0.320	0.912<br>0.169<br>0.081<br>0.032<br>0.686<br>0.007	0.724<br>0.153<br>0.073<br>0.049<br>0.763<br>0.008	0.094<br>0.703<br>1.253<br>1.118<br>0.877<br>1.821	0.644<br>0.609<br>0.300<br>0.264<br>0.636<br>0.257	0.980<br>0.488<br>0.340<br>0.044<br>0.309<br>0.199	0.971<br>0.192<br>0.113<br>0.032<br>0.730<br>0.013	0.980<br>0.351<br>0.474<br>NA<br>0.841<br>0.247
	0.4	0.600<br>0.463<br>0.430<br>0.238<br>0.829<br>0.254	0.503<br>0.544<br>0.547<br>0.306<br>0.799<br>0.418	0.911<br>0.177<br>0.079<br>0.033<br>0.662<br>0.007	0.817<br>0.167<br>0.076<br>0.042<br>0.709<br>0.007	0.562<br>1.258<br>0.571<br>0.536<br>0.455<br>1.446	0.967<br>0.836<br>0.228<br>0.191<br>0.126<br>0.457	0.997<br>0.828<br>0.304<br>0.035<br>0.026<br>0.487	0.974<br>0.202<br>0.119<br>0.031<br>0.672<br>0.014	0.985<br>0.573<br>0.537<br>NA<br>0.706<br>0.427
	0.7	0.594<br>0.635<br>0.475<br>0.266<br>0.699<br>0.415	0.521<br>0.736<br>0.542<br>0.317<br>0.659<br>0.581	0.932<br>0.188<br>0.083<br>0.036<br>0.729<br>0.007	0.876<br>0.179<br>0.070<br>0.042<br>0.759<br>0.005	0.894<br>1.291<br>0.342<br>0.325<br>0.127<br>1.306	0.999<br>0.952<br>0.178<br>0.147<br>0.014<br>0.597	1.000<br>0.976<br>0.230<br>0.027<br>0.005<br>0.655	0.977<br>0.214<br>0.143<br>0.033<br>0.703<br>0.021	0.992<br>0.769<br>0.523<br>NA<br>0.611<br>0.597

Table X. In each cell, from top to bottom are empirical type-I error/power, mean($\hat{\theta}$), SD($\hat{\theta}$), mean(SE($\hat{\theta}$)), coverage rate, MSE, when $n = 50\,000$, $m = 30$, p_invalid=0.3

θ	b	mixIE-MA	mixIE-MA-DP	cML-MA	cML-MA-DP	Egger	IVW	median	MRMix	ContMix
0.0	0.1	0.077	0.057	0.073	0.037	0.244	0.476	0.256	0.068	0.070
		0.001	0.001	0.001	0.001	0.662	0.310	0.032	0.001	0.000
		0.018	0.019	0.018	0.017	0.694	0.197	0.041	0.028	0.019
		0.017	0.019	0.017	0.019	0.560	0.166	0.025	0.034	NA
		0.923	0.943	0.927	0.963	0.734	0.524	0.744	0.932	0.898
		0.000	0.000	0.000	0.000	0.920	0.135	0.003	0.001	0.000
	0.4	0.103	0.067	0.097	0.040	0.951	0.978	0.959	0.116	0.084
		0.001	0.003	0.001	0.001	1.169	0.605	0.516	0.004	0.000
		0.020	0.021	0.020	0.018	0.384	0.189	0.316	0.031	0.021
		0.017	0.020	0.017	0.019	0.247	0.131	0.035	0.028	NA
		0.897	0.933	0.903	0.960	0.043	0.022	0.041	0.884	0.893
		0.000	0.000	0.000	0.000	1.514	0.401	0.366	0.001	0.000
	0.7	0.090	0.095	0.080	0.036	0.999	0.999	0.998	0.115	0.064
		0.003	0.008	0.002	0.002	1.152	0.759	0.806	0.006	0.002
		0.021	0.023	0.021	0.019	0.240	0.156	0.223	0.031	0.022
		0.018	0.021	0.019	0.021	0.146	0.103	0.028	0.029	NA
		0.910	0.905	0.920	0.964	0.001	0.001	0.002	0.885	0.887
		0.000	0.001	0.000	0.000	1.384	0.601	0.699	0.001	0.001
0.2	0.1	1.000	1.000	1.000	1.000	0.335	0.861	1.000	0.983	1.000
		0.200	0.200	0.201	0.198	0.854	0.509	0.234	0.181	0.200
		0.020	0.021	0.021	0.021	0.693	0.197	0.046	0.029	0.022
		0.019	0.021	0.019	0.022	0.560	0.166	0.028	0.032	NA
		0.913	0.937	0.905	0.954	0.737	0.524	0.760	0.888	0.896
		0.000	0.000	0.000	0.000	0.907	0.134	0.003	0.001	0.000
	0.4	1.000	1.000	1.000	1.000	0.985	0.998	1.000	0.989	1.000
		0.200	0.203	0.201	0.197	1.367	0.804	0.719	0.185	0.200
		0.023	0.025	0.024	0.024	0.383	0.189	0.314	0.034	0.025
		0.019	0.023	0.019	0.023	0.247	0.131	0.040	0.030	NA
		0.888	0.910	0.889	0.950	0.045	0.021	0.048	0.832	0.868
		0.001	0.001	0.001	0.001	1.509	0.401	0.368	0.001	0.001
	0.7	1.000	0.999	1.000	0.999	0.999	1.000	1.000	0.991	1.000
		0.203	0.208	0.203	0.199	1.350	0.959	1.006	0.186	0.201
		0.024	0.027	0.025	0.025	0.240	0.156	0.224	0.034	0.027
		0.021	0.025	0.021	0.025	0.147	0.103	0.032	0.030	NA
		0.898	0.903	0.900	0.945	0.001	0.001	0.001	0.859	0.896
		0.001	0.001	0.001	0.001	1.380	0.600	0.700	0.001	0.001

Table Y. In each cell, from top to bottom are empirical type-I error/power, mean($\hat{\theta}$), SD($\hat{\theta}$), mean(SE($\hat{\theta}$)), coverage rate, MSE, when $n = 50\,000$, $m = 30$, p_invalid=0.5

θ	b	mixIE-MA	mixIE-MA-DP	cML-MA	cML-MA-DP	Egger	IVW	median	MRMix	ContMix
0.0	0.1	0.097	0.063	0.103	0.049	0.178	0.609	0.578	0.083	0.109
		0.000	0.001	0.001	0.001	0.720	0.453	0.141	0.001	0.001
		0.025	0.026	0.025	0.023	0.742	0.228	0.199	0.032	0.026
		0.021	0.026	0.020	0.023	0.688	0.200	0.035	0.036	NA
		0.903	0.937	0.897	0.951	0.805	0.391	0.422	0.917	0.880
		0.001	0.001	0.001	0.001	1.067	0.258	0.059	0.001	0.001
	0.4	0.151	0.107	0.162	0.070	0.955	0.994	0.998	0.185	0.140
		0.002	0.008	0.003	0.002	1.164	0.726	0.703	0.004	0.002
		0.029	0.040	0.029	0.026	0.327	0.173	0.252	0.040	0.031
		0.022	0.029	0.021	0.024	0.278	0.136	0.033	0.032	NA
		0.849	0.893	0.838	0.930	0.037	0.006	0.002	0.815	0.847
		0.001	0.002	0.001	0.001	1.462	0.557	0.557	0.002	0.001
	0.7	0.149	0.147	0.145	0.063	1.000	1.000	1.000	0.164	0.131
		0.006	0.021	0.003	0.003	1.135	0.837	0.863	0.005	0.004
		0.031	0.061	0.031	0.028	0.196	0.130	0.174	0.040	0.058
		0.024	0.035	0.024	0.026	0.164	0.099	0.026	0.032	NA
		0.851	0.853	0.855	0.937	0.000	0.000	0.000	0.836	0.860
		0.001	0.004	0.001	0.001	1.327	0.718	0.775	0.002	0.003
0.2	0.1	1.000	1.000	1.000	0.998	0.249	0.893	0.999	0.967	1.000
		0.199	0.201	0.201	0.195	0.912	0.653	0.345	0.178	0.200
		0.028	0.030	0.030	0.030	0.740	0.228	0.199	0.033	0.030
		0.023	0.029	0.022	0.030	0.687	0.200	0.039	0.035	NA
		0.902	0.944	0.878	0.938	0.808	0.391	0.453	0.840	0.881
		0.001	0.001	0.001	0.001	1.054	0.257	0.061	0.002	0.001
	0.4	1.000	0.997	1.000	0.984	0.981	1.000	1.000	0.980	1.000
		0.202	0.208	0.200	0.191	1.362	0.925	0.902	0.181	0.201
		0.034	0.039	0.038	0.038	0.326	0.173	0.252	0.042	0.037
		0.025	0.033	0.024	0.033	0.278	0.136	0.037	0.035	NA
		0.831	0.877	0.798	0.878	0.037	0.005	0.004	0.744	0.841
		0.001	0.002	0.001	0.001	1.457	0.556	0.557	0.002	0.001
	0.7	1.000	0.982	0.999	0.981	1.000	1.000	1.000	0.985	1.000
		0.206	0.224	0.202	0.193	1.333	1.037	1.062	0.184	0.208
		0.036	0.058	0.040	0.039	0.197	0.130	0.174	0.044	0.090
		0.027	0.040	0.027	0.035	0.164	0.099	0.029	0.033	NA
		0.845	0.851	0.813	0.891	0.000	0.000	0.000	0.779	0.844
		0.001	0.004	0.002	0.002	1.323	0.717	0.773	0.002	0.008

Table Z. In each cell, from top to bottom are empirical type-I error/power, mean($\hat{\theta}$), SD($\hat{\theta}$), mean(SE($\hat{\theta}$)), coverage rate, MSE, when $n = 50\,000$, $m = 30$, p_invalid=0.7

θ	b	mixIE-MA	mixIE-MA-DP	cML-MA	cML-MA-DP	Egger	IVW	median	MRMix	ContMix
0.0	0.1	0.137	0.075	0.205	0.068	0.141	0.709	0.817	0.146	0.206
		0.001	0.003	0.003	0.003	0.677	0.571	0.355	0.001	0.003
		0.039	0.043	0.040	0.034	0.830	0.238	0.294	0.044	0.093
		0.029	0.043	0.026	0.032	0.792	0.222	0.044	0.040	NA
		0.863	0.925	0.795	0.932	0.837	0.291	0.183	0.854	0.845
		0.002	0.002	0.002	0.001	1.148	0.383	0.212	0.002	0.009
	0.4	0.224	0.142	0.270	0.114	0.919	0.997	0.999	0.285	0.298
		0.011	0.030	0.008	0.007	1.120	0.796	0.787	0.008	0.038
		0.079	0.093	0.049	0.040	0.320	0.155	0.206	0.056	0.202
		0.032	0.063	0.027	0.033	0.315	0.137	0.032	0.032	NA
		0.776	0.858	0.730	0.886	0.069	0.003	0.001	0.715	0.765
		0.006	0.010	0.002	0.002	1.358	0.657	0.661	0.003	0.042
	0.7	0.233	0.194	0.253	0.130	1.000	1.000	1.000	0.266	0.352
		0.047	0.134	0.011	0.010	1.104	0.876	0.891	0.012	0.176
		0.152	0.211	0.050	0.043	0.187	0.110	0.144	0.059	0.388
		0.049	0.136	0.031	0.035	0.186	0.097	0.025	0.035	NA
		0.767	0.806	0.747	0.870	0.000	0.000	0.000	0.734	0.731
		0.025	0.062	0.003	0.002	1.253	0.780	0.814	0.004	0.181
0.2	0.1	0.989	0.944	0.986	0.874	0.210	0.928	0.999	0.931	0.994
		0.200	0.203	0.197	0.179	0.870	0.771	0.557	0.172	0.207
		0.044	0.048	0.053	0.054	0.828	0.237	0.293	0.046	0.117
		0.032	0.049	0.028	0.046	0.791	0.221	0.050	0.041	NA
		0.855	0.928	0.726	0.865	0.842	0.289	0.190	0.762	0.856
		0.002	0.002	0.003	0.003	1.134	0.382	0.213	0.003	0.014
	0.4	0.989	0.864	0.970	0.835	0.971	1.000	1.000	0.962	0.993
		0.213	0.236	0.191	0.172	1.318	0.995	0.986	0.183	0.276
		0.085	0.096	0.070	0.067	0.320	0.154	0.206	0.059	0.290
		0.035	0.072	0.029	0.045	0.315	0.137	0.036	0.033	NA
		0.753	0.853	0.605	0.746	0.068	0.002	0.001	0.650	0.725
		0.007	0.010	0.005	0.005	1.352	0.656	0.660	0.004	0.090
	0.7	0.940	0.707	0.964	0.849	1.000	1.000	1.000	0.957	0.996
		0.259	0.360	0.202	0.182	1.302	1.076	1.090	0.191	0.520
		0.157	0.223	0.079	0.074	0.187	0.110	0.144	0.068	0.489
		0.060	0.149	0.035	0.046	0.186	0.097	0.029	0.038	NA
		0.747	0.788	0.660	0.755	0.000	0.000	0.000	0.652	0.635
		0.028	0.075	0.006	0.006	1.249	0.779	0.812	0.005	0.342

Table AA. In each cell, from top to bottom are empirical type-I error/power, mean($\hat{\theta}$), SD($\hat{\theta}$), mean(SE($\hat{\theta}$)), coverage rate, MSE, when $n = 50\,000$, $m = 100$, p_invalid=0.3

θ	b	mixIE-MA	mixIE-MA-DP	cML-MA	cML-MA-DP	Egger	IVW	median	MRMix	ContMix
0.0	0.1	0.123	0.074	0.121	0.041	0.779	0.934	0.745	0.070	0.101
		0.001	0.002	0.002	0.002	1.320	0.630	0.110	0.005	0.001
		0.026	0.027	0.026	0.024	0.589	0.215	0.083	0.039	0.026
		0.021	0.024	0.020	0.025	0.429	0.165	0.034	0.047	NA
		0.877	0.926	0.879	0.959	0.216	0.066	0.255	0.930	0.854
		0.001	0.001	0.001	0.001	2.089	0.443	0.019	0.002	0.001
	0.4	0.182	0.134	0.199	0.059	1.000	1.000	1.000	0.268	0.150
		0.006	0.013	0.007	0.006	1.287	0.928	0.911	0.017	0.004
		0.031	0.036	0.033	0.029	0.226	0.151	0.228	0.053	0.036
		0.022	0.030	0.022	0.029	0.138	0.093	0.039	0.036	NA
		0.818	0.866	0.801	0.941	0.000	0.000	0.000	0.732	0.807
		0.001	0.001	0.001	0.001	1.707	0.884	0.881	0.003	0.001
	0.7	0.188	0.207	0.180	0.041	1.000	1.000	1.000	0.290	0.138
		0.011	0.032	0.007	0.006	1.182	0.987	1.007	0.018	0.005
		0.036	0.043	0.038	0.032	0.133	0.104	0.144	0.063	0.043
		0.025	0.035	0.025	0.033	0.080	0.062	0.028	0.041	NA
		0.812	0.793	0.820	0.959	0.000	0.000	0.000	0.710	0.809
		0.001	0.003	0.001	0.001	1.415	0.985	1.035	0.004	0.002
0.2	0.1	1.000	1.000	1.000	1.000	0.863	0.993	1.000	0.895	1.000
		0.197	0.199	0.203	0.199	1.499	0.826	0.314	0.167	0.197
		0.028	0.029	0.029	0.028	0.588	0.215	0.087	0.039	0.029
		0.022	0.027	0.022	0.029	0.430	0.165	0.038	0.048	NA
		0.884	0.921	0.872	0.950	0.225	0.067	0.289	0.859	0.863
		0.001	0.001	0.001	0.001	2.032	0.438	0.020	0.003	0.001
	0.4	0.999	0.999	1.000	0.996	1.000	1.000	1.000	0.948	1.000
		0.202	0.211	0.207	0.198	1.483	1.126	1.110	0.181	0.200
		0.035	0.041	0.041	0.038	0.226	0.151	0.229	0.055	0.041
		0.024	0.034	0.024	0.036	0.138	0.093	0.044	0.036	NA
		0.807	0.868	0.756	0.921	0.000	0.000	0.000	0.717	0.791
		0.001	0.002	0.002	0.001	1.698	0.881	0.881	0.003	0.002
	0.7	0.998	0.994	0.999	0.988	1.000	1.000	1.000	0.940	0.999
		0.207	0.235	0.207	0.197	1.380	1.185	1.207	0.186	0.202
		0.041	0.051	0.047	0.043	0.133	0.105	0.144	0.070	0.050
		0.029	0.042	0.029	0.041	0.080	0.062	0.032	0.038	NA
		0.830	0.809	0.784	0.928	0.000	0.000	0.000	0.649	0.807
		0.002	0.004	0.002	0.002	1.409	0.982	1.035	0.005	0.003

Table AB. In each cell, from top to bottom are empirical type-I error/power, mean($\hat{\theta}$), SD($\hat{\theta}$), mean(SE($\hat{\theta}$)), coverage rate, MSE, when $n = 50\,000$, $m = 100$, p_invalid=0.5

θ	b	mixIE-MA	mixIE-MA-DP	cML-MA	cML-MA-DP	Egger	IVW	median	MRMix	ContMix
0.0	0.1	0.121	0.061	0.174	0.035	0.783	0.991	0.944	0.081	0.115
		-0.001	0.002	0.003	0.003	1.412	0.852	0.390	0.001	0.001
		0.034	0.037	0.038	0.033	0.552	0.222	0.265	0.043	0.036
		0.028	0.037	0.026	0.035	0.500	0.187	0.055	0.049	NA
		0.879	0.939	0.826	0.965	0.209	0.009	0.056	0.919	0.856
		0.001	0.001	0.001	0.001	2.297	0.775	0.222	0.002	0.001
	0.4	0.256	0.171	0.325	0.090	1.000	1.000	1.000	0.344	0.282
		0.013	0.034	0.016	0.014	1.269	1.009	1.005	0.022	0.012
		0.047	0.055	0.054	0.043	0.182	0.125	0.183	0.068	0.058
		0.030	0.050	0.026	0.040	0.153	0.093	0.037	0.039	NA
		0.744	0.829	0.675	0.910	0.000	0.000	0.000	0.656	0.687
		0.002	0.004	0.003	0.002	1.645	1.034	1.043	0.005	0.003
	0.7	0.317	0.395	0.343	0.110	1.000	1.000	1.000	0.456	0.296
		0.039	0.111	0.028	0.022	1.166	1.022	1.031	0.045	0.019
		0.060	0.084	0.072	0.054	0.106	0.083	0.114	0.090	0.078
		0.035	0.067	0.032	0.045	0.089	0.060	0.027	0.041	NA
		0.683	0.605	0.657	0.890	0.000	0.000	0.000	0.544	0.673
		0.005	0.019	0.006	0.003	1.371	1.052	1.075	0.010	0.006
0.2	0.1	0.994	0.998	1.000	0.995	0.852	1.000	1.000	0.842	1.000
		0.195	0.198	0.204	0.197	1.595	1.049	0.595	0.152	0.196
		0.038	0.041	0.044	0.041	0.551	0.222	0.263	0.043	0.040
		0.032	0.040	0.028	0.042	0.501	0.187	0.060	0.050	NA
		0.867	0.944	0.780	0.949	0.217	0.010	0.056	0.753	0.837
		0.001	0.002	0.002	0.002	2.249	0.770	0.225	0.004	0.002
	0.4	0.992	0.955	0.990	0.887	1.000	1.000	1.000	0.886	0.990
		0.209	0.239	0.214	0.193	1.467	1.208	1.203	0.169	0.210
		0.054	0.064	0.074	0.064	0.183	0.125	0.183	0.068	0.070
		0.034	0.057	0.029	0.053	0.153	0.093	0.041	0.043	NA
		0.753	0.822	0.568	0.872	0.000	0.000	0.000	0.611	0.672
		0.003	0.006	0.006	0.004	1.637	1.031	1.040	0.006	0.005
	0.7	0.993	0.962	0.965	0.831	1.000	1.000	1.000	0.891	0.948
		0.242	0.344	0.239	0.206	1.364	1.221	1.229	0.197	0.240
		0.073	0.105	0.115	0.099	0.107	0.083	0.115	0.087	0.161
		0.040	0.084	0.037	0.058	0.089	0.061	0.030	0.046	NA
		0.676	0.587	0.528	0.771	0.000	0.000	0.000	0.597	0.654
		0.007	0.032	0.015	0.010	1.367	1.050	1.071	0.008	0.028

Table AC. In each cell, from top to bottom are empirical type-I error/power, mean($\hat{\theta}$), SD($\hat{\theta}$), mean(SE($\hat{\theta}$)), coverage rate, MSE, when $n = 50\,000$, $m = 100$, p_invalid=0.7

θ	b	mixIE-MA	mixIE-MA-DP	cML-MA	cML-MA-DP	Egger	IVW	median	MRMix	ContMix
0.0	0.1	0.187	0.058	0.317	0.053	0.583	0.998	0.990	0.120	0.257
		0.003	0.006	0.014	0.013	1.245	1.002	0.760	0.003	0.008
		0.057	0.061	0.064	0.052	0.562	0.227	0.332	0.053	0.062
		0.040	0.063	0.033	0.052	0.570	0.200	0.065	0.052	NA
		0.813	0.942	0.683	0.947	0.410	0.002	0.010	0.880	0.790
		0.003	0.004	0.004	0.003	1.866	1.056	0.687	0.003	0.004
	0.4	0.432	0.316	0.553	0.186	1.000	1.000	1.000	0.418	0.560
		0.043	0.113	0.062	0.049	1.203	1.047	1.036	0.027	0.060
		0.091	0.115	0.120	0.090	0.172	0.111	0.148	0.094	0.152
		0.042	0.089	0.030	0.055	0.174	0.093	0.035	0.047	NA
		0.568	0.684	0.447	0.814	0.000	0.000	0.000	0.582	0.461
		0.010	0.026	0.018	0.011	1.476	1.108	1.094	0.010	0.027
	0.7	0.592	0.633	0.638	0.372	1.000	1.000	1.000	0.490	0.741
		0.136	0.361	0.176	0.132	1.126	1.037	1.035	0.062	0.436
		0.141	0.189	0.227	0.189	0.100	0.072	0.093	0.120	0.467
		0.050	0.149	0.036	0.056	0.101	0.059	0.026	0.052	NA
		0.408	0.367	0.362	0.628	0.000	0.000	0.000	0.510	0.292
		0.038	0.166	0.083	0.053	1.279	1.081	1.080	0.018	0.408
0.2	0.1	0.942	0.825	0.973	0.785	0.696	1.000	1.000	0.776	0.968
		0.201	0.203	0.212	0.193	1.428	1.200	0.958	0.146	0.205
		0.063	0.067	0.081	0.071	0.562	0.227	0.330	0.055	0.069
		0.045	0.069	0.035	0.067	0.570	0.200	0.070	0.054	NA
		0.818	0.945	0.626	0.916	0.419	0.002	0.011	0.728	0.795
		0.004	0.004	0.007	0.005	1.824	1.052	0.683	0.006	0.005
	0.4	0.927	0.830	0.916	0.677	1.000	1.000	1.000	0.809	0.947
		0.249	0.343	0.277	0.223	1.400	1.245	1.234	0.178	0.311
		0.106	0.134	0.171	0.148	0.172	0.111	0.148	0.105	0.246
		0.047	0.105	0.034	0.070	0.174	0.093	0.038	0.045	NA
		0.568	0.665	0.347	0.686	0.000	0.000	0.000	0.487	0.436
		0.014	0.038	0.035	0.023	1.469	1.105	1.091	0.011	0.073
	0.7	0.955	0.927	0.926	0.798	1.000	1.000	1.000	0.811	0.983
		0.393	0.659	0.503	0.405	1.324	1.236	1.234	0.217	0.875
		0.189	0.206	0.290	0.266	0.100	0.072	0.094	0.146	0.466
		0.060	0.167	0.038	0.069	0.101	0.060	0.029	0.046	NA
		0.363	0.290	0.194	0.388	0.000	0.000	0.000	0.447	0.159
		0.073	0.253	0.176	0.113	1.274	1.079	1.078	0.022	0.672

B.1.4 Simulation with different sample sizes

Similar to scenarios (a) and (b) in our main simulation, i.e. directional pleiotropy and balanced pleiotropy under InSIDE assumption, we performed simulations with different sample sizes for the exposure and the outcome. Specifically, instead of using $n_x = n_y = n = 50000$, we let $n_x = 50000$ and varied n_y from $\{10000, 30000, 70000\}$. In the implementation of mixIE, we tried both n_x and n_y in the calculation of BIC and compared the results. And in the implementation of cML, we followed its authors' suggestion of using $\min(n_x, n_y)$. Figs. B to G compare our proposed methods with other MR methods, in which mixIE-MA-DP-nx and mixIE-MA-nx used n_x while mixIE-MA-DP-ny and mixIE-MA-ny used n_y in the calculation of BIC. We can see that they gave similar results and thus in the main text we used the sample size of the outcome by default.

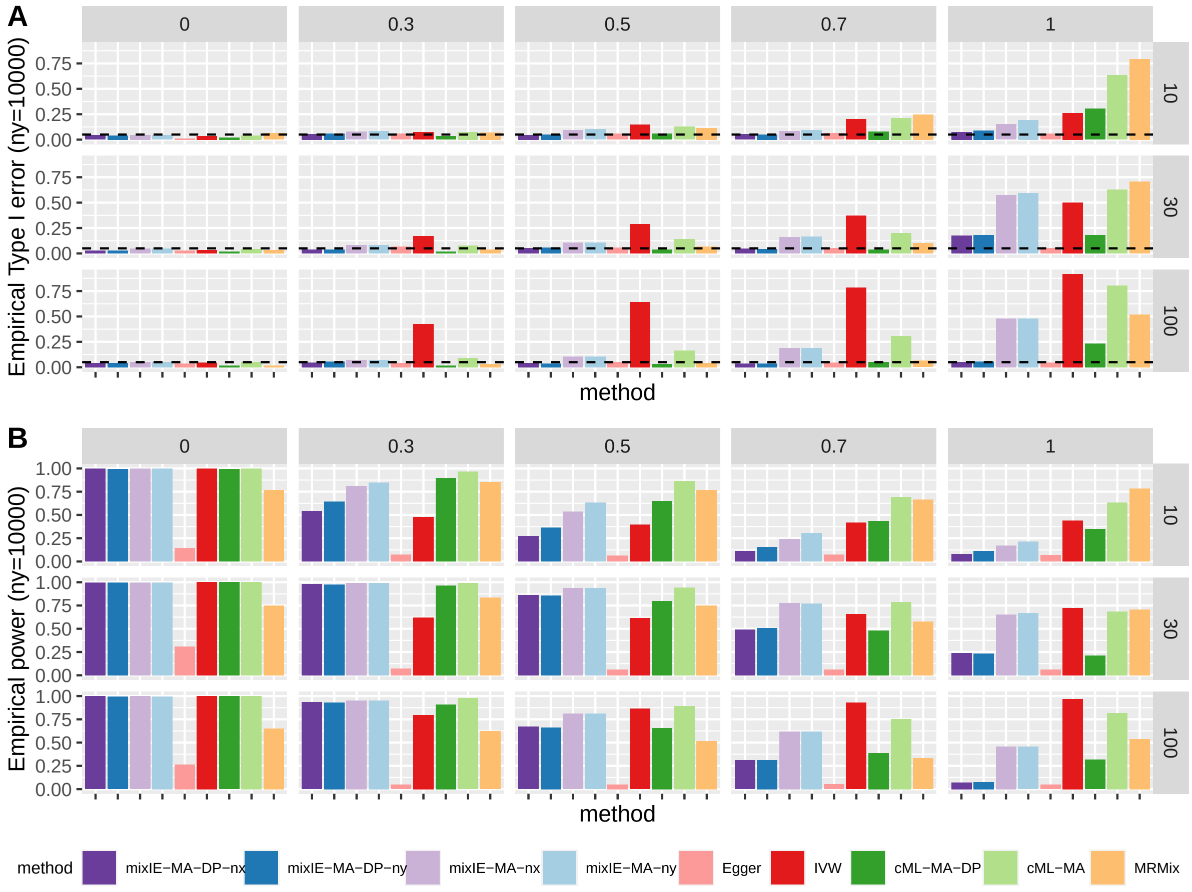

Fig B. Directional pleiotropy, InSIDE satisfied: Empirical type-I error (Panel A) or power (Panel B) with sample size $n_x = 50\,000, n_y = 10\,000$. Each row corresponds to $m = 10, 30, 100$ SNPs and each column corresponds to 0, 30%, 50%, 70%, 100% invalid IVs.

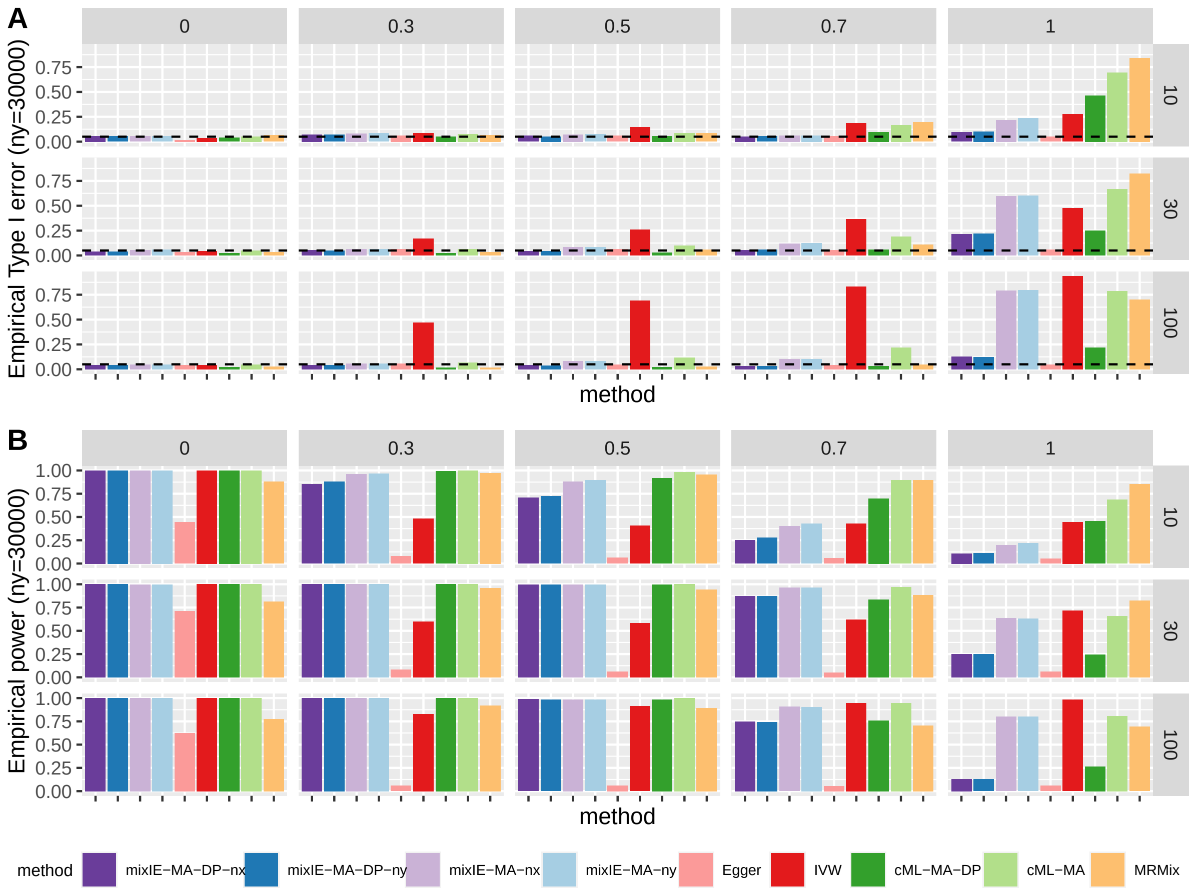

Fig C. Directional pleiotropy, InSIDE satisfied: Empirical type-I error (Panel A) or power (Panel B) with sample size $n_x = 50\,000, n_y = 30\,000$. Each row corresponds to $m = 10, 30, 100$ SNPs and each column corresponds to 0, 30%, 50%, 70%, 100% invalid IVs.

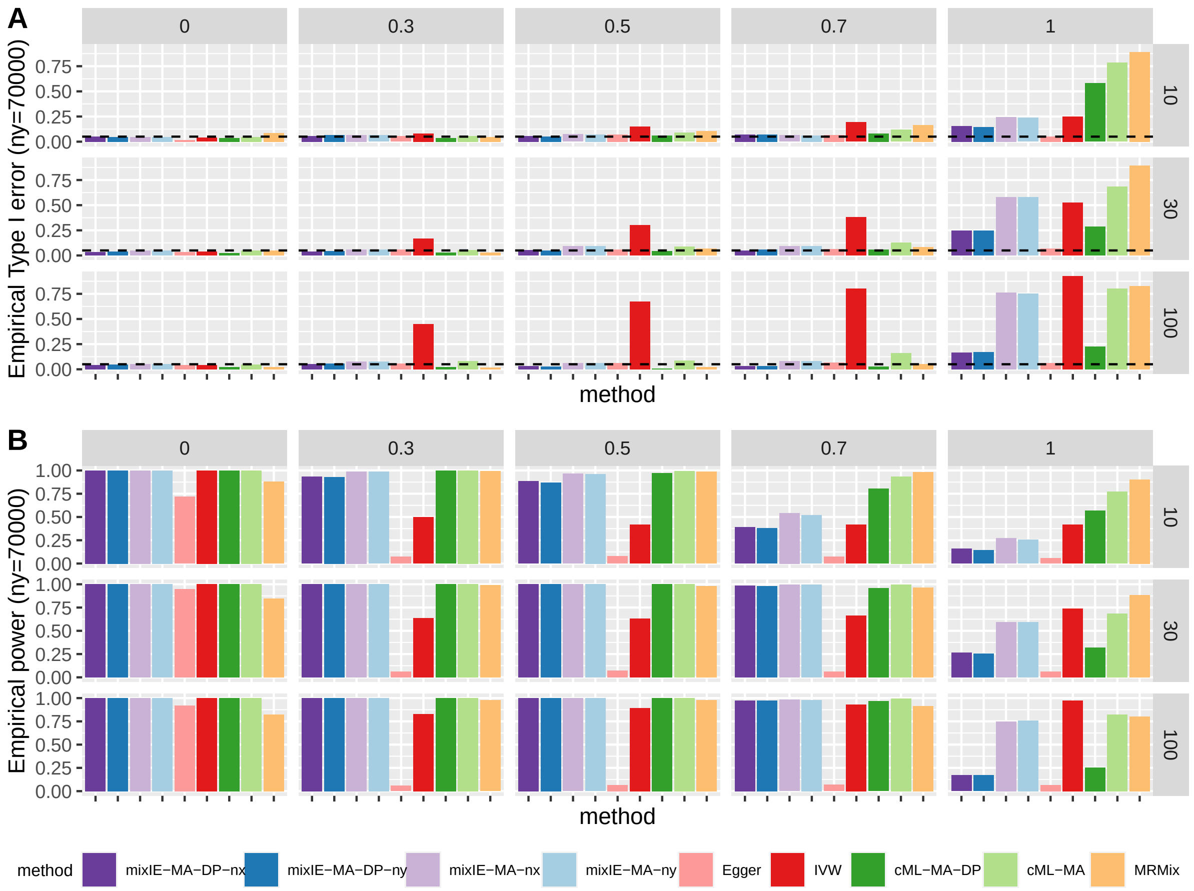

Fig D. Directional pleiotropy, InSIDE satisfied: Empirical type-I error (Panel A) or power (Panel B) with sample size $n_x = 50\,000, n_y = 70\,000$. Each row corresponds to $m = 10, 30, 100$ SNPs and each column corresponds to 0, 30%, 50%, 70%, 100% invalid IVs.

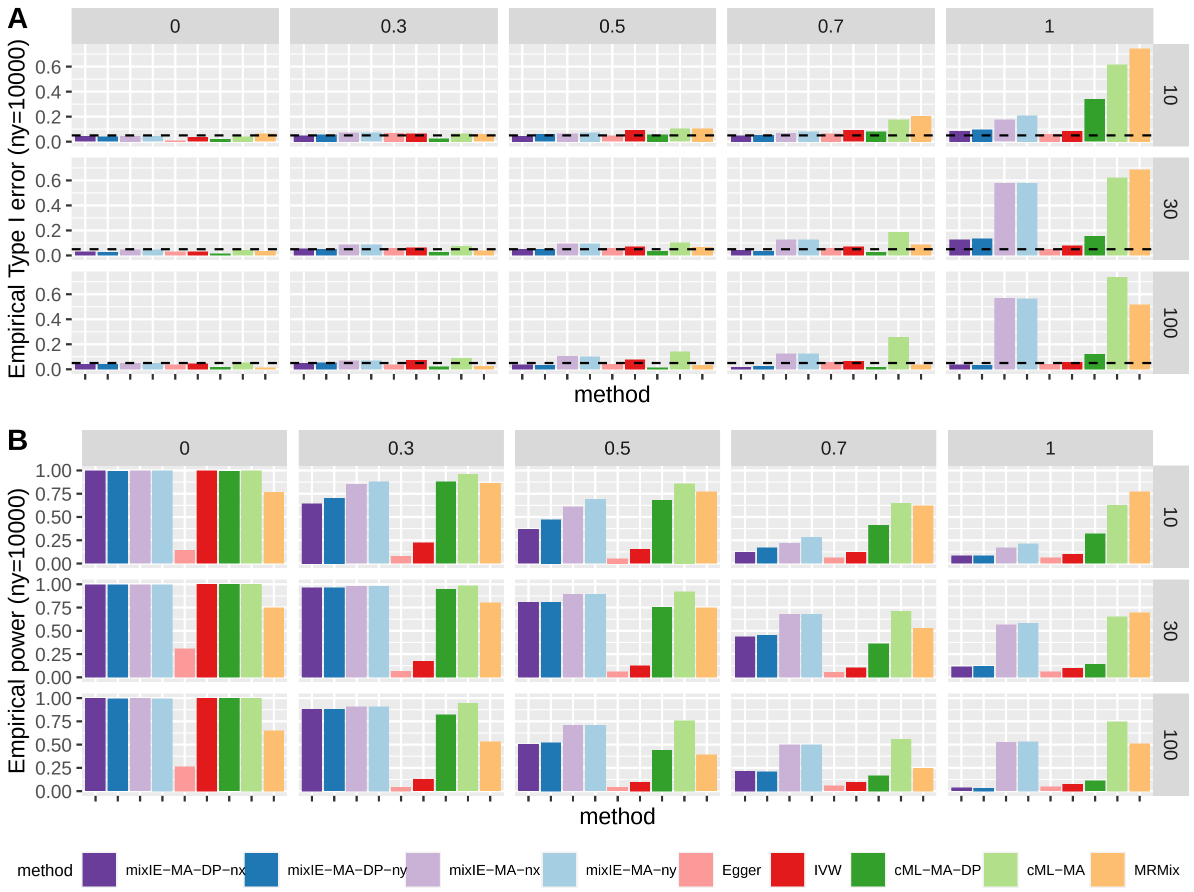

Fig E. Balanced pleiotropy, InSIDE satisfied: Empirical type-I error (Panel A) or power (Panel B) with sample size $n_x = 50\,000, n_y = 10\,000$. Each row corresponds to $m = 10, 30, 100$ SNPs and each column corresponds to 0, 30%, 50%, 70%, 100% invalid IVs.

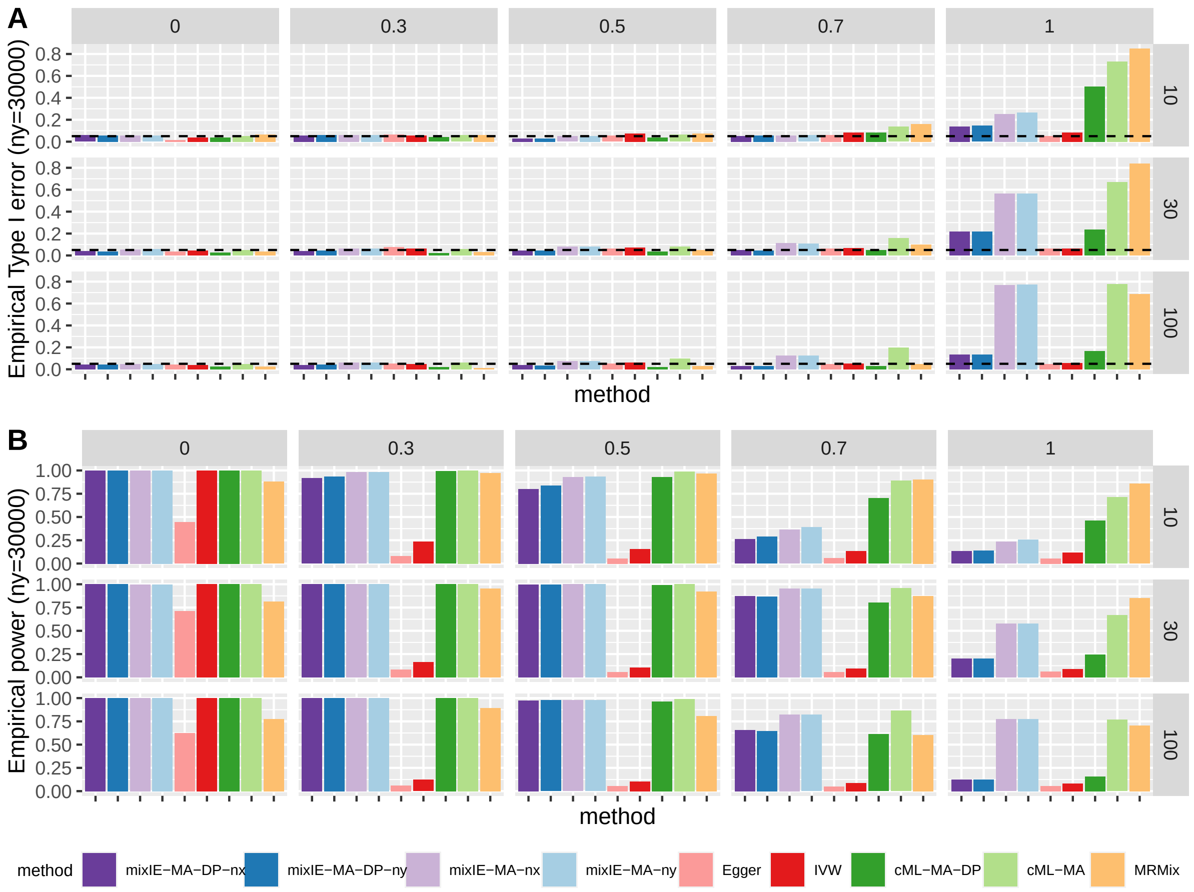

Fig F. Balanced pleiotropy, InSIDE satisfied: Empirical type-I error (Panel A) or power (Panel B) with sample size $n_x = 50\,000, n_y = 30\,000$. Each row corresponds to $m = 10, 30, 100$ SNPs and each column corresponds to 0, 30%, 50%, 70%, 100% invalid IVs.

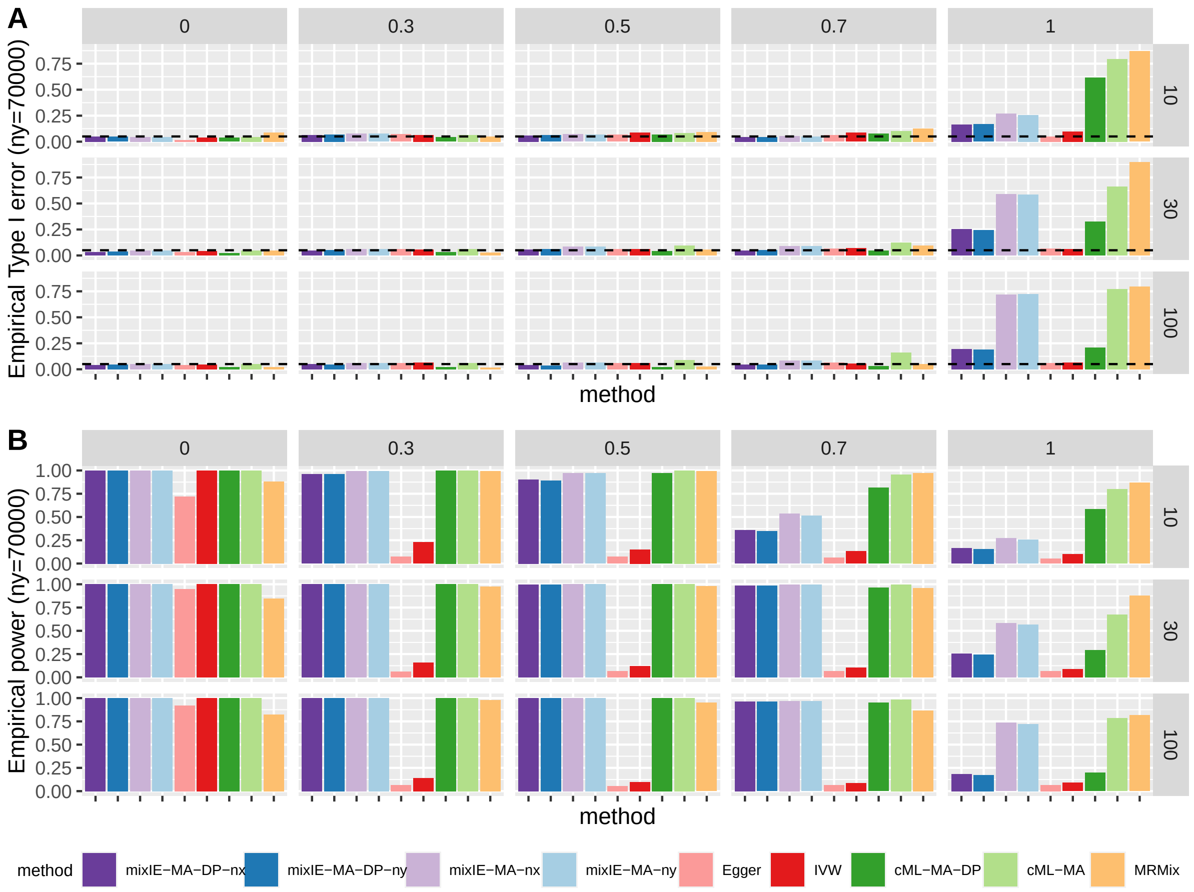

Fig G. Balanced pleiotropy, InSIDE satisfied: Empirical type-I error (Panel A) or power (Panel B) with sample size $n_x = 50\,000, n_y = 70\,000$. Each row corresponds to $m = 10, 30, 100$ SNPs and each column corresponds to 0, 30%, 50%, 70%, 100% invalid IVs.

B.2 Simulation with Weak Invalid IVs

Table AD. In each cell, from top to bottom are empirical type-I error/power, mean($\hat{\theta}$), SD($\hat{\theta}$), mean(SE($\hat{\theta}$)), coverage rate, MSE, when h_y =0.1

m	θ	mixIE-MA	mixIE-MA-DP	cML-MA	cML-MA-DP	Egger	IVW	median	MRMix	ContMix
50	-0.20	0.999	0.999	0.998	0.963	0.963	0.969	0.999	0.985	1.000
		-0.199	-0.199	-0.196	-0.182	-0.198	-0.199	-0.200	-0.201	-0.199
		0.025	0.025	0.031	0.032	0.050	0.050	0.034	0.034	0.031
		0.019	0.024	0.014	0.031	0.051	0.050	0.020	0.027	NA
		0.859	0.939	0.708	0.930	0.945	0.945	0.822	0.895	0.787
		0.001	0.001	0.001	0.001	0.003	0.003	0.001	0.001	0.001
	-0.10	0.967	0.960	0.986	0.847	0.493	0.516	0.971	0.908	0.995
		-0.099	-0.099	-0.097	-0.088	-0.099	-0.099	-0.100	-0.100	-0.099
		0.024	0.025	0.029	0.026	0.050	0.050	0.034	0.030	0.030
		0.020	0.023	0.014	0.026	0.051	0.050	0.020	0.027	NA
		0.860	0.942	0.709	0.933	0.943	0.945	0.818	0.890	0.792
		0.001	0.001	0.001	0.001	0.003	0.003	0.001	0.001	0.001
	-0.05	0.727	0.599	0.771	0.452	0.146	0.168	0.680	0.584	0.833
		-0.050	-0.050	-0.048	-0.042	-0.049	-0.050	-0.050	-0.050	-0.049
		0.025	0.025	0.028	0.023	0.050	0.050	0.034	0.030	0.030
		0.019	0.023	0.014	0.023	0.051	0.050	0.020	0.026	NA
		0.860	0.941	0.717	0.925	0.943	0.945	0.824	0.897	0.790
		0.001	0.001	0.001	0.001	0.003	0.003	0.001	0.001	0.001
	0.00	0.140	0.061	0.235	0.033	0.051	0.055	0.170	0.101	0.293
		0.000	0.000	0.000	0.000	0.001	0.000	-0.001	0.000	0.000
		0.024	0.025	0.026	0.020	0.050	0.050	0.033	0.029	0.030
		0.019	0.023	0.014	0.021	0.051	0.050	0.020	0.026	NA
		0.860	0.939	0.765	0.967	0.943	0.945	0.830	0.899	0.790
		0.001	0.001	0.001	0.000	0.003	0.003	0.001	0.001	0.001
	0.05	0.734	0.607	0.788	0.458	0.162	0.177	0.671	0.555	0.841
		0.050	0.050	0.047	0.043	0.050	0.050	0.049	0.050	0.050
		0.025	0.025	0.028	0.023	0.050	0.050	0.033	0.029	0.030
		0.020	0.023	0.014	0.023	0.051	0.050	0.020	0.027	NA
		0.863	0.941	0.727	0.922	0.942	0.944	0.830	0.899	0.788
		0.001	0.001	0.001	0.001	0.003	0.003	0.001	0.001	0.001
	0.10	0.966	0.965	0.983	0.849	0.506	0.533	0.965	0.900	0.992
		0.100	0.099	0.097	0.088	0.100	0.100	0.099	0.100	0.100
		0.025	0.025	0.029	0.027	0.050	0.050	0.034	0.030	0.030
		0.019	0.023	0.014	0.026	0.051	0.050	0.020	0.040	NA
		0.866	0.944	0.730	0.919	0.942	0.943	0.827	0.891	0.790
		0.001	0.001	0.001	0.001	0.003	0.003	0.001	0.001	0.001
	0.20	0.997	0.999	0.997	0.955	0.955	0.962	0.998	0.981	1.000
		0.199	0.199	0.196	0.182	0.200	0.199	0.198	0.200	0.200
		0.025	0.025	0.032	0.035	0.050	0.050	0.034	0.031	0.030
		0.019	0.023	0.014	0.031	0.051	0.050	0.020	0.031	NA
		0.861	0.944	0.721	0.926	0.941	0.942	0.832	0.889	0.791
		0.001	0.001	0.001	0.002	0.003	0.003	0.001	0.001	0.001

Table AE. In each cell, from top to bottom are empirical type-I error/power, mean($\hat{\theta}$), SD($\hat{\theta}$), mean(SE($\hat{\theta}$)), coverage rate, MSE, when h_y =0.2

m	θ	mixIE-MA	mixIE-MA-DP	cML-MA	cML-MA-DP	Egger	IVW	median	MRMix	ContMix
50	-0.20	0.996	0.998	0.999	0.962	0.782	0.809	0.997	0.992	1.000
		-0.199	-0.199	-0.196	-0.182	-0.198	-0.199	-0.200	-0.200	-0.199
		0.024	0.025	0.029	0.032	0.071	0.070	0.041	0.028	0.027
		0.020	0.024	0.015	0.032	0.071	0.070	0.021	0.025	NA
		0.880	0.951	0.754	0.926	0.944	0.948	0.805	0.891	0.821
		0.001	0.001	0.001	0.001	0.005	0.005	0.002	0.001	0.001
	-0.10	0.968	0.954	0.978	0.859	0.274	0.310	0.953	0.933	0.992
		-0.100	-0.099	-0.097	-0.089	-0.099	-0.099	-0.100	-0.100	-0.099
		0.024	0.024	0.027	0.026	0.071	0.070	0.041	0.027	0.027
		0.020	0.024	0.015	0.026	0.071	0.070	0.021	0.025	NA
		0.886	0.948	0.764	0.927	0.944	0.948	0.807	0.894	0.811
		0.001	0.001	0.001	0.001	0.005	0.005	0.002	0.001	0.001
	-0.05	0.748	0.584	0.788	0.462	0.098	0.108	0.656	0.582	0.835
		-0.050	-0.050	-0.048	-0.043	-0.049	-0.050	-0.050	-0.050	-0.050
		0.024	0.024	0.025	0.022	0.071	0.070	0.041	0.027	0.027
		0.019	0.024	0.015	0.023	0.071	0.070	0.021	0.025	NA
		0.882	0.948	0.768	0.923	0.943	0.947	0.807	0.892	0.815
		0.001	0.001	0.001	0.001	0.005	0.005	0.002	0.001	0.001
	0.00	0.114	0.052	0.186	0.040	0.048	0.053	0.182	0.108	0.285
		0.000	0.000	0.000	0.000	0.001	0.000	-0.001	0.000	0.000
		0.024	0.024	0.024	0.020	0.071	0.070	0.041	0.027	0.027
		0.020	0.024	0.015	0.020	0.071	0.070	0.021	0.026	NA
		0.886	0.948	0.814	0.960	0.944	0.947	0.818	0.892	0.805
		0.001	0.001	0.001	0.000	0.005	0.005	0.002	0.001	0.001
	0.05	0.734	0.603	0.809	0.476	0.097	0.111	0.661	0.589	0.849
		0.050	0.050	0.048	0.043	0.051	0.050	0.049	0.050	0.050
		0.024	0.024	0.025	0.022	0.071	0.070	0.041	0.027	0.027
		0.020	0.024	0.015	0.023	0.071	0.070	0.021	0.025	NA
		0.885	0.946	0.773	0.929	0.944	0.947	0.817	0.894	0.818
		0.001	0.001	0.001	0.001	0.005	0.005	0.002	0.001	0.001
	0.10	0.976	0.961	0.988	0.869	0.293	0.308	0.947	0.910	0.995
		0.100	0.100	0.098	0.090	0.100	0.100	0.099	0.100	0.100
		0.024	0.024	0.026	0.026	0.071	0.070	0.041	0.027	0.027
		0.019	0.024	0.015	0.026	0.071	0.070	0.021	0.026	NA
		0.881	0.946	0.778	0.926	0.944	0.947	0.818	0.890	0.810
		0.001	0.001	0.001	0.001	0.005	0.005	0.002	0.001	0.001
	0.20	0.994	0.999	0.996	0.950	0.792	0.805	0.994	0.995	1.000
		0.199	0.199	0.196	0.182	0.200	0.199	0.198	0.201	0.199
		0.024	0.025	0.030	0.034	0.071	0.071	0.041	0.028	0.027
		0.020	0.024	0.015	0.032	0.071	0.070	0.021	0.029	NA
		0.882	0.942	0.764	0.929	0.944	0.946	0.816	0.890	0.817
		0.001	0.001	0.001	0.001	0.005	0.005	0.002	0.001	0.001

Table AF. In each cell, from top to bottom are empirical type-I error/power, mean($\hat{\theta}$), SD($\hat{\theta}$), mean(SE($\hat{\theta}$)), coverage rate, MSE, when h_y =0.4

m	θ	mixIE-MA	mixIE-MA-DP	cML-MA	cML-MA-DP	Egger	IVW	median	MRMix	ContMix
50	-0.20	0.997<br>-0.199<br>0.024<br>0.020<br>0.897<br>0.001	0.998<br>-0.199<br>0.024<br>0.024<br>0.950<br>0.001	0.999<br>-0.197<br>0.028<br>0.016<br>0.803<br>0.001	0.958<br>-0.184<br>0.032<br>0.030<br>0.943<br>0.001	0.497<br>-0.198<br>0.100<br>0.099<br>0.942<br>0.010	0.526<br>-0.199<br>0.099<br>0.098<br>0.945<br>0.010	0.992<br>-0.200<br>0.051<br>0.022<br>0.798<br>0.003	0.986<br>-0.199<br>0.027<br>0.026<br>0.920<br>0.001	1.000<br>-0.198<br>0.026<br>NA<br>0.838<br>0.001
	-0.10	0.973<br>-0.099<br>0.023<br>0.019<br>0.905<br>0.001	0.954<br>-0.099<br>0.024<br>0.024<br>0.955<br>0.001	0.986<br>-0.098<br>0.025<br>0.015<br>0.798<br>0.001	0.888<br>-0.090<br>0.025<br>0.025<br>0.936<br>0.001	0.155<br>-0.098<br>0.100<br>0.099<br>0.942<br>0.010	0.174<br>-0.099<br>0.099<br>0.098<br>0.947<br>0.010	0.941<br>-0.100<br>0.051<br>0.022<br>0.803<br>0.003	0.912<br>-0.099<br>0.027<br>0.025<br>0.916<br>0.001	0.991<br>-0.098<br>0.026<br>NA<br>0.830<br>0.001
	-0.05	0.733<br>-0.049<br>0.023<br>0.019<br>0.907<br>0.001	0.619<br>-0.049<br>0.024<br>0.023<br>0.956<br>0.001	0.785<br>-0.048<br>0.024<br>0.015<br>0.802<br>0.001	0.515<br>-0.044<br>0.022<br>0.022<br>0.934<br>0.001	0.073<br>-0.049<br>0.100<br>0.099<br>0.942<br>0.010	0.079<br>-0.050<br>0.099<br>0.098<br>0.947<br>0.010	0.644<br>-0.050<br>0.051<br>0.022<br>0.805<br>0.003	0.602<br>-0.049<br>0.026<br>0.026<br>0.915<br>0.001	0.835<br>-0.049<br>0.026<br>NA<br>0.831<br>0.001
	0.00	0.096<br>0.000<br>0.023<br>0.019<br>0.904<br>0.001	0.046<br>0.000<br>0.024<br>0.023<br>0.954<br>0.001	0.166<br>0.001<br>0.023<br>0.015<br>0.834<br>0.001	0.028<br>0.000<br>0.019<br>0.020<br>0.972<br>0.000	0.049<br>0.001<br>0.100<br>0.099<br>0.941<br>0.010	0.053<br>0.000<br>0.099<br>0.098<br>0.947<br>0.010	0.190<br>-0.001<br>0.052<br>0.022<br>0.810<br>0.003	0.078<br>0.001<br>0.027<br>0.026<br>0.922<br>0.001	0.246<br>0.001<br>0.026<br>NA<br>0.822<br>0.001
	0.05	0.731<br>0.050<br>0.023<br>0.020<br>0.903<br>0.001	0.621<br>0.050<br>0.024<br>0.023<br>0.952<br>0.001	0.806<br>0.049<br>0.024<br>0.015<br>0.819<br>0.001	0.527<br>0.044<br>0.022<br>0.022<br>0.931<br>0.000	0.084<br>0.051<br>0.100<br>0.099<br>0.942<br>0.010	0.088<br>0.050<br>0.099<br>0.098<br>0.946<br>0.010	0.655<br>0.049<br>0.051<br>0.022<br>0.811<br>0.003	0.598<br>0.051<br>0.027<br>0.026<br>0.918<br>0.001	0.845<br>0.051<br>0.026<br>NA<br>0.812<br>0.001
	0.10	0.979<br>0.100<br>0.023<br>0.019<br>0.901<br>0.001	0.958<br>0.100<br>0.024<br>0.024<br>0.953<br>0.001	0.989<br>0.099<br>0.025<br>0.015<br>0.809<br>0.001	0.896<br>0.091<br>0.024<br>0.025<br>0.939<br>0.001	0.167<br>0.101<br>0.100<br>0.099<br>0.941<br>0.010	0.182<br>0.099<br>0.099<br>0.098<br>0.946<br>0.010	0.935<br>0.099<br>0.051<br>0.022<br>0.813<br>0.003	0.925<br>0.101<br>0.028<br>0.025<br>0.917<br>0.001	0.996<br>0.101<br>0.025<br>NA<br>0.813<br>0.001
	0.20	0.999<br>0.199<br>0.024<br>0.019<br>0.901<br>0.001	0.999<br>0.199<br>0.024<br>0.024<br>0.950<br>0.001	0.997<br>0.198<br>0.028<br>0.016<br>0.799<br>0.001	0.972<br>0.186<br>0.031<br>0.030<br>0.937<br>0.001	0.518<br>0.200<br>0.100<br>0.099<br>0.943<br>0.010	0.537<br>0.199<br>0.099<br>0.098<br>0.946<br>0.010	0.995<br>0.198<br>0.051<br>0.022<br>0.810<br>0.003	0.990<br>0.201<br>0.028<br>0.026<br>0.915<br>0.001	1.000<br>0.200<br>0.026<br>NA<br>0.801<br>0.001

C Primary Real Data Analysis

C.1 Main Analysis

C.1.1 Example of BF-T2D

As mentioned in the main text, mixIE-MA and mixIE-MA-DP both gave significant results for the causal pair BF-T2D after bonferroni adjustment while Egger regression and IVW were only marginally significant. And mixIE-MA identified the same invalid IV as cML-MA (the red point in Fig. H).

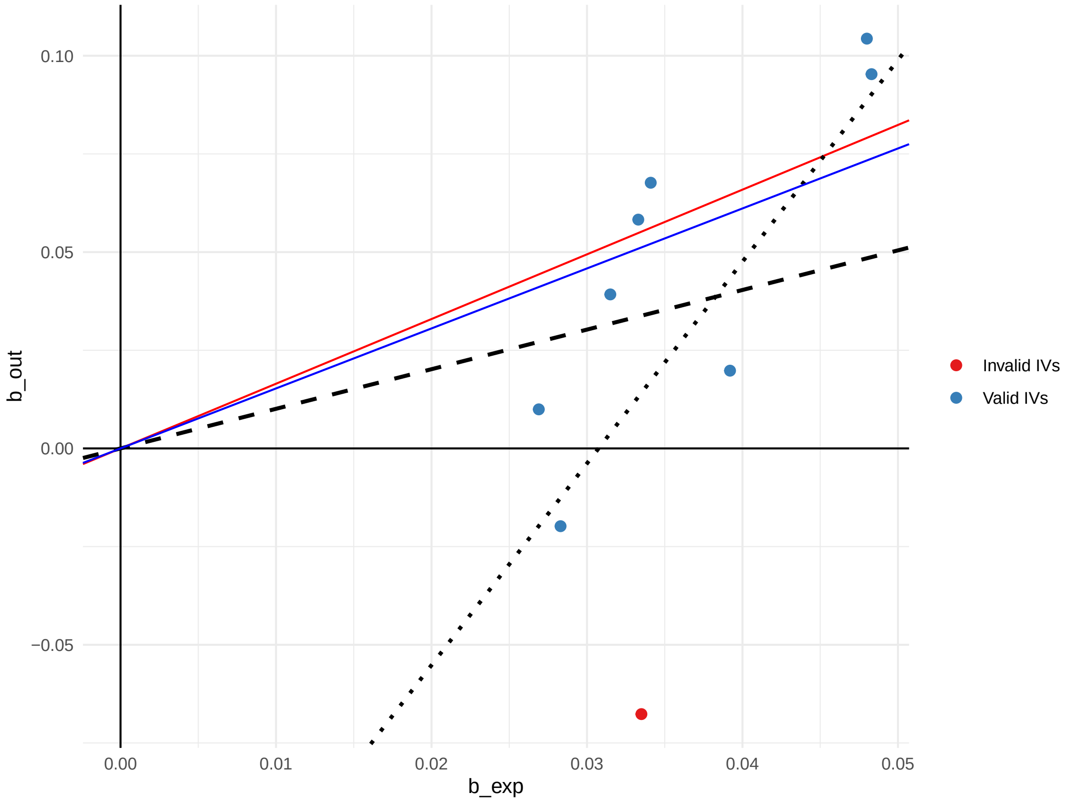

Fig H. GWAS scatter plot for BF-T2D pair. Blue points are valid IVs identified by mixIE-MA and cML-MA; red point is the invalid IV identified by mixIE-MA and cML-MA. Red solid line is the estimate by mixIE-MA; blue solid line is the estimate by cML-MA; black dashed line is the estimate by IVW; black dotted line is the estimate by Egger regression.

C.1.2 Examples of FG-T2D and TG-CAD

As mentioned in the main text, mixIE-MA and mixIE-MA-DP may give different results for some of the pairs with a high proportion of invalid IVs. For example, Figs. Ia and Ib show the details for the two pairs, FG-T2D and TG-CAD, respectively.

For the FG-T2D pair, mixIE-MA identified 6 invalid IVs while mixIE-MA-DP identified the same five invalid IVs as that by cML-MA (shown in the Supplementary of [2]). However, as shown in panel D in Fig. Ia, most of the estimates from data perturbation were close to the estimate by mixIE-MA except a few negative ones, which resulted in a large standard error and thus an insignificant result. For those perturbed datasets giving negative estimates, the Egger regression model gave negative estimates and had larger weights in model averaging, leading to those extreme and negative estimates in the histogram.

For TG-CAD pair, we can see that there was an obvious discrepancy between the sets of invalid IVs identified by mixIE-MA and by mixIE-MA-DP respectively. For example, as shown in panel C in Fig. Ib, the 112-120th IVs were identified as invalid by mixIE-MA while in mixIE-MA-DP, they were identified as invalid IVs less than 100 times out of the 200 data perturbations. Also, as shown in the Supplementary of [2], the set of invalid IVs identified by mixIE-MA-DP was closer to the one identified by cML-MA. It is more likely that mixIE-MA-DP corrected some incorrect classifications of valid versus invalid IVs by mixIE-MA, and thus gave a more reliable estimate in this example.

(a) FG-T2D

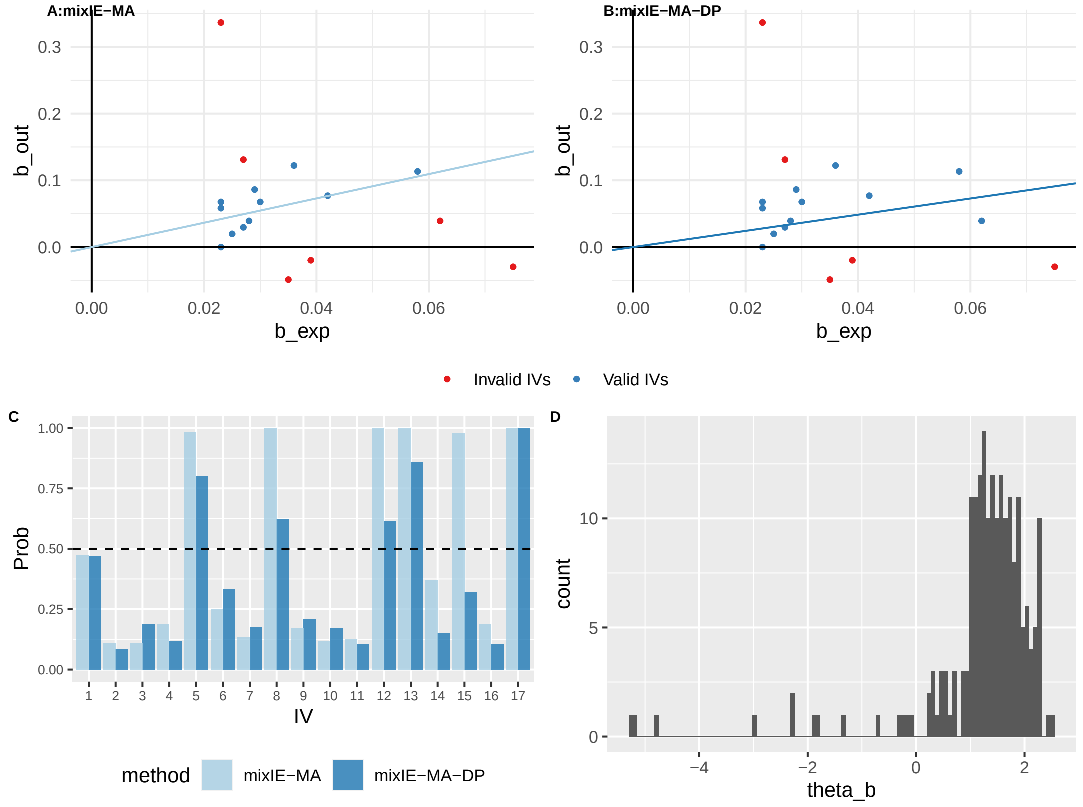

(b) TG-CAD

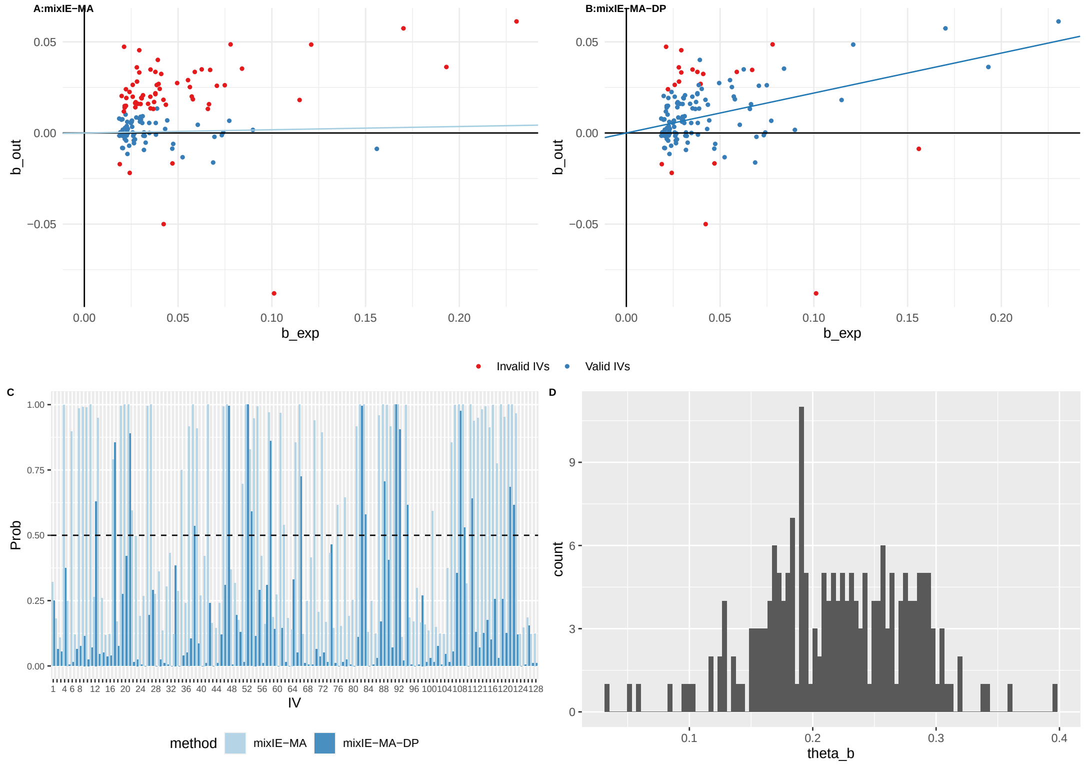

Fig I. The results of mixIE for (a) FG-T2D and (b) TG-CAD. Within each, panels A and B are estimates and identified invalid IVs by mixIE-MA and mixIE-MA-DP respectively, panel C is the posterior probability of each IV being invalid (mixIE-MA-DP used empirical frequency), and panel D is the histogram of $\hat{\theta}^{(b)*}$ from 200 data perturbations.

C.1.3 Full results for the 48 pairs of mixIE

Table AG. 48 pairs of risk factors and diseases results (***:pval<0.001, **:0.001<pval<0.05)

class	risk_disease	m	mixIE-MA-DP_est	mixIE-MA-DP_se	mixIE-MA_est	mixIE-MA_se	mixIE-MA_pi
C	BF-STROKE	10	-1.07e-02	1.01e-01	-1.31e-02	9.22e-02	0.00
	BF-CAD	10	-2.53e-02	1.50e+00	-9.40e-02	1.27e+00	1.00
	BF-T2D	9	1.40e+00***	3.88e-01	1.65e+00***	4.72e-01	0.11
	BMI-STROKE	90	7.43e-02	5.34e-02	-2.57e-02	4.09e-02	0.13
	BMI-CAD	90	3.41e-01***	3.40e-02	3.32e-01***	2.33e-02	0.09
	BMI-T2D	90	8.44e-01***	6.99e-02	8.40e-01***	7.78e-02	0.01
	DBP-STROKE	1343	5.41e-02***	1.30e-02	4.63e-02***	2.03e-03	0.01
	DBP-CAD	1345	5.31e-02***	2.07e-03	5.26e-02***	1.36e-03	0.18
	FG-T2D	17	1.21e+00	1.13e+00	1.82e+00***	3.51e-01	0.35
	HEIGHT-CAD	986	-8.76e-02***	1.67e-02	-8.30e-02***	8.01e-03	0.09
	LDL-STROKE	184	8.81e-02***	1.70e-02	6.98e-02***	1.39e-02	0.03
	LDL-CAD	184	3.79e-01***	3.07e-02	4.34e-01***	1.26e-02	0.27
	SBP-STROKE	1319	3.48e-02***	9.52e-03	2.68e-02***	1.12e-03	0.01
	SBP-CAD	1324	3.40e-02***	1.16e-03	3.14e-02***	7.72e-04	0.15
	SMOKE-STROKE	129	3.12e-01***	8.27e-02	3.13e-01***	8.24e-02	0.00
	SMOKE-ASTHMA	114	2.80e-01	1.52e-01	4.20e-01**	1.33e-01	0.04
	SMOKE-CAD	129	5.71e-01***	1.15e-01	7.54e-01***	6.45e-02	0.16
	SMOKE-T2D	115	3.18e-01	1.81e-01	3.50e-01**	1.70e-01	0.00
	TG-CAD	128	2.19e-01***	5.96e-02	1.78e-02	1.95e-02	0.49
R	ALCOHOL-STROKE	53	7.48e-02	9.31e-02	9.09e-02	9.33e-02	0.00
	ALCOHOL-CAD	54	1.69e-01	1.24e-01	2.30e-01***	6.05e-02	0.11
	ALCOHOL-T2D	44	2.53e-01	2.15e-01	2.46e-01	2.07e-01	0.00
	BF-ASTHMA	10	1.21e-01	1.48e-01	1.18e-01	1.35e-01	0.00
	BMI-ASTHMA	88	1.42e-01**	5.65e-02	1.42e-01**	5.90e-02	0.00
	BW-STROKE	65	-1.15e-01	7.91e-02	-1.10e-01	7.93e-02	0.05
	BW-CAD	65	-1.36e-01	9.54e-02	-1.17e-01***	3.37e-02	0.28
	BW-T2D	54	-4.80e-01**	1.91e-01	-3.90e-01***	1.12e-01	0.15
	DBP-T2D	1110	2.30e-02**	7.63e-03	1.78e-02***	4.09e-03	0.01
	FG-STROKE	17	7.70e-02	8.65e-02	3.75e-02	9.48e-02	0.00
	FG-CAD	17	2.37e-01***	5.19e-02	2.32e-01***	5.54e-02	0.00
	HDL-T2D	188	-9.29e-02	6.69e-02	1.45e-03	4.13e-02	0.11
	HEIGHT-STROKE	986	-2.83e-02	1.47e-02	-1.76e-02	1.26e-02	0.01
	LDL-T2D	178	-1.53e-01***	3.37e-02	-1.81e-01***	3.47e-02	0.03
	SBP-T2D	1110	2.05e-02***	2.39e-03	1.99e-02***	2.40e-03	0.01
	TG-STROKE	128	-3.64e-03	2.21e-02	-3.22e-03	2.18e-02	0.00
	TG-T2D	124	5.64e-02	7.78e-02	-7.88e-02	4.82e-02	0.10
	ALCOHOL-ASTHMA	44	-9.74e-02	1.45e-01	-8.71e-02	1.48e-01	0.00
	BW-ASTHMA	54	1.47e-01	1.12e-01	2.96e-01**	1.11e-01	0.11
	DBP-ASTHMA	1108	-5.27e-04	6.21e-03	-7.63e-04	3.09e-03	0.01
	FG-ASTHMA	17	-1.95e-01	1.49e-01	-2.67e-01**	1.12e-01	0.12

Table AG. 48 pairs of risk factors and diseases results (***:pval<0.001, **:0.001<pval<0.05) *(continued)*

class	risk_disease	m	mixIE-MA-DP_est	mixIE-MA-DP_se	mixIE-MA_est	mixIE-MA_se	mixIE-MA_pi
U	HDL-ASTHMA	188	1.95e-02	2.67e-02	9.73e-03	2.67e-02	0.02
	HEIGHT-ASTHMA	977	-1.60e-02	1.76e-02	-3.93e-03	1.98e-02	0.01
	HEIGHT-T2D	982	3.00e-02	1.36e-01	3.03e-02	9.06e-02	1.00
	LDL-ASTHMA	173	1.03e-02	2.79e-02	-6.83e-03	2.50e-02	0.02
	SBP-ASTHMA	1106	4.01e-03**	1.99e-03	3.21e-03	2.01e-03	0.01
	TG-ASTHMA	122	-8.15e-02**	3.93e-02	-8.89e-02**	3.35e-02	0.00
N	HDL-STROKE	197	-3.40e-02**	1.73e-02	-3.12e-02	1.67e-02	0.01
	HDL-CAD	197	-1.13e-01**	5.60e-02	-9.83e-02***	1.40e-02	0.47

C.2 Sensitivity Analysis

C.2.1 FG-T2D

As shown previously in Fig. Ia, where by default we included the Egger regression model *explicitly* in the candidate model list, there were a few negative estimates from data perturbation; these negative estimates resulted when the Egger regression model gave negative estimates and had larger weights in model averaging on those perturbed datasets. Thus, we performed mixIE-MA and mixIE-MA-DP without including the Egger regression model *explicitly* in the candidate model list (but it was still possible that the CEM algorithm would estimate all IVs to be invalid and thus end up with the Egger regression model). The results are shown in Fig. J: those extreme negative estimates did not appear in the histogram this time, and mixIE-MA-DP gave a nearly significant result (p-value=0.003) with a point estimate 1.48.

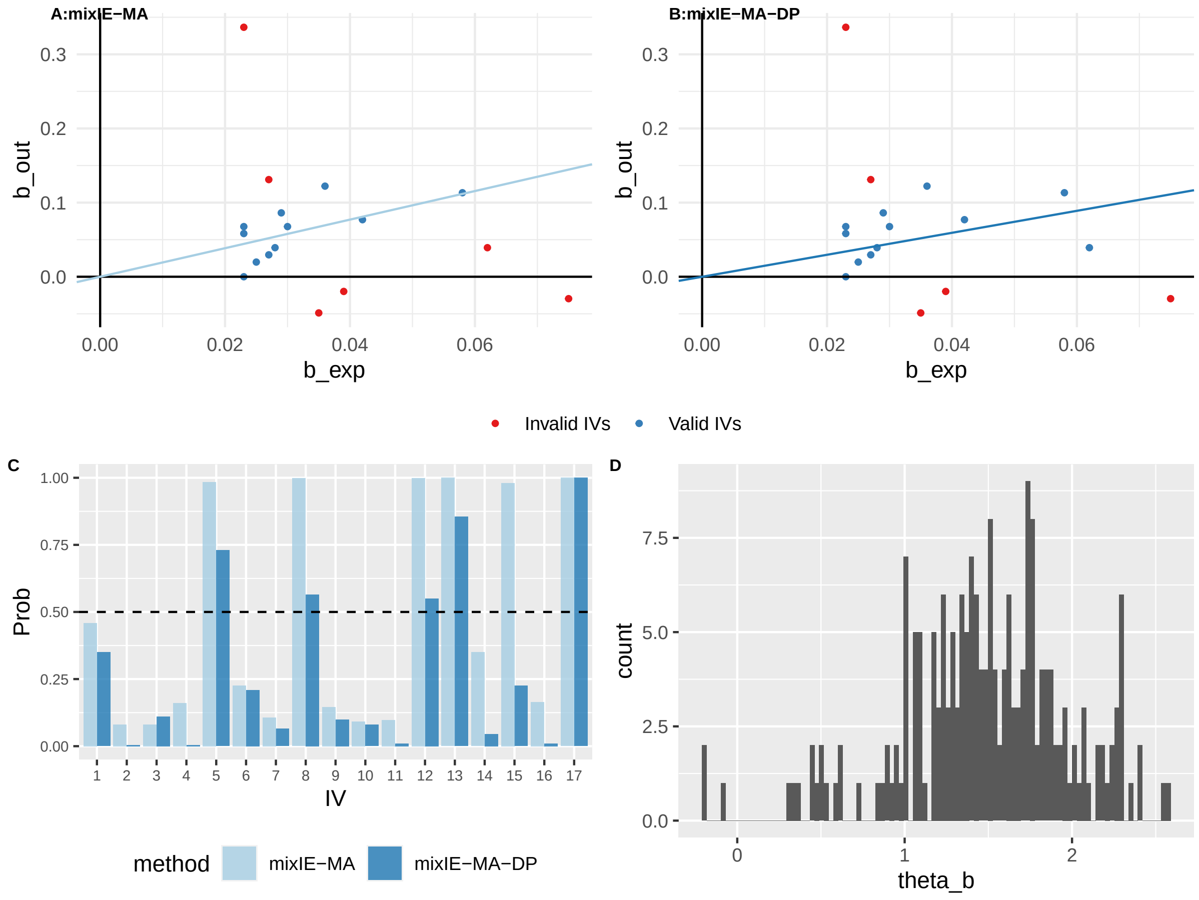

Fig J. The results for FG-T2D without including the Egger regression model explicitly in the list of the candidate models.

C.2.2 Removal of Invalid IVs

As shown in Fig. I, mixIE-MA-DP was more likely to identify the correct set of invalid IVs. We applied mixIE-MA and mixIE-MA-DP again to the 48 risk factor-disease pairs after removing the invalid IVs identified by mixIE-MA-DP. Now both methods gave significant results for the two pairs, FG-T2D and TG-CAD. Complete results are given in Fig. K.

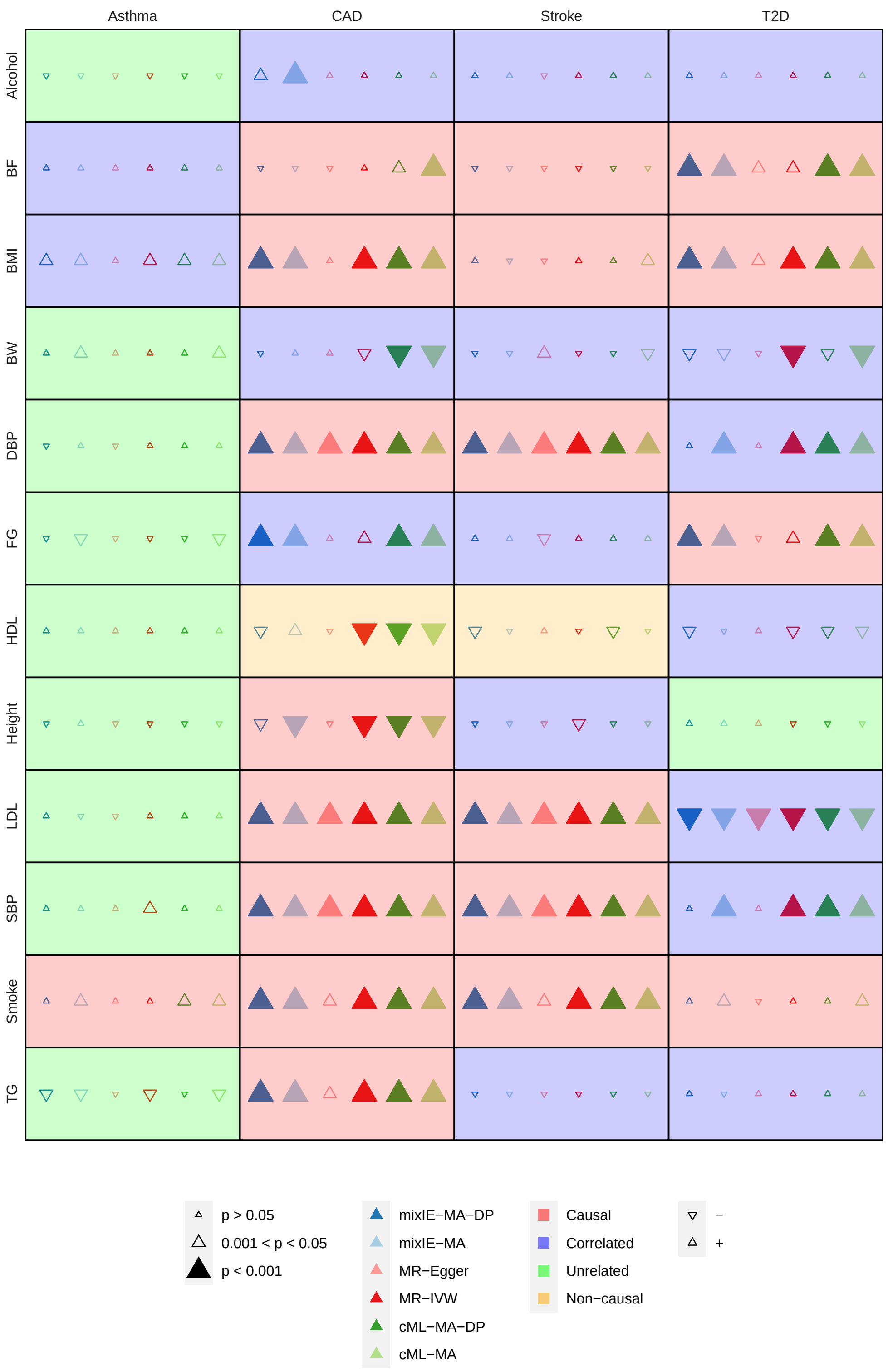

Fig K. Apply mixIE-MA and mixIE-MA-DP after removing invalid IVs identified by mixIE-MA-DP in main analysis.

C.2.3 Reorientation of IVs

One of the issues of Egger regression is that it is sensitive to the coding of SNPs (because of the intercept term in its regression model), and our proposed methods would inherit this problem from Egger regression. Here we performed the analysis using the original coding of SNPs provided in [3] for mixIE-MA, mixIE-MA-DP and Egger regression. Table AH compares the total numbers of significant pairs identified by our proposed methods and Egger regression with or without reorienting IVs respectively. It appeared that mixIE-MA performed more robustly to the coding of SNPs than Egger regression: using the original coding of IVs, Egger regression now identified 12 causal pairs but only 6 before; in contrast, mixIE-MA-DP identified one more causal pair (FG-T2D) than before and mixIE-MA identified one more causal pair (TG-CAD) but BF-T2D became marginally significant now. Complete results are given in Fig. L.

Table AH. Numbers of significant pairs among 48 risk factor-disease pairs at the significance cutoff of p-value < 0.001.

	Causal	Correlated	Unrelated	Non-causal
mixIE-MA (No reorientation)	13	6	0	1
mixIE-MA-DP (No reorientation)	14	5	0	1
Egger (No reorientation)	12	4	0	1
mixIE-MA	13	7	0	1
mixIE-MA-DP	13	3	0	0
Egger	6	1	0	0

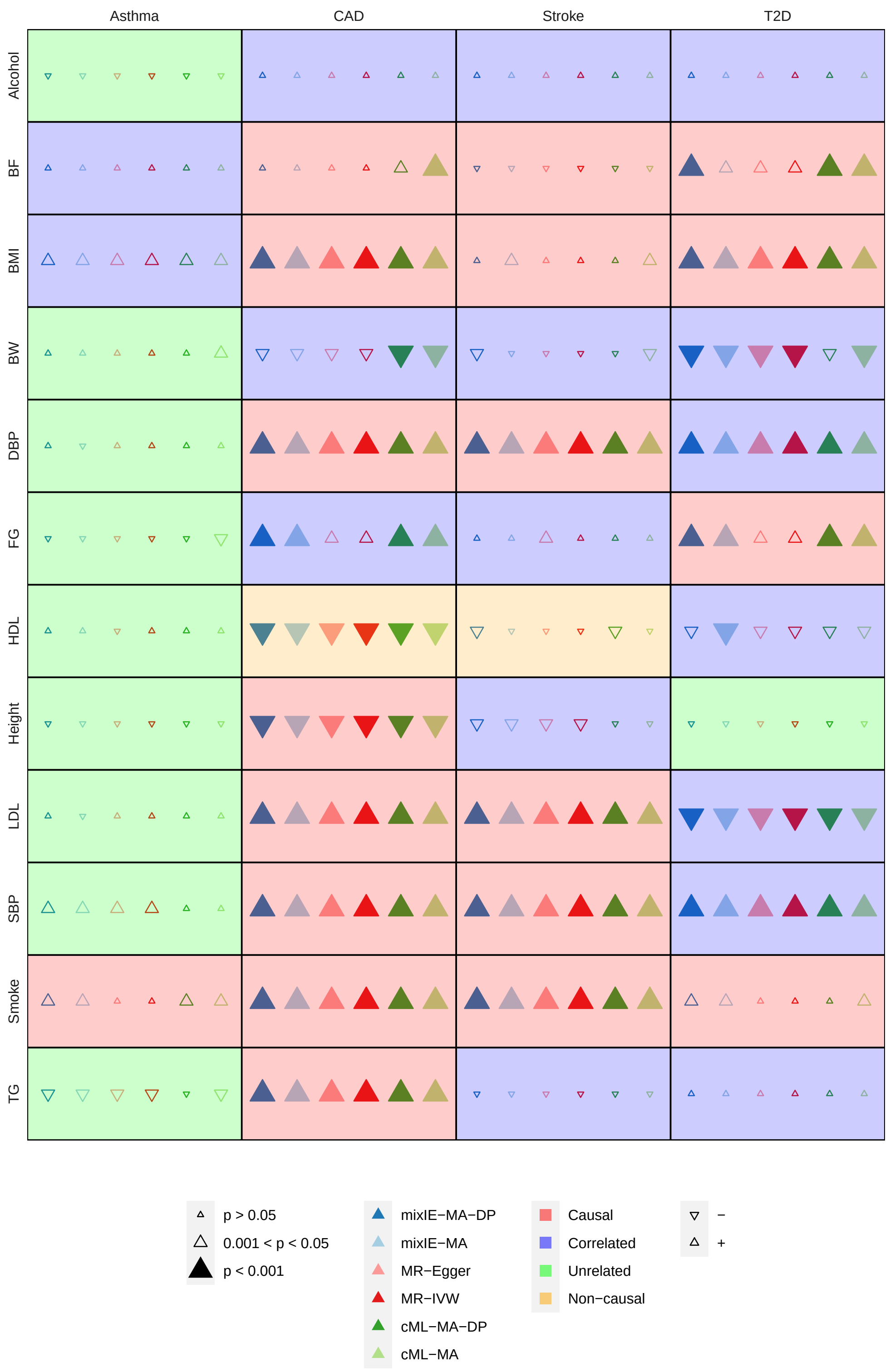

Fig L. Apply mixIE-MA, mixIE-MA-DP and Egger regression without re-orientating SNPs.

D Secondary Real Data Analysis

Table AI. Results for secondary real data analysis (63 pairs)

exposure	outcome	mixIE-MA_est	mixIE-MA_se	mixIE-MA_pval	mixIE-MA-DP_est	mixIE-MA-DP_se	mixIE-MA-DP_pval
Fasting_Proinsulin	Age_at_Smoking	-1.91e-02	1.59e-02	2.29e-01	-1.81e-02	1.50e-02	2.28e-01
Fasting_Proinsulin	Anorexia	9.16e-02	1.77e-01	6.05e-01	9.79e-02	1.92e-01	6.11e-01
Fasting_Proinsulin	Childhood_IQ	-1.30e-01	9.90e-02	1.89e-01	-1.26e-01	1.05e-01	2.26e-01
Fasting_Proinsulin	Ever_Never_Smoked	-4.35e-02	7.73e-02	5.74e-01	-4.17e-02	8.29e-02	6.15e-01
Fasting_Proinsulin	Former_Current_Smoker	-1.76e-01	1.09e-01	1.07e-01	-1.74e-01	1.17e-01	1.36e-01
Fasting_Proinsulin	Height_GIANT	6.44e-02	1.96e-02	9.99e-04	6.49e-02	2.07e-02	1.76e-03
Fasting_Proinsulin	HOMA-B	1.47e-01	1.03e-01	1.55e-01	1.51e-01	1.72e-01	3.79e-01
Fasting_Proinsulin	Infant_Head_Circumference	-7.33e-02	9.60e-02	4.45e-01	-7.40e-02	1.10e-01	5.00e-01
Fasting_Proinsulin	LDL	6.70e-03	4.62e-02	8.85e-01	2.41e-02	2.90e-02	4.05e-01
Fasting_Proinsulin	RA	-1.02e-01	9.70e-02	2.91e-01	-9.67e-02	1.18e-01	4.12e-01
Fasting_Proinsulin	SCZ	-1.12e-01	6.56e-02	8.79e-02	-1.10e-01	7.15e-02	1.25e-01
Fasting_Proinsulin	T2D	-8.06e-01	2.14e+00	7.07e-01	-6.03e-01	2.46e+00	8.06e-01
Height_GIANT	Age_at_Smoking	2.25e-03	5.00e-03	6.53e-01	2.11e-03	5.04e-03	6.76e-01
Height_GIANT	Ever_Never_Smoked	-2.82e-02	2.60e-02	2.78e-01	-3.09e-02	2.62e-02	2.37e-01
Height_GIANT	Fasting_Proinsulin	1.46e-02	1.55e-02	3.46e-01	1.34e-02	1.48e-02	3.64e-01
Height_GIANT	Former_Current_Smoker	-1.39e-02	3.59e-02	6.98e-01	-9.21e-03	3.57e-02	7.96e-01
Height_GIANT	HOMA-B	-8.59e-03	7.47e-03	2.50e-01	-8.81e-03	7.25e-03	2.24e-01
Height_GIANT	RA	1.13e-01	3.26e-02	5.30e-04	6.67e-02	3.72e-02	7.29e-02
Height_GIANT	SCZ	-1.60e-01	2.60e-02	6.87e-10	-3.40e-02	9.12e-02	7.09e-01
Height_GIANT	T2D	1.16e-01	4.02e-02	3.94e-03	5.69e-02	1.59e-01	7.21e-01
HOMA-B	Age_at_Smoking	-5.26e-02	5.45e-02	3.34e-01	-5.26e-02	5.45e-02	3.34e-01
HOMA-B	Ever_Never_Smoked	1.15e-01	2.78e-01	6.79e-01	1.15e-01	2.78e-01	6.79e-01
HOMA-B	Fasting_Proinsulin	2.40e-01	1.69e-01	1.55e-01	2.40e-01	1.69e-01	1.55e-01
HOMA-B	Former_Current_Smoker	8.75e-01	3.81e-01	2.17e-02	8.75e-01	3.81e-01	2.17e-02
HOMA-B	Height_GIANT	-1.52e-01	7.06e-02	3.16e-02	-1.52e-01	7.06e-02	3.16e-02
HOMA-B	Infant_Head_Circumference	4.59e-01	3.39e-01	1.76e-01	4.59e-01	3.39e-01	1.76e-01
HOMA-B	LDL	-2.70e-02	9.40e-02	7.74e-01	-2.70e-02	9.40e-02	7.74e-01
HOMA-B	RA	3.39e-01	3.91e-01	3.86e-01	3.39e-01	3.91e-01	3.86e-01
HOMA-B	SCZ	2.73e-01	2.58e-01	2.90e-01	2.73e-01	2.58e-01	2.90e-01
HOMA-B	T2D	3.44e-01	3.96e-01	3.84e-01	3.44e-01	3.96e-01	3.84e-01
LDL	Age_at_Smoking	-1.24e-02	7.06e-03	7.95e-02	-1.23e-02	7.32e-03	9.23e-02
LDL	Anorexia	8.13e-02	1.01e-01	4.19e-01	8.22e-02	1.07e-01	4.41e-01
LDL	Ever_Never_Smoked	-7.02e-03	3.69e-02	8.49e-01	-3.72e-02	4.14e-02	3.69e-01
LDL	Fasting_Proinsulin	-5.83e-02	2.24e-02	9.19e-03	-6.00e-02	2.22e-02	6.96e-03
LDL	Former_Current_Smoker	4.96e-02	4.95e-02	3.16e-01	4.95e-02	4.92e-02	3.15e-01
LDL	HOMA-B	1.65e-02	1.02e-02	1.04e-01	7.60e-03	1.37e-02	5.78e-01
LDL	Infant_Head_Circumference	-4.34e-02	4.35e-02	3.18e-01	-4.32e-02	4.82e-02	3.70e-01
LDL	RA	-9.12e-04	3.27e-02	9.78e-01	7.44e-03	3.45e-02	8.29e-01
LDL	SCZ	-7.30e-04	2.34e-02	9.75e-01	-1.73e-03	2.59e-02	9.47e-01
LDL	T2D	-2.62e-01	5.70e-02	4.18e-06	-1.70e-01	8.33e-02	4.11e-02
RA	SCZ	2.73e-02	1.23e-02	2.67e-02	1.96e-02	1.81e-02	2.78e-01
SCZ	Age_at_Smoking	-3.62e-03	5.51e-03	5.11e-01	-3.87e-03	5.26e-03	4.62e-01
SCZ	Childhood_IQ	-3.06e-02	4.26e-02	4.72e-01	-3.01e-02	4.55e-02	5.09e-01
SCZ	Fasting_Proinsulin	-1.21e-02	1.98e-02	5.41e-01	-5.82e-03	2.18e-02	7.89e-01
SCZ	Former_Current_Smoker	-1.08e-02	3.92e-02	7.82e-01	-7.01e-03	5.06e-02	8.90e-01
SCZ	Height_GIANT	-1.59e-02	1.24e-02	2.00e-01	-1.51e-02	1.64e-02	3.59e-01
SCZ	HOMA-B	9.76e-03	8.03e-03	2.24e-01	9.44e-03	7.67e-03	2.18e-01
SCZ	Infant_Head_Circumference	8.57e-03	3.57e-02	8.11e-01	9.65e-04	6.40e-02	9.88e-01
SCZ	LDL	3.74e-02	1.57e-02	1.69e-02	3.30e-03	2.56e-02	8.97e-01
SCZ	RA	-8.95e-03	2.94e-02	7.60e-01	2.46e-02	3.01e-02	4.14e-01
SCZ	T2D	1.67e-02	4.89e-02	7.33e-01	1.37e-02	7.07e-02	8.47e-01
T2D	Age_at_Smoking	-1.66e-03	5.82e-03	7.75e-01	-1.46e-03	6.40e-03	8.20e-01
T2D	Anorexia	1.29e-02	9.83e-02	8.95e-01	1.17e-02	1.06e-01	9.12e-01
T2D	Childhood_IQ	1.60e-02	3.74e-02	6.69e-01	1.75e-02	3.73e-02	6.39e-01
T2D	Ever_Never_Smoked	1.31e-02	2.97e-02	6.59e-01	1.39e-02	3.30e-02	6.73e-01
T2D	Fasting_Proinsulin	2.72e-02	3.22e-02	3.98e-01	4.53e-02	5.47e-02	4.07e-01
T2D	Former_Current_Smoker	1.30e-02	4.06e-02	7.49e-01	1.42e-02	4.52e-02	7.52e-01
T2D	Height_GIANT	-9.05e-03	9.00e-03	3.15e-01	-1.11e-02	1.43e-02	4.38e-01
T2D	HOMA-B	-3.00e-02	6.70e-02	6.55e-01	-3.85e-02	2.65e-02	1.46e-01
T2D	Infant_Head_Circumference	-2.95e-02	3.61e-02	4.13e-01	-3.20e-02	6.43e-02	6.19e-01
T2D	LDL	5.75e-03	9.53e-03	5.46e-01	6.67e-03	1.38e-02	6.29e-01
T2D	RA	-3.84e-02	3.30e-02	2.44e-01	-3.74e-02	3.88e-02	3.35e-01
T2D	SCZ	-5.66e-02	3.00e-02	5.90e-02	-5.83e-02	3.15e-02	6.42e-02

E Computational time

We compared the computational time of our proposed mixIE method with other MR methods used in the simulation. We used all default parameters provided by each method. In particular, for mixIE-MA, we used 50 random starting points, and for mixIE-MA-DP, we used $B = 200$ data perturbations and 50 random starting points. For cML-MA-DP, we used 200 data perturbations and 0 random starting points (in addition to the single default starting point).

We generated data according to the directional pleiotropy and InSIDE satisfied setup in Section 2.7.1 in the Main text. Specifically, we tried $m = 10, 30, 100, n = 50,000$ and 50% invalid IVs. For each m, we did 10 simulations and averaged the computational time among the 10 simulations for each method. Results are shown in Table AJ. All methods were run on a 2017 MacBook Pro with a 3.1 GHz Intel Core i5 processor and 8 GB memory.

Table AJ. Comparison of computational times (in seconds) of different MR methods as averaged over 10 simulations for m IVs.

Method \ m	10	30	100
Egger	0.001	0.001	0.001
IVW	0.002	0.001	0.001
Weighted-median	0.430	0.520	0.645
MR-ContMix	0.002	0.007	0.032
MRMix	2.949	4.521	7.002
cML-MA	0.011	0.057	0.314
cML-MA-DP	1.921	12.134	71.418
mixIE-MA	0.079	0.111	0.192
mixIE-MA-DP	15.957	22.837	35.811

References

1. Louis TA. Finding the observed information matrix when using the EM algorithm. Journal of the Royal Statistical Society: Series B (Methodological). 1982;44(2):226–233.

2. Xue H, Shen X, Pan W. Constrained maximum likelihood-based Mendelian randomization robust to both correlated and uncorrelated pleiotropic effects. The American

Journal of Human Genetics. 2021;108(7):1251–1269.

3. Morrison J, Knoblauch N, Marcus JH, Stephens M, He X. Mendelian randomization accounting for correlated and uncorrelated pleiotropic effects using genome-wide summary statistics. Nature genetics. 2020;52(7):740–747.
